# 3,3′-((3-Hydroxyphenyl)azanediyl)dipropionic Acid Derivatives as a Promising Scaffold Against Drug-Resistant Pathogens and Chemotherapy-Resistant Cancer

**DOI:** 10.3390/pathogens14050484

**Published:** 2025-05-15

**Authors:** Povilas Kavaliauskas, Waldo Acevedo, Eglė Mickevičiūtė, Ramunė Grigalevičiūtė, Birutė Grybaitė, Birutė Sapijanskaitė-Banevič, Guoda Pranaitytė, Vidmantas Petraitis, Rūta Petraitienė, Vytautas Mickevičius

**Affiliations:** 1 Department of Organic Chemistry, Kaunas University of Technology, LT-50254 Kaunas, Lithuania or povilas.kavaliauskas@som.umaryland.edu (P.K.); birute.grybaite@ktu.lt (B.G.); birute.sapijanskaite@ktu.lt (B.S.-B.); guoda.pranaityte@ktu.edu (G.P.); 2 Biological Research Center, Lithuanian University of Health Sciences, Tilzes Street 18, LT-47181 Kaunas, Lithuania; ramune.grigaleviciute@lsmuni.lt; 3 Department of Microbiology and Immunology, University of Maryland School of Medicine, 655 W. Baltimore Street, Baltimore, MD 21201, USA; 4 Institute of Infectious Diseases and Pathogenic Microbiology, Birstono Street 38A, LT-59116 Prienai, Lithuania; 5 Instituto de Química, Facultad de Ciencias, Pontificia Universidad Católica de Valparaíso, Valparaíso 2373223, Chile; waldo.acevedo@pucv.cl; 6 Department of Information Systems, Kaunas University of Technology, LT-51368 Kaunas, Lithuania; egle.mickeviciute@ktu.lt; 7 Department of Animal Nutrition, Lithuanian University of Health Sciences, Tilzes Street 18, LT-47181 Kaunas, Lithuania; 8 Center for Discovery and Innovation, Hackensack Meridian Health, Nutley, NJ 07110, USA; vidmantas.petraitis@hmh-cdi.org (V.P.); ruta.petraitiene@hmh-cdi.org (R.P.)

**Keywords:** 3,3′-((3-hydroxyphenyl)azanediyl)dipropionic acid, *β*-amino acids derivatives, antimicrobial activity, multidrug-resistant pathogens, head and neck cancer, c-MET, HER2

## Abstract

The synthesis and antimicrobial and anticancer activity of 3,3′-((3-hydroxyphenyl)azanediyl)dipropionic acid derivatives (**2**–**25**) against drug-resistant bacterial pathogens and FaDu head and neck cancer cells were investigated. The derivatives were synthesized through various methods, including esterification, hydrazinolysis, and condensation reactions. The compounds demonstrated structure-dependent antimicrobial activity, predominantly targeting Gram-positive pathogens. Compounds containing 4-nitrophenyl, 1-naphthyl, and 5-nitro-2-thienyl groups exhibited enhanced activity against *S. aureus* and *E. faecalis*. Additionally, compounds **5**, **6**, and **25** showed antiproliferative activity in cisplatin-resistant FaDu cells at low micromolar concentrations. The in silico modeling revealed that compound **25** interacts with the HER-2 and c-MET proteins. These compounds also induced significant oxidative stress in FaDu cells and demonstrated low cytotoxic activity in non-cancerous HEK293 cells. These results highlight the potential of *N*-aryl-substituted *β*-amino acid derivatives as promising scaffolds for the further development of novel amino acid-based antimicrobial and anticancer agents targeting drug-resistant pathogens and cancers.

## 1. Introduction

The growing healthcare problem of antimicrobial resistance (AMR) and the microbial acquisition of multiple genetic resistance determinants have facilitated the emergence of multidrug-resistant (MDR) bacterial pathogens with extremely limited therapeutic options, in particular in patients with innate or acquired immunodeficiencies, chronic traumas, or undergoing chemotherapy [1]. Among these drug-resistant pathogens, ESKAPE group pathogens (*Enterococcus faecium*, *Staphylococcus aureus*, *Klebsiella pneumoniae*, *Acinetobacter baumannii*, *Pseudomonas aeruginosa*, and *Enterobacter* spp.) present a significant global health challenge due to their ability to evade conventional antimicrobial agents through diverse resistance mechanisms, including enzymatic degradation, efflux pump overexpression, and target site modification [2,3]. While *E. faecium* is widely recognized for its multidrug-resistant phenotype, *Enterococcus faecalis* also represents a clinically relevant pathogen, frequently isolated from hospital-acquired infections and known to harbor resistance to aminoglycosides and beta-lactams. Its prevalence in clinical settings and well-characterized pathogenicity make *E. faecalis* a suitable and representative model for initial antimicrobial screening [2,3,4,5]. The rapid acquisition of resistance in the clinical setting and loss in the therapeutic activity of existing antibiotics necessitates the development of novel pharmacophores capable of overcoming these resistance mechanisms and restoring therapeutic efficacy by targeting novel cellular targets [4,5].

In parallel, resistance to chemotherapeutic agents remains a huge therapeutic obstacle in clinical oncology, particularly in head and neck squamous cell carcinoma (HNSCC), which accounts for a substantial burden of cancer-related morbidity and mortality [6,7,8]. HNSCC frequently exhibits resistance to standard treatment modalities, including cisplatin-based chemotherapy and radiation therapy, due to mechanisms such as increased drug efflux, enhanced DNA repair, epithelial-to-mesenchymal transition, and evasion of apoptosis [9,10,11,12]. The recurrence and progression of drug-resistant HNSCC underscore the urgent need for the identification of novel chemotherapeutic scaffolds capable of targeting these resistance pathways while minimizing systemic toxicity [11,12].

Naturally occurring *β*-alanine-containing compounds have demonstrated significant biological activity, highlighting their potential as pharmacological scaffolds [13]. Carnosine and anserine, found in mammalian tissues, possess free radical-scavenging properties and confer cellular protection against oxidative stress [14]. Moreover, the cyclization products of *β*-alanine derivatives play critical roles in biological systems, serving as key intermediates in the synthesis of structurally diverse heterocyclic frameworks with potential therapeutic applications [15,16].

**Figure 1:**
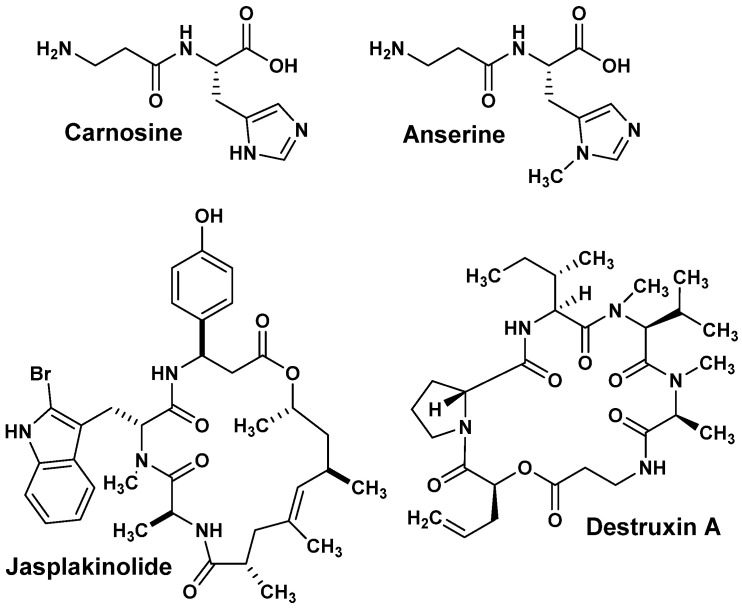
Derivatives of *β*-amino acids.

*N*-substituted *β*-amino acids are widely utilized as synthetic building blocks for the development of bioactive molecules, including azetidine, dihydroquinolinone, hydropyridone, benzodiazepine, and imidazole derivatives [17,18,19,20,21]. Notably, *β*-amino acids are frequently incorporated into bacterial secondary metabolites, conferring antimicrobial properties. For example, cryptophycin, a depsipeptide derived from *Nostoc* sp. GSV224, exhibits potent antitumor activity and has been investigated for cancer therapy [22]. Similarly, destruxins, fungus-derived cyclic depsipeptides, have demonstrated cytotoxic activity, with potential applications in leukemia and hepatitis B treatment [23]. The amino acid derivative Jasplakinolide inhibits the proliferation of PC3 prostate carcinoma cells by binding F-actin [24] (Figure 1).

Beyond their antimicrobial and anticancer potential, *β*-amino acid-derived hydrazides and hydrazones of carboxylic acids serve as precursors for the synthesis of nitrogen-containing heterocycles, which have been extensively explored in medicinal chemistry [25,26]. These heterocyclic frameworks have demonstrated efficacy in diverse applications, including pesticide development, dye synthesis, antioxidant formulation, corrosion inhibition, and the treatment of cancer, tuberculosis, and various microbial and viral infections [27,28,29,30,31,32,33].

In this study, we synthesized and characterized a series of novel *N*-aryl-substituted *β*-amino acid derivatives bearing a 3-hydroxyphenyl core and evaluated their in vitro activity against MDR bacterial pathogens and drug-resistant cancer FaDu cells. Our findings demonstrate that these compounds exhibit promising antimicrobial activity against *E. faecium* and MDR *S. aureus*, particularly those harboring emerging AMR determinants. Furthermore, by using radiation- and cisplatin-resistant FaDu head and neck cancer cells, we show that these derivatives induce cytotoxicity at low micromolar concentrations. This study demonstrates that *N*-aryl-substituted *β*-amino acid derivatives could be further explored as promising pharmacophores with dual antimicrobial and anticancer activity leading to further hit-to-lead optimization.

## 2. Materials and Methods

### 2.1. Chemical Synthesis

Reagents and solvents were purchased from Sigma-Aldrich (St. Louis, MO, USA) and used without further purification. The reaction course and purity of the synthesized compounds were monitored by TLC by using aluminum plates pre-coated with silica gel with F_254_ nm (Merck KGaA, Darmstadt, Germany). NMR spectra were recorded with a Brucker Avance III (400, 101 MHz) spectrometer (Bruker BioSpin AG, Fällanden, Switzerland). Chemical shifts were reported in (δ) ppm relative to tetramethylsilane (TMS), with the residual solvent as the internal reference (DMSO-d_6_, δ = 2.50 ppm for ^1^H and δ = 39.5 ppm for ^13^C). Data are reported as follows: chemical shift, multiplicity, coupling constant [Hz], integration, and assignment. Melting points were determined with a B-540 melting point analyzer (Büchi Corporation, New Castle, DE, USA) and were uncorrected. Mass spectra were measured with a Bruker maXis 4G mass spectrometer (Bruker Daltonics, Bremen, Germany). Elemental analyses (C, H, and N) were conducted by using the Elemental Analyzer CE-440 (Exeter Analytical Ltd., Coventry, UK), and their results were found to be in good agreement (±0.3%) with the calculated values. IR spectra (ν, cm^−1^) were recorded with a Perkin–Elmer Spectrum BX FT–IR spectrometer (Perkin–Elmer Inc., Waltham, MA, USA) using KBr pellets.

#### 2.1.1. 3,3′-((3-Hydroxyphenyl)azanediyl)dipropionic Acid (**2**)

A mixture of *m*-aminophenol (5.45 g, 50 mmol), acrylic acid (10.81 g, 150 mmol), and water (5 mL) was heated to 75 °C for 5 h and cooled. Crystalline product 2 was filtered off, washed with diethyl ether, dried, and recrystallized from the water and ethanol mixture. Light brown powder, yield 18.97 g (75%), m.p. 150–152 °C (water/ethanol) [2]. ^1^H NMR (400 MHz, DMSO-d_6_) δ: 2.44 (t, *J* = 7.1 Hz, 4H, 2× CH_2_CO), 3.49 (t, *J* = 7.1 Hz, 4H, 2× NCH_2_), 5.99–6.15 (m, 3H, H_Ar_), 6.94 (t, *J* = 8.0 Hz, 1H H_Ar_), 9.02 (s, 1H, OH), 12.25 (s, 2H, COOH). ^13^C NMR (101 MHz, DMSO-d_6_) δ: 32.05 (CH_2_CO), 46.40 (NCH_2_), 99.11, 103.31, 103.63, 129.99, 148.21, 158.53 (C_Ar_), 173.30 (C=O). IR (KBr): ν_max_ (cm^−1^) = 3323 (OH), 1662 (C=O). Anal. Calcd. for C_12_H_15_NO_5_, %: C 56.91; H 5.97; N 5.53. Found: C 56.75; H 5.72; N 5.33.

#### 2.1.2. Dimethyl 3,3′-((3-hydroxyphenyl)azanediyl)dipropionate (**3**)

A mixture of dipropionic acid 2 (0.51 g, 2 mmol), conc. sulfuric acid (1 mL), and methanol (7 mL) was heated under reflux for 7 h. Then, the solvent was evaporated under reduced pressure, and the residue neutralized with 5% sodium carbonate solution to pH 7. The obtained solid was filtered off, washed with plenty of water, and recrystallized from propan-2-ol. Light brown powder, yield 0.31 g (56%), m.p. 155–157 °C [27]. ^1^H NMR (400 MHz, DMSO-d_6_) δ: 2.53 (overlaps with DMSO-d_6_, 4H, 2× CH_2_CO), 3.51 (t, *J* = 7.1 Hz, 4H, 2× NCH_2_), 3.60 (s, 6H, 2× CH_3_), 6.03–6.14 (m, 3H, H_Ar_), 6.94 (t, *J* = 8.4 Hz, 1H, H_Ar_), 9.05 (s, 1H, OH); ^13^C NMR (101 MHz, DMSO-d_6_) δ: 31.70 (CH_2_CO), 46.25 (NCH_2_), 51.40 (CH_3_), 99.23, 103.38, 103.83, 129.93, 148.00, 158.48 (C_Ar_), 172.07 (2× C=O). IR (KBr): ν_max_ (cm^−1^) = 3311 (OH), 1711 (2× C=O). Anal. Calcd. for C_14_H_19_NO_5_, %: C 59.78; H 6.81; N 4.98. Found: C 59.57; H 6.54; N 4.69.

#### 2.1.3. 3,3′-((3-Hydroxyphenyl)azanediyl)di(propanehydrazide) (**4**)

A mixture of methyl ester 3 (0.32 g, 1.1 mmol), hydrazine hydrate (1.31 g, 26 mmol), and propan-2-ol (10 mL) was heated under reflux for 6 h and cooled. Crystalline product 4 was filtered off, washed with propan-2-ol, diethyl ether, and dried. White powder, yield 0.17 g (56%), m.p. 160–162 °C (from propan-2-ol) [28]. ^1^H NMR (400 MHz, DMSO-d_6_) δ: 2.25 (t, *J* = 7.1 Hz, 4H, 2× CH_2_CO), 3.44 (t, *J* = 7.1 Hz, 4H, 2× NCH_2_), 4.20 (s, 4H, 2× NH_2_), 5.97–6.17 (m, 3H, H_Ar_), 6.92 (t, *J* = 8.0 Hz, 1H, H_Ar_), 9.04 (s, 3H, OH, 2× NH); ^13^C NMR (101 MHz, DMSO-d_6_) δ: 31.57 (CH_2_CO), 46.92 (NCH_2_), 98.97, 103.21, 103.34, 129.94, 148.40, 158.46 (C_Ar_), 170.18 (2× C=O). IR (KBr): ν_max_ (cm^−1^) = 3285 (OH), 3173 (NH_2_), 3053 (NH), 1671, 1623 (C=O). Anal. Calcd. for C_12_H_19_N_5_O_3_, %: C 51.23; H 6.81; N 24.90. Found: C 51.07; H 6.57; N 24.65.

#### 2.1.4. 1-(3-Hydroxyphenyl)dihydropyrimidine-2,4(1*H*,3*H*)-dione (**5**)

It was resynthesized according to the described procedure [34].

#### 2.1.5. 1-(3-Hydroxyphenyl)-2-thioxotetrahydropyrimidin-4(1*H*)-one (**6**)

It was resynthesized according to the described procedure [34].

#### 2.1.6. 3-(7-Hydroxy-4-oxo-3,4-dihydroquinolin-1(2*H*)-yl)propanoic Acid (**7**)

It was resynthesized according to the described procedure [35].

#### 2.1.7. General Procedure for Preparation of Hydrazones **8**–**18**

To a solution of hydrazide 4 (1.5 mmol) in propan-2-ol (15 mL), the corresponding aromatic aldehyde was added (1.65 mmol), and the mixture was heated under reflux for 2 h; then, it was cooled down, and the formed precipitate was filtered off, washed with methanol and diethyl ether, and recrystallized from 1,4-dioxane or propan-2-ol.

#### 2.1.8. 3,3′-((3-Hydroxyphenyl)azanediyl)bis(*N*′-(benzylidene)propanehydrazide) (**8**)

Light purple powder, yield 0.41 g (59%), m.p. 173–175 °C (from propan-2-ol). ^1^H NMR (400 MHz, DMSO-d_6_) δ: 2.81–2.98 and 3.28–3.43 (2m, 4H, 2× CH_2_CO), 3.51–3.71 (m, 4H, 2× NCH_2_), 6.02–8.21 (m, 16H, H_Ar_, 2× CH), 9.09 (s, 1H, OH), 11.37, 11.46 (2s, 2H, 2× NH); ^13^C NMR (101 MHz, DMSO-d_6_) δ: 30.33, 30.49, 32.36, 32.55 (CH_2_CO), 46.36, 46.69 (NCH_2_), 98.86, 99.02, 103.08, 103.20, 103.39, 103.47, 126.71, 126.74, 127.00, 128.77, 128.80, 129.68, 129.88, 129.91, 134.16, 143.10, 143.13, 146.10, 146.12, 148.38, 158.54, 158.58, 167.20 (C_Ar_), 172.92 (C=O). IR (KBr): ν_max_ (cm^−1^) = 3197 (OH), 3059, 2962 (2× NH), 1655 (C=O). Anal. Calcd. for C_26_H_27_N_5_O_3_, %: C 68.25; H 5.95; N 15.31. Found: C 68.00; H 5.63; N 15.11. HRMS *m*/*z* calculated for C_26_H_27_N_5_O_3_ [M+H]+: 458.2113; found: 458.2187.

#### 2.1.9. 3,3′-((3-Hydroxyphenyl)azanediyl)bis(*N*′-(2,4-difluorobenzylidene)propanehydrazide) (**9**)

Light purple powder, yield 0.59 g (74%), m.p. 223–225 °C (from propan-2-ol). ^1^H NMR (400 MHz, DMSO-d_6_) δ: 2.43–2.53 (m, overlaps with DMSO-d_6_, 2H, CH_2_CO), 2.78–2.93 (m, 2H, CH_2_CO), 3.48–3.68 (m, 4H, 2× NCH_2_), 6.04–7.95 (m, 10H, H_Ar_), 8.10, 8.12, 8.28, 8.31 (4s, 2H, 2× CH), 9.05 (br. s, 1H, OH), 11.46, 11.56 (2s, 2H, 2× NH); ^13^C NMR (101 MHz, DMSO-d_6_) δ: 30.37, 30.52, 32.48, 32.76 (CH_2_CO), 46.52, 46.90 (NCH_2_), 98.93, 99.10, 103.20, 103.33, 103.53, 103.59, 104.20, 104.46, 104.72, 112.49, 112.67, 118.64, 118.73, 127.73, 127.84, 128.03, 130.03, 135.20, 138.19, 148.45, 148.55, 158.66, 159.61, 162.12, 167.42 (C_Ar_), 173.17 (C=O). IR (KBr): ν_max_ (cm^−1^) = 3449 (OH), 3166, 3083 (2× NH), 1678 (C=O). Anal. Calcd. for C_26_H_23_F_4_N_5_O_3_, %: C 58.98; H 4.38; N 13.23. Found: C 58.71; H 4.13; N 13.02. HRMS *m*/*z* calculated for C_26_H_23_F_4_N_5_O_3_ [M+H]+: 530.1737; found: 530.1810.

#### 2.1.10. 3,3′-((3-Hydroxyphenyl)azanediyl)bis(*N*′-(4-nitrobenzylidene)propanehydrazide) (**10**)

Ruby powder, yield 0.56 g (68%), m.p. 231–233 °C (from propan-2-ol). ^1^H NMR (400 MHz, DMSO-d_6_) δ: 2.48–2.58 (m, overlaps with DMSO-d_6_, 2H, CH_2_CO), 2.87–2.96 (m, 2H, CH_2_CO), 3.52–3.67 (m, 4H, 2× NCH_2_), 6.05–8.30 (m, 14H, H_Ar_, 2× CH), 9.08, 9.10 (2s, 1H, OH), 11.66, 11.73, (2s, 2H, 2× NH); ^13^C NMR (101 MHz, DMSO-d_6_) δ: 30.73, 32.91 (CH_2_CO), 46.53 (NCH_2_), 99.01, 99.16, 103.23, 103.36, 103.586, 103.63, 124.04, 124.10, 124.39, 127.69, 127.98, 128.02, 130.06, 140.54, 140.57, 140.84, 143.75, 147.62, 148.55, 158.68, 158.72, 167.81, 167.85 (C_Ar_), 173.52 (C=O). IR (KBr): ν_max_ (cm^−1^) = 3415 (OH), 3112, 2961 (NH), 1681 (C=O). Anal. Calcd. for C_26_H_25_N_7_O_7_, %: C 57.04; H 4.60; N 17.91. Found: C 56.79; H 4.42; N 17.71. HRMS *m*/*z* calculated for C_26_H_25_N_7_O_7_ [M+H]+: 548.1815; found: 548.1886.

#### 2.1.11. 3,3′-((3-Hydroxyphenyl)azanediyl)bis(*N*′-(4-chlorobenzylidene)propanehydrazide) (**11**)

Pink powder, yield 0.41 g (52%), m.p. 186–188 °C (from dioxane). ^1^H NMR (400 MHz, DMSO-d_6_) δ: 2.48–2.55 (m, overlaps with DMSO-d_6_, 2H, CH_2_CO), 2.83–2.93 (m, 2H, CH_2_CO), 3.49–3.67 (m, 4H, 2× NCH_2_), 6.05–8.15 (m, 14H, H_Ar_, 2× CH), 9.06 (s, 1H, OH), 11.41, 11.42, 11.49, (3s, 2H, 2× NH); ^13^C NMR (101 MHz, DMSO-d_6_) δ: 30.28, 30.46, 32.56 (CH_2_CO), 46.41, 46.69 (NCH_2_), 98.84, 99.00, 103.07, 103.21, 103.40, 103.47, 128.34, 128.63, 128.83, 129.91, 133.09, 133.24, 134.09, 134.36, 141.81, 144.79, 148.37, 148.46, 158.55, 158.58, 167.31 (C_Ar_), 173.02 (C=O). IR (KBr): ν_max_ (cm^−1^) = 3255 (OH), 3179, 3057 (NH), 1660 (C=O). Anal. Calcd. for C_26_H_25_Cl_2_N_5_O_3_, %: C 59.32; H 4.79; N 13.30. Found: C 59.12; H 4.53; N 13.12. HRMS *m*/*z* calculated for C_26_H_25_Cl_2_N_5_O_3_ [M+H]+: 526.1334; found: 526.1405.

#### 2.1.12. 3,3′-((3-Hydroxyphenyl)azanediyl)bis(*N*′-(4-dimethylamino)benzylidene)propanehydrazide) (**12**)

Light brown powder, yield 0.25 g (30%), m.p. 138–140 °C (from a mixture of propan-2-ol and dioxane). ^1^H NMR (400 MHz, DMSO-d_6_) δ: 2.40–2.50 (m, overlaps with DMSO-d_6_, 2H, CH_2_CO), 2.79–2.91 (m, 2H, CH_2_CO), 2.91, 2.92, 2.95 (3s, 12H, 4× CH_3_), 3.51–3.64 (m, 4H, 2× NCH_2_), 6.05–7.51 (m, 12H, H_Ar_), 7,86, 7.99 (2s, 2H, 2× CH), 9.05 (br. s, 1H, OH), 11.06 11.07, 11.12, 11.13 (4s, 2H, 2× NH); ^13^C NMR (101 MHz, DMSO-d_6_) δ: 25.29 (4CH_3_), 30.22, 30.34, 32.16 32.27 (CH_2_CO), 46.12, 46.54, 46.71 (NCH_2_), 98.60, 98.75, 102.87, 102.98, 103.08, 103.18, 111.55, 111.61, 121.36, 121.39, 121.41, 127.76, 127.80, 128.10, 129.62, 129.66, 143.71, 143.78, 146.68, 148.24, 148.30, 151.02, 151.20, 158.32, 166.29, 166.33, 169.78 (C_Ar_), 172.05 (C=O). IR (KBr): ν_max_ (cm^−1^) = 3320 (OH), 3182, 3079 (NH), 1649 (C=O). Anal. Calcd. for C_30_H_37_N_7_O_3_, %: C 66.28; H 6.86; N 18.03. Found: C 66.00; H 6.64; N 17.82. HRMS *m*/*z* calculated for C_30_H_37_N_7_O_3_ [M+H]+: 544.2957; found: 544.3030.

#### 2.1.13. 3,3′-((3-Hydroxyphenyl)azanediyl)bis(*N*′-(3,4,5-trimethoxybenzylidene)propanehydrazide) (**13**)

Light pink powder, yield 0.48 g (50%), m.p. 189–191 °C (from propan-2-ol). ^1^H NMR (400 MHz, DMSO-d_6_) δ: 2.50 (overlaps with DMSO-d_6_) and 2.83–2.97 (m, 4H, 2CH_2_CO), 3.49–3.65 (m, 4H, 2× NCH_2_), 3.67–3.90 (m, 18H, 6× OCH_3_), 6.02–6.27 (m, 3H, H_Ar_), 6.79–7.28 (m, 5H, H_Ar_), 7.86, 7.91, 8.05, 8.07 (4s, 2H, 2× CH), 9.02, 9.04, 9.07 (3s, 1H, OH), 11.38, 11.39, 11.40 (3s, 2H, 2× NH); ^13^C NMR (101 MHz, DMSO-d_6_) δ: 30.26, 30.39, 32.37, 32.52 (CH_2_CO), 46.48, 46.60 (NCH_2_), 55.77, 55.87, 55.90, 55.93, 56.06, 60,07, 60,10, 60.22, 62.03, 99.00, 99.13, 103.32, 103.47, 103.81, 103.96, 104.20, 106.73, 129.66, 129.72, 129.79, 131.66, 138.85, 138.92, 139.07, 142.82, 142.92, 142.98, 146.12, 148.40, 148.47, 153.06, 153.14, 153.33, 158.49, 167.13 (C_Ar_) 172.86, 172.92 (C=O). IR (KBr): ν_max_ (cm^−1^) = 3200 (OH), 2940, 2838 (NH), 1657 (C=O). Anal. Calcd. for C_32_H_39_N_5_O_9_, %: C 60.27; H 6.16; N 10.98. Found: C 60.05; H 5.97; N 10.74. HRMS *m*/*z* calculated for C_32_H_39_N_5_O_9_ [M+Na]^+^: 660.2747; found: 660.2643.

#### 2.1.14. 3,3′-((3-Hydroxyphenyl)azanediyl)bis(*N*′-(naphthalen-1-ylmethylene)propanehydrazide) (**14**)

Light violet powder, yield 0.4 g (48%), m.p. 134–136 °C (from propan-2-ol). ^1^H NMR (400 MHz, DMSO-d_6_) δ: 2.54–2.62 and 2.88–3.07 (2m, 4H, CH_2_CO) 3.60–3.80 (m, 4H, 2× NCH_2_), 6.00–6.38 (m, 3H, H_Ar_), 6.83–7.04 (m, 1H, H_Ar_), 7.40–8.08 (m, 12H, H_Ar_), 8.40–8.55 (m, 1H, H_Ar_), 8.58–8.89 (m, 3H, H_Ar_, 2× CH), 9.07, 9.08 (2s, 1H, OH), 11.42, 11.43, 11.55, 11.56 (4s, 2H, 2× NH); ^13^C NMR (101 MHz, DMSO-d_6_) δ: 30.40, 30.42, 30.54, 32.56 (CH_2_CO), 46.41, 46.71, 46.78 (NCH_2_), 99.03, 99.13, 103.37, 103.52, 123.36, 124.32, 125.54, 125.62, 126.14, 126.18, 126.26, 126.50, 127.14, 127.21, 127.29, 127.95, 128.78, 129.38, 129.43, 129.51, 129.92, 130.09, 130.43, 133.43, 133.45, 133.52, 142.44, 146.08, 148.40, 148.46, 158.56, 167.22 (C_Ar_), 172.91, 172.95 (C=O). IR (KBr): ν_max_ (cm^−1^) = 3178 (OH), 3055, 2958 (NH), 1664 (C=O). Anal. Calcd. for C_34_H_31_N_5_O_3_, %: C 73.23; H 5.60; N 12.56. Found: C 73.01; H 5.47; N 12.32.

#### 2.1.15. 3,3′-((3-Hydroxyphenyl)azanediyl)bis(*N*′-(furan-2-ylmethylene)benzylidene)propanehydrazide) (**15**)

Gray powder, yield 0.39 g (59%), m.p. Decomposition at 118 °C (from propan-2-ol). ^1^H NMR (400 MHz, DMSO-d_6_) δ: 2.40–2.52 (m, overlaps with DMSO-d_6_, 2H, CH_2_CO), 2.73–2.89 (m, 2H, CH_2_CO), 3.48–3.68 (m, 4H, 2× NCH_2_), 6.01–8.15 (m, 12H, H_Ar_, H_Het,_ 2× CH), 8.95, 9.02, 9.05 (3 s, 1H, OH), 11.30, 11.38 (2s, 2H, 2× NH); ^13^C NMR (101 MHz, DMSO-d_6_) δ: 30.38, 30.51, 32.34, 32.52 (CH_2_CO), 46.03, 46.73 (NCH_2_), 98.79, 98.96, 103.25, 103.31, 103.44, 103.52, 103.66, 112.02, 112.13, 112.92, 113.02, 113.25, 113.30, 129.96, 133.09, 133.13, 133.17, 136.02, 144.76, 144.80, 145.02, 149.25, 149.39, 158.49, 158.52, 167.18 (C_Ar_), 172.77, 172.79 (C=O). IR (KBr): ν_max_ (cm^−1^) = 3415 (OH), 3194, 3112 (NH), 1661 (C=O). Anal. Calcd. for C_22_H_23_N_5_O_5_, %: C 60.40; H 5.30; N 16.01. Found: C 60.20; H 5.17; N 15.82. HRMS *m*/*z* calculated for C_22_H_23_N_5_O_5_ [M+Na]^+^: 460.1699; found: 460.1594.

#### 2.1.16. 3,3′-((3-Hydroxyphenyl)azanediyl)bis(*N*′-((5-nitrothiophen-2-yl)methylene)propanehydrazide) (**16**)

Brown powder, yield 0.57 g (68%), m.p. Decomposition at 142 °C (from propan-2-ol). ^1^H NMR (400 MHz, DMSO-d_6_) δ: 2.77–2.91 (m, 4H, CH_2_CO), 3.50–3.69 (m, 4H, 2× NCH_2_), 6.19–6.34 (m, 2H, H_Ar_), 6.66–7.15 (m, 2H, H_Ar_), 7.92–8.42 (m, 4H, H_Ar_, CH), 9.25, 9.47 (2s, 1H, 2× CH), 10.03 (s, 1H, OH), 11.74, 11.75, 11.77, 11.78 (4s, 2H, 2× NH); ^13^C NMR (101 MHz, DMSO-d_6_) δ: 30.25, 32.33 (CH_2_CO), 46.13, 46.38 (NCH_2_), 96.07, 98.54, 98.84, 116.33, 117.50, 119.00, 122.80, 128.86, 130.14, 130.44, 135.73, 136.29, 139.66, 146.52, 146.77, 147.71, 148.36, 150.27, 150.64, 162.32, 167.76 (C_Ar_), 173.05, 173.22 (C=O). IR (KBr): ν_max_ (cm^−1^) = 3198 (OH), 3090, 3046 (NH), 1650 (C=O). Anal. Calcd. for C_22_H_21_N_7_O_7_S_2_, %: C 47.22; H 3.78; N 17.52. Found: C 47.07; H 3.51; N 17.32.

#### 2.1.17. 3,3′-((3-Hydroxyphenyl)azanediyl)bis(*N*′-(5-nitrofuran-2-yl)methylene)benzylidene)propanehydrazide) (**17**)

Dark green powder, yield 0.62 g (79%), m.p. 216–218 °C (from a mixture of propan-2-ol and dioxane). ^1^H NMR (400 MHz, DMSO-d_6_) δ: 2.45–2.59 (m, overlaps with DMSO-d_6_, 2H, CH_2_CO), 2.78–2.92 (m, 2H, CH_2_CO), 3.47–3.68 (m, 4H, 2× NCH_2_), 6.00–8.18 (m, 10H, H_Ar_, H_Het,_ 2× CH), 9.02, 9.04, 907 (3 s, 1H, OH), 11.73, 11.75, 11.82 (3s, 2H, 2× NH); ^13^C NMR (101 MHz, DMSO-d_6_) δ: 30.23, 30.45, 32.45, 32.69 (CH_2_CO), 46.06, 46.13, 46.51, 46.63 (NCH_2_), 98.93, 99.05, 103.21, 103.29, 103.47, 103.54, 114.25, 114.37, 114.62, 114.68, 115.03, 115.17, 129.99, 131.04, 131.19, 134.06, 148.24, 148.31, 148.36, 151.62, 151.69, 151.82, 158.53, 167.88, 167.93 (C_Ar_), 173.38, 173.40 (C=O). IR (KBr): ν_max_ (cm^−1^) = 3323 (OH), 3199, 3105 (NH), 1669 (C=O). Anal. Calcd. for C_22_H_21_N_7_O_9_, %: C 50.10; H 4.01; N 18.59. Found: C 49.95; H 3.87; N 18.35. HRMS *m*/*z* calculated for C_22_H_21_N_7_O_9_ [M+Na]+: 528.1400; found: 528.1466.

#### 2.1.18. 3,3′-((3-Hydroxyphenyl)azanediyl)bis(*N*′-(thiophen-3-yl)methylene)benzylidene)propanehydrazide) (**18**)

Violet powder, yield 0.21 g (30%), m.p. 116–118 °C (from propan-2-ol). ^1^H NMR (400 MHz, DMSO-d_6_) δ: 2.41–2.52 (m, overlaps with DMSO-d_6_, 2H, CH_2_CO), 2.77–2.92 (m, 2H, CH_2_CO), 3.45–3.71 (m, 4H, 2× NCH_2_), 6.00–8.22 (m, 12H, H_Ar_, H_Het,_ 2× CH), 9.04 (s, 1H, OH), 11.25, 11.30 (2s, 2H, 2× NH); ^13^C NMR (101 MHz, DMSO-d_6_) δ: 30.29, 30.45, 32.35, 32.54 (CH_2_CO), 46.36, 46.63 (NCH_2_), 98.83, 98.99, 103.03, 103.18, 103.36, 103.43, 124.58, 124.63, 124.68, 127.35, 127.40, 127.44, 127.54, 127.93, 137.33, 137.47, 137.87, 141.85, 148.39, 148.50, 158.50, 158.54, 167.08 (C_Ar_), 172.74 (C=O). IR (KBr): ν_max_ (cm^−1^) = 3198 (OH), 3090, 2967 (NH), 1667 (C=O). Anal. Calcd. for C_22_H_23_N_5_O_3_S_2_, %: C 56.27; H 4.94; N 14.91. Found: C 56.03; H 4.71; N 14.74.

#### 2.1.19. General Procedure for Preparation of Hydrazones **19**–**22**

A mixture of hydrazide 4 (1.8 mmol, 0.5 g) and the corresponding ketone (acetone, 2-butanone, acetophenone, or 4-acetylbenzenesulfonamide) (2.75 mmol) was heated under reflux for 5 h (methanol was used as a solvent (35 mL) in the synthesis of compound **22**). After the completion of the reaction, the mixture was cooled down, diluted with water (35 mL), and left in the refrigerator for 24 h. Then, the formed precipitate was filtered off, washed with diethyl ether, and recrystallized from 1,4-dioxane to give title compounds **19**–**22**. Product 20 was separated from the reaction mixture by evaporating the volatile fractions under reduced pressure, diluting the residue with diethyl ether, filtering the obtained solid, and recrystallizing from 1,4-dioxane to give title compound **20**.

#### 2.1.20. 3,3′-((3-Hydroxyphenyl)azanediyl)bis(*N*′-(propan-2-ylidene)propanehydrazide) (**19**)

Light brown powder, yield 0.36 g (55%), m.p. 169–171 °C (from propan-2-ol). ^1^H NMR (400 MHz, DMSO-d_6_) δ: 1.83, 1.91 (2s, 12H, 4× CH_3_), 2.50 (overlaps with DMSO-d_6_) and 2.65–2.77 (m, 4H, CH_2_CO), 3.42–3.59 (m, 4H, 2× NCH_2_), 5.98–6.28 (m, 3H, H_Ar_), 6.88–6.97 (m, 1H, H_Ar_), 8.97, 8.99, 9.02 (3s, 1H, OH), 10.00, 10.06 (2s, 2H, 2× NH); ^13^C NMR (101 MHz, DMSO-d_6_) δ: 17.05, 17.51, 24.96, 25.11 (CH_3_), 30.70, 30.78, 31.10, 31.98, 32.19 (CH_2_CO), 46.12, 46.21, 46.73, 46.81 (NCH_2_), 98.80, 98.94, 99.22, 103.11, 103.21, 103.44, 129.76, 148.46, 150.36, 150.39, 154.76, 154.83, 158.45, 167.03, 167.09 (C_Ar_), 172.90, 172.94 (C=O). IR (KBr): ν_max_ (cm^−1^) = 3256 (OH), 3046, 2999 (NH), 1670 (C=O). Anal. Calcd. for C_18_H_27_N_5_O_3_, %: C 59.81; H 7.53; N 19.38. Found: C 59.63; H 7.33; N 19.12.

#### 2.1.21. 3,3′-((3-Hydroxyphenyl)azanediyl)bis(*N*′-(butan-2-ylidene)propanehydrazide) (**20**)

Brown powder, yield 0.25 g (35%), m.p. 72–74 °C (from propan-2-ol). ^1^H NMR (400 MHz, DMSO-d_6_) δ: 0.88–1.09 and 1.69–1.92 (2m, 12H, 4× CH_3_), 2.21 (q, *J* = 7.5 Hz, 4H, 2× CH_2_CH_3_), 2.50 (overlaps with DMSO-d_6_) and 2.65–2.81 (m, 4H, 2× CH_2_CO), 3.40–3.61 (m, 4H, 2× NCH_2_), 5.98–6.26 (m, 3H, H_Ar_), 6.83–6.99 (m, 1H, H_Ar_), 8.97, 8.99, 9.02 (3s, 1H, OH), 9.97, 10.06, 10.16 (3s, 2H, 2× NH); ^13^C NMR (101 MHz, DMSO-d_6_) δ: 9.75, 10.61, 10.87, 15.69, 15.91 (CH_3_), 22.14, 22.41, 22.91, 23.33 (CH_2_CH_3_), 30.70, 30.88, 31.47, 31.54, 32.02, 32.16 (CH_2_CO), 46.22, 46.72 (NCH_2_), 98.75, 98.93, 99.20, 99.52, 103.04, 103.18, 103.26, 103.42, 129.72, 129.80, 148.45, 153.84, 153.93, 158.25, 158.45, 167.04, 167.13 (C_Ar_), 173.07, 173.12 (C=O). IR (KBr): ν_max_ (cm^−1^) = 3220 (OH), 3044, 2971 (NH), 1656 (C=O). Anal. Calcd. for C_20_H_31_N_5_O_3_, %: C 61.67; H 8.02; N 17.98. Found: C 61.41; H 7.87; N 17.74.

#### 2.1.22. 3-((3-Hydroxyphenyl)(3-oxo-3-(2-(1-phenylethylidene)hydrazineyl)propyl)amino)-*N*′-(1-phenylethylidene)propanehydrazide (**21**)

Light brown powder, yield 0.64 g (74%), m.p. 196–198 °C (from propan-2-ol). ^1^H NMR (400 MHz, DMSO-d_6_) δ: 2.16–2.28 (m, 6H, 2× CH_3_), 2.58–2.68 and 2.86–3.01 (2m, 4H, 2× CH_2_CO), 3.52–3.71 (m, 4H, 2× NCH_2_), 6.01–6.29 (m, 3H, H_Ar_), 6.82–7.02 (m, 1H, H_Ar_), 7.28–7.45 (m, 6H, H_Ar_), 7.65–7.80 (m, 4H, H_Ar_), 9.02, 9.04, 9.06 (3s, 1H, OH), 10.39, 10.41, 10.53, 10.55 (4s, 2H, 2× NH); ^13^C NMR (101 MHz, DMSO-d_6_) δ: 13.65, 13.70, 14.10 (CH_3_), 30.67, 30.79, 32.22, 32.29 (CH_2_CO), 46.42, 46.75, 46.80 (NCH_2_), 98.80, 99.02, 103.10, 103.30, 103.38, 103.57, 125.95, 126.00, 126.28, 128.25, 128.33, 128.36, 128.88, 129.11, 129.85, 138.17, 138.28, 147.54, 147.63, 148.45, 151.01, 151.11, 158.51, 167.75 (C_Ar_), 173.76, 173.82 (C=O). IR (KBr): ν_max_ (cm^−1^) = 3392 (OH), 3187, 3051 (NH), 1657 (C=O). Anal. Calcd. for C_28_H_31_N_5_O_3_, %: C 69.26; H 6.44; N 14.42. Found: C 69.00; H 6.27; N 14.23. HRMS *m*/*z* calculated for C_28_H_31_N_5_O_3_ [M+Na]^+^: 508.2426; found: 508.2318.

#### 2.1.23. 4,4′-(((3,3′-((3-Hydroxyphenyl)azanediyl)bis(propanoyl))bis(hydrazin-2-yl-1-ylidene))bis(ethan-1-yl-1-ylidene))dibenzenesulfonamide (**22**)

Light purple powder, yield 0.65 g (56%), m.p. 199–201 °C (from propan-2-ol). ^1^H NMR (400 MHz, DMSO-d_6_) δ: 2.14–2.33 (m, 6H, 2× CH_3_), 2.59–2.70 and 2.88–3.04 (2m, 4H, 2× CH_2_CO), 3.49–3.69 (m, 4H, 2× NCH_2_), 6.02–6.32 (m, 3H, H_Ar_), 6.83–7.02 (m, 1H, H_Ar_), 7.30–7.43 (m, 4× NH_2_), 7.71–8.00 (m, 8H, H_Ar_), 9.06, 9.07 (2s, 1H, OH), 10.52, 10.67, 10.69 (3s, 2H, 2× NH); ^13^C NMR (101 MHz, DMSO-d_6_) δ: 13.72, 14.11 (CH_3_), 30.57, 30.77, 32.21, 32.35 (CH_2_CO), 46.52, 46.62, 46.64, 46.77 (NCH_2_), 98.93, 99.09, 103.20, 103.32, 103.45, 109.63, 125.71, 125.98, 126.43, 126.73, 128.89, 129.89, 141.24, 141.30, 144.00, 144.22, 146.35, 148.43, 148.44, 149.49, 150.23, 150.86, 158.54, 165.48, 168.09, 168.17 (C_Ar_), 174.00, 174.08 (C=O). IR (KBr): ν_max_ (cm^−1^) = 3302 (OH), 3185, 3032 (NH), 1671 (C=O). Anal. Calcd. for C_28_H_33_N_7_O_7_S_2_, %: C 52.24; H 5.17; N 15.23. Found: C 52.03; H 4.87; N 15.07.

#### 2.1.24. 3,3′-((3-Hydroxyphenyl)azanediyl)bis(*N*-(2,5-dimethyl-1*H*-pyrrol-1-yl)propanamide) (**23**)

To a solution of dihydrazide 4 (0.3 g, 1.1 mmol) in propan-2-ol (25 mL), hexane-2,5-dione (0.5 g, 4.4 mmol) and a catalytic amount of acetic acid (0.1 mL) were added, and the mixture was heated under reflux for 5 h, then cooled down, and diluted with water (25 mL); the formed precipitate was filtered off, washed with water, and recrystallized from a mixture of propan-2-ol and water. Violet powder, yield 0.17 g (36%), decomposition at 148 °C. ^1^H NMR (400 MHz, DMSO-d_6_) δ: 1.95 (s, 12H, 4× CH_3_); 2.55 (t, *J* = 6.9 Hz, 4H, 2× CH_2_CO), 3.61 (t, *J* = 7.0 Hz, 4H, 2× NCH_2_), 5.62 (s, 4H, 4× CH_pyr_); 5.97–6.27 (m, 3H, H_Ar_), 6.86–7.02 (m, 1H, H_Ar_), 9.08 (s, 1H, OH), 10.65 (s, 2H, 2× NH); ^13^C NMR (101 MHz, DMSO-d_6_) δ: 10.93 (4× CH_3_), 31.30 (CH_2_CO), 46.52 (NCH_2_), 99.55, 102.90, 103.69, 103.85, 103.92, 126.66, 129.94, 148.20, 158.51 (C_Ar_), 170.24 (C=O). IR (KBr): ν_max_ (cm^−1^) = 3247 (OH), 2981, 2921 (2× NH), 1673 (2× C=O). Anal. Calcd. for C_24_H_31_N_5_O_3_, %: C 65.88; H 7.14; N 16.01. Found: C 65.62; H 6.91; N 15.83. HRMS *m*/*z* calculated for C_24_H_31_N_5_O_3_ [M+H]^+^: 438.2426; found: 438.2502.

#### 2.1.25. 3,3′-((3-Hydroxyphenyl)azanediyl)bis(1-(3,5-dimethyl-1*H*-pyrazol-1-yl)propan-1-one) (**24**)

To a solution of dihydrazide 5 (0.5 g, 1.8 mmol) in propan-2-ol (28 mL), pentane-2,4-dione (0.9 g, 9.0 mmol) and a catalytic amount of hydrochloric acid (0.05 mL) were added, and the mixture was heated under reflux for 5 h and then cooled down. The solvent was removed under reduced pressure, the residue was poured with water (30 mL), and the formed precipitate was filtered off, washed with water and diethyl ether, and recrystallized from a mixture of propan-2-ol and water. White powder, yield 0.35 g (62%), m.p. 158–160 °C (from propan-2-ol). ^1^H NMR (400 MHz, DMSO-d_6_) δ: 2.16 and 2.46 (2s, 12H, 4× CH_3_); 3.28 (t, *J* = 7.1 Hz, 4H, 2× CH_2_CO), 3.68 (t, *J* = 7.1 Hz, 4H, 2× NCH_2_), 6.02–6.30 (m, 5H, H_Ar_, 2× CH_Het_), 6.95 (t, *J* = 8.1 Hz, 1H, H_Ar_), 9.02 (s, 1H, OH); ^13^C NMR (101 MHz, DMSO-d_6_) δ: 13.42 14.07 (4× CH_3_), 33.27 (CH_2_CO), 46.13 (NCH_2_), 99.3,0 103.49, 103.66, 111.18, 129.88, 143.15, 148.15, 151.47, 158.47 (C_Ar_), 172.07 (C=O). IR (KBr): ν_max_ (cm^−1^) = 3275 (OH), 1721 (2× C=O). Anal. Calcd. for C_22_H_27_N_5_O_3_, %: C 64.53; H 6.65; N 17.10. Found: C 64.33; H 6.41; N 16.87. HRMS *m*/*z* calculated for C_22_H_27_N_5_O_3_ [M+H]^+^: 410.2113; found: 410.2187.

#### 2.1.26. 3,3′-((3-Hydroxyphenyl)azanediyl)bis(*N*′-(2-oxoindolin-3-ylidene)propanehydrazide (**25**)

To a solution of dihydrazide 4 (2.1 mmol) in propan-2-ol (22 mL), isatin (5.1 mmol) and glacial acetic acid (2 drops) were added. The reaction mixture was heated under reflux for 12 h and then cooled down. The formed precipitate was filtered off, washed with propan-2-ol, and recrystallized from 1,4-dioxane. Light brown powder, yield 0.72 g (64%), m.p. 207–208 °C, (from propan-2-ol). ^1^H NMR (400 MHz, DMSO-d_6_) δ: 2.71–3.11 (m, 4H, 2× CH_2_CO), 3.57–3.81 (m, 4H, 2× NCH_2_), 6.00–8.22 (m, 12H, H_Ar_), 9.05 (br. s, 1H, OH); 10.78, 11.13, 11.22, 12,55, 12.92 (5s, 4H, 2× NHC=N, 2× NHN). ^13^C NMR (101 MHz, DMSO-d_6_) δ: 29.73 (CH_2_CO), 46.21 (NCH_2_), 98.99, 103.81, 110.58, 111.06, 115.41, 121.78, 126.15, 130.17, 131.46, 132.56, 142.45, 143.80, 147.94, 158.50, 162.69 (C_arom_), 164.88, 173.70 (C=O); IR (KBr): ν_max_ (cm^−1^) = 3223 (OH), 3063, 2965 (NH), 1715, 1683 (C=O). Anal. Calcd. for C_28_H_25_N_7_O_5_, %: C 62.33; H 4.67; N 18.17. Found: C 62.15; H 4.73; N 18.05.

### 2.2. Microbial Strains and Culture Conditions

The multidrug-resistant *S. aureus* strain TCH 1516 [USA 300-HOU-MR] was obtained from the American Type Culture Collection (ATCC, Manassas, VA, USA) [36] (Table 1). *E. faecalis* AR-0671, multidrug-resistant *E. coli* AR-0001, *K. pneumoniae* AR-0003, *P. aeruginosa* AR-1114, and *A. baumannii* AR-0273 strains were acquired from the Centers for Disease Control and Prevention (CDC) AR isolate bank (Table 1). Prior to the initiation of this study, all microbial strains were stored in commercial cryopreservation systems at a temperature of −80 °C. The strains were cultivated on Columbia sheep blood agar (Becton Dickinson, Franklin Lakes, NJ, USA) or potato dextrose agar (PDA) for Candida (Becton Dickinson, Franklin Lakes, NJ, USA).

### 2.3. Minimal Inhibitory Concentration Determination

The antimicrobial activity of 3-((3-hydroxyphenyl)amino)propanoic acid derivatives was assessed by using the broth microdilution method, following the guidelines outlined by the Clinical Laboratory Standards Institute (CLSI), with modifications [36,37]. In brief, the compounds were dissolved in dimethyl sulfoxide (DMSO) to attain a final concentration of 25–30 mg/mL. Vancomycin hydrochloride (MedChem express, Cat. No. HY-17362), cefazolin (MedChem express, Cat. No. HY-B1892), and meropenem (MedChem express, Cat. No. HY-13678) were used as control antimicrobial drugs and were dissolved in DMSO as described before. Dilution series were prepared in deep 96-well microplates to achieve a two-fold concentration range of 0.25, 0.5, 1, 2, 4, 8, 16, 32, and 64 µg/mL, utilizing cation-adjusted Mueller–Hinton broth (CAMHB) as the growth medium. The microplates containing the dilution series were then inoculated with fresh cultures of each tested organism to reach a final concentration of 5 × 10^4^ CFU (colony-forming units) of the test organism in media containing 1% DMSO and 1× drug concentration, with a volume of 200 µL per well. Wells that were inoculated with medium containing 1% DMSO served as positive controls. Subsequently, the microplates were incubated at 35 ± 1 °C for 18 ± 2 h. Following the incubation period, the plates were examined by using a manual microplate viewer (Sensititre Manual Viewbox, ThermoFisher Scientific, Waltham, MA, USA). The minimal inhibitory concentration (MIC) was defined as the lowest concentration (µg/mL) of the tested drug that completely inhibited the growth of the test organism. All experiments were conducted in duplicate with three technical replicates for each condition.

### 2.4. Preparation of Test Compounds and Screening Libraries

Test compounds **2**–**25** were dissolved in hybridoma-grade dimethyl sulfoxide (Millipore, Sigma, Burlington, MA, USA) to prepare stock solutions at concentrations of 10–25 mg/mL. Cisplatin and doxorubicin hydrochloride (MedChemExpress, South Brunswick Township, NJ, USA) were dissolved in DMSO, and the dissolved compounds were then manually dispensed into deep 96-well plates, sealed, and stored at −80 °C until the day of the experiment. For in vitro anticancer activity screening, the compounds were thawed at room temperature, protected from light, and the aliquots were diluted in complete cell culture media to achieve a final concentration of 100 µM and used for the in vitro assays. The cytotoxicity assays were performed in triplicate.

### 2.5. Cell Lines and Culture Conditions

FaDu cells were obtained from the American Type Culture Collection (Rockville, MD, USA). HEK293 cells were kindly provided by Dr. Iliev lab at the Jill Roberts Institute for Inflammatory Bowel Disease, Weill Cornel Medicine of Cornel University (New York, NY, USA). All cells were cultivated in Dulbecco’s Modified Eagle Medium/Nutrient Mixture F-12 (DMEM/F-12) (Gibco, Waltham, MA, USA), 10% fetal bovine serum (FBS) (Gibco, Waltham, MA, USA), 100 U/mL penicillin, and 100 μg/mL streptomycin (P/S) (Gibco, Waltham, MA, USA). Culturing conditions were maintained at 37 °C in a humidified atmosphere containing 5% CO_2_. The culture medium was refreshed every 2–3 days, and cells were passaged upon reaching 70–80% confluence.

### 2.6. MTT-Based Cell Viability Assay

The in vitro inhibitory effects of the compounds were assessed by using the MTT assay [38]. Briefly, cells were plated in 96-well plates at a density of 1 × 10⁴ cells per well. After the cells were allowed to adhere overnight at 37 °C in 5% CO₂, they were treated with compounds at a concentration of 100 µM in triplicate. Following a 20 h incubation, the MTT reagent was added, and the cells were further incubated for 4 h. The resulting formazan was solubilized in anhydrous DMSO, and absorbance was measured at 570 nm by using a microplate reader. Cell viability was calculated by using the following formula: ([AE − AB]/[AC − AB]) × 100%, where AE, AC, and AB represent the absorbance values of experimental samples, untreated controls, and blank wells, respectively. Data analysis was performed by using GraphPad Prism or QuickCalcs.

### 2.7. IC_50_ Determination

The IC_50_ values, defined as the concentration of compound required to reduce cell viability by 50%, were determined by using a dose–response curve. The data were fitted to a nonlinear regression model (GraphPad Prism version 9.0, GraphPad Software, San Diego, CA, USA) to calculate the IC_50_ values for each compound tested in triplicate.

### 2.8. Superoxide Dismutase (SOD) Activity Determination

FaDu cells were seeded onto 6-well tissue culture plates at a density of 5 × 10^5^ cells per well and incubated overnight to allow for cell attachment. Following the incubation period, the cells were treated with the selected compounds dissolved in tissue culture medium containing 0.25% DMSO and incubated for an additional 6 h. After the treatment, the medium was aspirated, and the cells were washed twice with phosphate-buffered saline (PBS) to remove residual compounds. The cells were then trypsinized for 10 min to detach them from the culture surface. Following trypsinization, the cells were collected by centrifugation, washed with ice-cold PBS to remove any residual trypsin, and resuspended in 0.5 mL of ice-cold PBS. The cell suspension was then subjected to sonication on ice for disruption. After sonication, the samples were centrifuged at 10,000× *g* for 10 min at 4 °C to remove cell debris. The resulting supernatant was carefully collected for the subsequent assessment of SOD activity. The activity of superoxide dismutase (SOD) was determined by using a commercial colorimetric assay kit (Superoxide Dismutase (SOD) Colorimetric Activity Kit, Catalog Number EIASODC) (Invitrogen, ThermoFisher Scientific, Waltham, MA, USA) following the manufacturer’s instructions.

### 2.9. Hydrogen Peroxide Production Assay

FaDu cells were seeded on the 6-well plates and incubated with the compounds as described before. After incubation, cells were cooled down on ice, washed two times with ice-cold PBS, and scraped in PBS. Cells were centrifuged at 10,000× *g* for 10 min at 4 °C, the supernatant was discarded, and 650 µL of ice-cold PBS was added. Cells were sonicated and centrifuged at 10,000× *g* for 10 min at 4 °C to remove cell debris. The resulting supernatant was carefully collected for subsequent hydrogen peroxide analysis by using a commercial kit (Pierce™ Quantitative Peroxide Assay Kit (Aqueous), Catalog number 23280) (Invitrogen, ThermoFisher Scientific, Waltham, MA, USA).

### 2.10. In Silico Molecular Modeling

#### 2.10.1. Receptor Preparation

The crystal structures of 13 selected proteins (Table 2) were retrieved from the Protein Data Bank [39]. These proteins, such as cyclooxygenase-2, mitogen-activated protein kinases, tyrosine protein kinase receptor, mesenchymal–epithelial transition receptor, epidermal growth factor receptors, vascular endothelial growth factor receptor, and estrogen receptors, among others, are overexpressed in head and neck squamous cell carcinoma [40,41,42,43,44,45,46,47,48,49].

#### 2.10.2. Ligand Preparation

The 3D structure of candidate compounds **5**, **6**, **16**, and **25** were built by using GaussView 5.0 and geometrically optimized by using Avogadro (version 2.0) [48]. These structures were visually checked to correct some structural errors. The 3D structure of the ligands was extracted from crystal 7JXH and 3RHK, whereas the structure of an inhibitor drug (erlotinib) was taken from the PubChem database.

#### 2.10.3. Docking of Ligand–Protein Interaction

The compounds were docked into proteins to identify their potential binding site. Both ligand and protein were prepared by using AutoDock Tools, version 1.5.7. Docking calculations were performed by using AutoDock Vina [50,51]. Gasteiger partial charges were assigned to the atoms of the ligand. The AutoTors option was used to define the rotatable bonds in the ligand. We selected a grid size enough to cover each receptor. Finally, graphical analysis was performed by using VMD [52] and Discovery Studio (Dassault Systèmes BIOVIA. Discovery Studio Visualizer; V20.1.0, Vol19295; Dassault Systèmes: San Diego, CA, USA, 2021).

### 2.11. Statistical Analysis

The data are expressed as means ± SD from three independent experiments, unless otherwise stated. Statistical significance was determined by using a one-way ANOVA test in GraphPad Prism software. A *p* < 0.05 was considered statistically significant.

## 3. Results

### 3.1. Synthesis and Characterization of N-Aryl-Substituted β-Amino Acid Derivatives

**Scheme 1:**
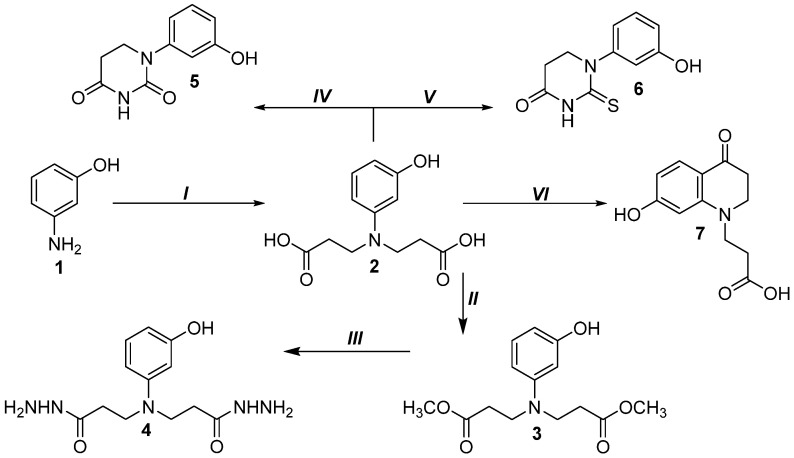
Synthesis of compounds **2**–**7**. (***I***) acrylic acid, H_2_O, 75 °C, 5 h; (***II***) CH_3_OH, H^+^, reflux, 7 h; (***III***) N_2_H_4_.H_2_O, *i*-PrOH, reflux, 6 h; (***IV***) carbamide, HCl, reflux, 1 h; (***V***) KSCN, HCl, reflux, 1 h; (***VI***) acrylic acid, hydroquinone, C_6_H_6_, reflux, 3 h.

The decision to synthesize compounds containing the *β*-alanine moiety has been stipulated by the possibility to synthesize new biologically active compounds [22,23]. Quite many *N*-aryl-substituted *β*-amino acids and their derivatives have been synthesized; however, the data on the synthesis and properties of 3-hydroxyphenyl-*β*-amino acid derivatives are limited. One of the convenient methods for the synthesis of *N*-aryl-*β*-alanines is the interaction of aromatic amines with acrylic acid. 3,3′-((3-Hydroxyphenyl)azanediyl)dipropionic acid (**2**) was synthesized by the addition reaction of 3-aminophenol (**1**) with acrylic acid in water (Scheme 1).

The reactions were carried out under reflux for 5 h. Dimethyl esters **3** were synthesized by the esterification of 3,3′-((3-hydroxyphenyl)azanediyl)dipropionic acid (**2**) with an excess of methanol in the presence of a catalytic amount of sulfuric acid. Subsequently, dihydrazide **4** was obtained by the hydrazinolysis of dimethyl esters **3** in propan-2-ol under reflux in 3 h. Comparing the ^1^H NMR spectrum of dihydrazide **4** with the ^1^H NMR spectrum of diester **3**, the characteristic signal of the ester groups was not observed at 3.60 ppm. A broad singlet integrated for four protons (4.20 ppm) and a singlet (9.04 ppm) which integrated for three protons and in which NH and OH groups overlap are attributed to the NH_2_, NH, and OH groups of the hydrazide molecule in the ^1^H NMR spectrum of compound **4** (Supplementary Materials, Figure S5).

Interaction between dicarboxylic acid **2** and carbamide or potassium thiocyanate gave corresponding compounds **5** and **6** with six-membered rings [25]. And, during the reaction between 3-aminophenol (**1**) and acrylic acid, quinoline derivative **7** was synthesized [26].

**Scheme 2:**
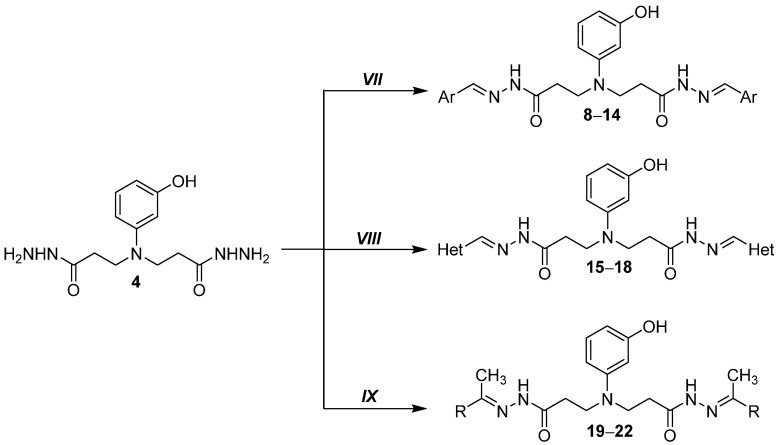
Synthesis of hydrazones **8**–**22**. (***VII***) ArCHO, *i*-PrOH, reflux, 2 h; (***VIII***) HetCHO, *i*-PrOH, reflux, 2 h; (***IX***) CH_3_COR, CH_3_OH (**22**), reflux, 5 h. **8**, Ar=C_6_H_5_; **9**, Ar=2,4-F_2_-C_6_H_3_; **10**, Ar=4-NO_2_-C_6_H_4_; **11**, Ar=4-Cl-C_6_H_4_; **12**, Ar=4-(CH_3_)_2_N-C_6_H_4_; **13**, Ar=3,4,5-(OCH_3_)_3_-C_6_H_2_; **14**, Ar=1-naphthyl; **15**, Het=2-furyl; **16**, Het=5-nitro-2-thienyl; **17**, Het=5-nitro-2-furyl; **18**, Het=3-thienyl; **19**, R=CH_3_; **20**, R=C_2_H_5_; **21**, R=C_6_H_5_; **22**, R=4-H_2_NO_2_SC_6_H_4_.

The condensation of compound **4** with aromatic aldehydes gave the corresponding hydrazones **8**–**18** (Scheme 2). The difference among compounds **8**–**18** is due to the mode of substitution in the azomethine fragment. The presence of particular substitution patterns in the benzene ring, as well as a mono substituent of azomethine fragment, caused the formation of geometrical isomers. NMR did not provide conclusive information about the separate conformations but gave a time-averaged spectral view of the structures present in the solution. Under analogous conditions, hydrazones **15**–**18** containing heterocyclic fragments in their structure were obtained. A detailed analysis of the NMR spectral data of **8**–**18** revealed the formation of rotamers. In the ^1^H NMR spectra, fragments of N=CH and CH_2_CO were observed as a double set of resonances with a descending value of chemical shift difference. Such chemical shift differences indicated the existence of the isomerism center. CH_2_CO fragments of compounds **12** were observed as two multiplets at 2.40–2.50 and 2.79–2.91 ppm (Supplementary Materials, Figure S15). Two resonances were attributed to the NH group in compounds **8**–**18**. The protons of the N=CH group were observed as two doubled lines in the ^1^H NMR spectra of all compounds **8**–**18**. The intensity ratio of the signals was 0.7:0.3 in each case, and it was determined by the same process, i.e., the hindered rotation of the amide fragment.

We compare the biological properties of the product ketones (acetone, ethyl methyl ketone, acetophenone, and 4-acetylbenzenesulfonamide) that were used in analog reactions, which gave derivatives **19**–**22**. The NMR spectra of compounds **19**–**22** were complicated due to the presence of an amide fragment in both side chains and the magnetic non-equivalence of each of the methyl groups in the azomethine fragment. The amide fragment in compounds **19**–**22** (Scheme 2) caused the formation of rotamers because of the restricted rotation around the CO-NH bond and was able to take part in the intermolecular and intramolecular interactions. Consequently, the formation of some stable structures was observed in the NMR spectra. For this reason, a double set of resonances was observed in the ^13^C NMR spectra for all carbon atoms.

**Scheme 3:**
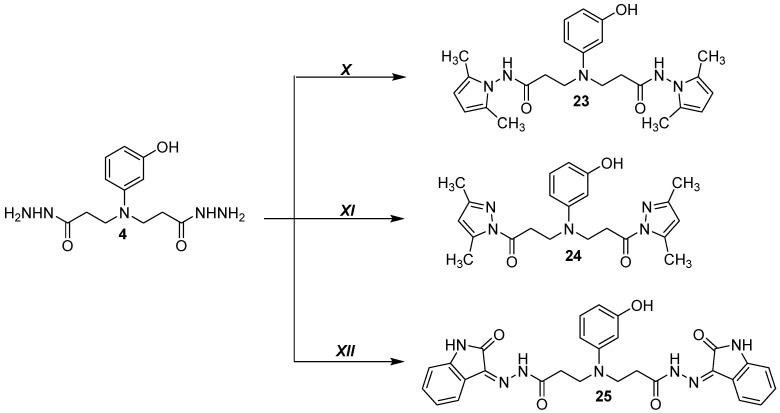
Synthesis of compounds **23**–**25**. (***X***) hexan-2,5-dione, CH_3_COOH, *i*-PrOH, reflux, 5 h; (***XI***) pentane-2,4-dione, HCl, *i*-PrOH, reflux, 5 h; (***XII***) isatin, CH_3_COOH, *i*-PrOH, reflux, 12 h.

The condensation of dihydrazide **4** with hexane-2,5-dione in the presence of a catalytic amount of acetic acid gave pyrrole compounds **23**. The reactions with pentane-2,4-dione in propan-2-ol in the presence of a catalytic amount of hydrochloric acid provided pyrazoles **24**. These reactions were carried out under reflux for 5 h (Scheme 3). The double intensity resonances at 10.93 ppm (CH_3_), 102.90 ppm (CH-CH), and 126.66 ppm (N-CCH_3_) in the ^13^C NMR spectrum of **23** (Supplementary Materials, Figure S38). proved the existence of a pyrrole ring. In the last stage of work, compound 25 with a 2-oxindole moiety was prepared by the reaction of *β*-amino acid dihydrazide **4** with isatin.

### 3.2. 3,3′-((3-Hydroxyphenyl)azanediyl)dipropionic Acid Derivatives ***2***–***25*** Show Antimicrobial Activity Against Multidrug-Resistant Bacterial Pathogens

After synthesizing and confirming the chemical structures of compounds **2**–**25**, we aimed to evaluate their in vitro antimicrobial activity by using a broth microdilution assay against multidrug-resistant pathogens with genetically defined resistance determinants, as well as clinically significant pathogens (Table 1).

The starting compound, 3,3′-((3-hydroxyphenyl)azanediyl)dipropionic acid (**2**) exhibited no antimicrobial activity against any of the tested bacterial strains (MIC > 64 μg/mL). Further chemical modifications yielded dimethyl ester **3**, which displayed weak activity against *Escherichia coli* AR-0001 (MIC of 32 μg/mL) but lacked activity against other Gram-negative and Gram-positive isolates. Dihydrazide **4** demonstrated promising activity against *Staphylococcus aureus* TCH-1516 (MIC of 8 μg/mL); however, no activity was observed against *Enterococcus faecalis* AR-0671 (MIC > 64 μg/mL) or Gram-negative isolates (MIC > 64 μg/mL). Six-membered-ring-containing compounds **5** and **6** exhibited activity against *S. aureus* TCH-1516 (MICs of 16 and 8 μg/mL, respectively) and *E. faecalis* AR-0671 (MIC of 32 μg/mL). Conversely, quinoline derivative **7** displayed no antimicrobial activity against any tested isolates (MIC > 64 μg/mL) (Table 1).

Next, we examined the antimicrobial activity of compounds containing aromatic substituents (**8**–**18**) to assess the role of specific functional groups. Compound **8**, bearing a phenyl substituent, exhibited no antimicrobial activity (MIC > 64 μg/mL). The introduction of a 2,4-difluorophenyl moiety (compound **9**) did not confer antimicrobial activity. However, the incorporation of a 4-nitrophenyl group (compound **10**) significantly enhanced activity against *S. aureus* TCH-1516 (MIC of 4 μg/mL) and *E. faecalis* AR-0671 (MIC of 16 μg/mL). Substituting the 4-nitrophenyl group with a 4-chlorophenyl moiety (compound **11**) reduced activity against *S. aureus* TCH-1516 (MIC of 64 μg/mL) and abolished activity against *E. faecalis* AR-0671 (MIC > 64 μg/mL). The 4-(dimethylamino)phenyl substituent (compound **12**) did not enhance activity against Gram-positive isolates; however, weak activity was observed against *Klebsiella pneumoniae* AR-0003, *Pseudomonas aeruginosa* AR-1114, and *Acinetobacter baumannii* AR-0273 (MIC of 64 μg/mL). The incorporation of a 3,4,5-trimethoxyphenyl moiety (compound **13**) resulted in the complete loss of antimicrobial activity (MIC > 64 μg/mL). Conversely, the introduction of a 1-naphthyl substituent (compound 14) substantially improved activity against *S. aureus* TCH-1516 (MIC of 2 μg/mL) and *E. faecalis* AR-0671 (MIC of 8 μg/mL) (Table 1).

To explore the impact of heterocyclic substituents on antimicrobial activity, we synthesized compounds **15**–**18**. Compound **15**, featuring a 2-furyl moiety, exhibited weak activity against *P. aeruginosa* AR-1114 and *A. baumannii* AR-0273 (MIC of 64 μg/mL) but was inactive against other strains. The incorporation of a 5-nitro-2-thienyl substituent (compound **16**) conferred strong activity against *S. aureus* TCH-1516 (MIC of 1 μg/mL) and *E. faecalis* AR-0671 (MIC of 4 μg/mL), as well as moderate activity against *E. coli* AR-0001 (MIC of 32 μg/mL), but lacked activity against other Gram-negative isolates. The introduction of a 5-nitro-2-furyl moiety (compound **17**) diminished activity against *S. aureus* TCH-1516 (MIC of 16 μg/mL) and *E. faecalis* AR-0671 (MIC > 64 μg/mL). The replacement of this moiety with a 3-thienyl substituent (compound **18**) completely abolished antimicrobial activity against all tested isolates (MIC > 64 μg/mL) (Table 1).

Compounds synthesized by using various ketones (**19**–**22**) exhibited structure-dependent activity. Compound **19**, bearing a methyl substituent, displayed no antimicrobial activity (MIC > 64 μg/mL). The introduction of an ethyl substituent (compound **20**) conferred activity against *S. aureus* TCH-1516 (MIC of 16 μg/mL) and *E. faecalis* AR-0671 (MIC of 32 μg/mL). The incorporation of a phenyl moiety (compound **21**) reduced activity against *S. aureus* TCH-1516 (MIC of 32 μg/mL). Notably, the addition of a 4-sulfamoylphenyl substituent (compound **22**) conferred activity against *S. aureus* TCH-1516 (MIC of 8 μg/mL), *E. faecalis* AR-0671 (MIC of 32 μg/mL), and *E. coli* AR-0001 (MIC of 16 μg/mL) (Table 1).

Pyrrole-containing compound **23** exhibited selective activity against *S. aureus* TCH-1516 (MIC of 64 μg/mL), whereas the introduction of a pyrazole substituent (compound **24**) resulted in the complete loss of antimicrobial activity. Finally, the isatin-containing compound **25** demonstrated activity against *S. aureus* TCH-1516 (MIC of 32 μg/mL), *E. faecalis* AR-0671 (MIC of 16 μg/mL), and *E. coli* AR-0001 (MIC of 64 μg/mL) (Table 1).

Collectively, these results demonstrate that 3,3′-((3-hydroxyphenyl)azanediyl)dipropionic acid derivatives shows structure-dependent antimicrobial activity against multidrug-resistant bacterial isolates harboring emerging resistance mechanisms.

### 3.3. 3,3′-((3-Hydroxyphenyl)azanediyl)dipropionic Acid Derivatives ***2***–***25*** Demonstrate Structure-Dependent Anticancer Activity

**Figure 2:**
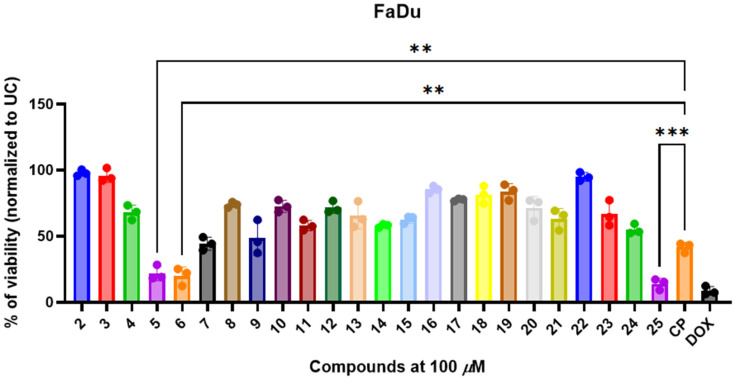
The 3,3′-((3-hydroxyphenyl)azanediyl)dipropionic acid derivatives (**2**–**25**) demonstrate structure-dependent anticancer activity in FaDu cells. The cells were exposed to the compounds, cisplatin (CP), or doxorubicin (DOX) for 24 h, and viability was measured by using the MTT assay. Data are presented as means ± SD from three experimental replicates. Statistical significance was determined by using a one-way ANOVA test. **—*p* < 0.01; ***—*p* < 0.001.

After demonstrating the in vitro antimicrobial activity of compounds **2**–**25** against multidrug-resistant bacterial isolates, we postulated that these N-aryl-substituted unnatural β-amino acids could harbor anticancer activity. To verify this, we focused on FaDu head and neck cancer models due to their immense resistance to radiation, cisplatin, and other FDA-approved anticancer medications. We exposed FaDu cells to a fixed 100 µM concentration of the compounds or cisplatin and doxorubicin, which served as control agents, and evaluated post-treatment viability by using an MTT assay (Figure 2).

Among the tested compounds, compound **6**, containing a thiocarbamide moiety, and compound **25**, bearing a 2-oxindole core, exhibited the most promising reduction in cell viability, suggesting that these substituents enhance anticancer activity. The high cytotoxicity observed for compound **6** is potentially due to the electron-withdrawing thiocyanate functionality, which may increase interaction with cellular targets. Similarly, the presence of the 2-oxindole scaffold in compound **25**, a pharmacophore frequently associated with anticancer properties, demonstrated significant activity in FaDu cells (Figure 2).

In contrast, compounds **2** and **3**, bearing the *β*-amino acid backbone with carboxyethyl and ester functional groups, respectively, exhibited minimal cytotoxicity, suggesting that these groups alone do not confer significant anticancer potential in FaDu cells. The introduction of the hydrazide functionality in compound **4** resulted in a moderate decrease in viability, indicating that the NH-NH₂ moiety contributes to enhanced activity. Furthermore, the tested hydrazones (compounds **8**–**18**) demonstrated variable activity, with the nature and position of the substituent on the benzene ring influencing their anticancer activity. Notably, compounds **12** and **15**, containing electron-withdrawing nitro and heterocyclic groups, respectively, displayed enhanced cytotoxic effects, supporting the hypothesis that these functionalities promote interaction with cellular targets (Figure 2).

Among the ketone-derived analogs (compounds **19**–**22**), cytotoxicity was moderate, with compound **19** (acetone-derived) displaying lower activity compared with compound **22**, which incorporates a 4-acetylbenzenesulfonamide moiety. This observation suggests that the presence of a sulfonamide group may enhance interactions with biological targets or mediates solubility and accessibility. Additionally, pyrrole (compound **23**) and pyrazole (compound **24**) derivatives exhibited reduced viability, further indicating that nitrogen-containing heterocycles contribute to cytotoxicity (Figure 2).

After characterizing the cytotoxic activity of compounds **2**–**25** in FaDu cells, we selected the most promising compounds (**5**, **6**, and **25**) for further analysis. We then aimed to determine whether the observed cytotoxicity in FaDu cells was selective for cancerous cells.

**Figure 3:**
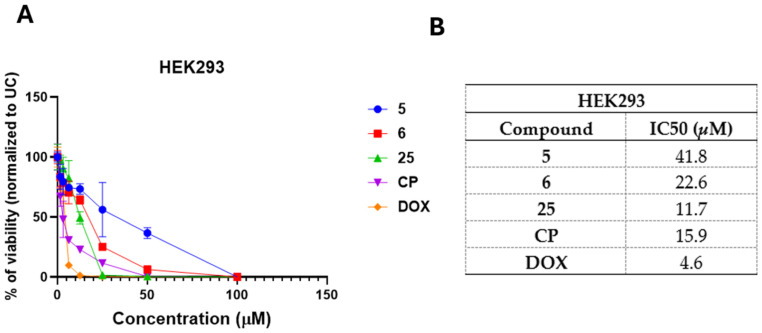
The selected 3,3′-((3-hydroxyphenyl)azanediyl)dipropionic acid derivatives (**5**, **6**, and **25**) exhibit favorable cytotoxic effects in non-cancerous HEK293T cells. (**A**) shows dose–response kinetics of compounds **5**, **6**, **25**, and control pharmaceuticals in HEK293 cells, while (**B**) demonstrates the calculated IC_50_ values. Cells were treated with the compounds, cisplatin (CP), or doxorubicin (DOX) for 24 h, and viability was assessed by using the MTT assay. Data are presented as means ± SD from three independent experiments.

The exposure of HEK293 cells to compounds reduced HEK293 viability in a dose-dependent manner (Figure 3A). Compounds **5** (IC_50_ = 41.8 µM) and **6** ((IC_50_ = 22.6 µM) showed significantly lower cytotoxicity (*p* < 0.05) in comparison with CP (IC_50_ = 15.9 µM) and DOX (IC_50_ = 4.6 µM), respectively. On the other hand, compound **25** showed stronger cytotoxic activity in HEK 293 cells, with IC_50_ = 11.7 µM, in comparison with compounds **5** and **6**, although compound **25** induced significantly lower cytotoxicity against Hek 293 cells than DOX (Figure 3B).

These results demonstrate that 3,3′-((3-hydroxyphenyl)azanediyl)dipropionic acid derivatives **2**–**25** harbor cytotoxic and structure-dependent cytotoxic activity in FaDu cells with multiple drug-resistance mechanisms, and the selected most promising anticancer compounds show favorable activity in non-cancerous HEK293 cells.

### 3.4. Selected and Most Promising 3,3′-((3-Hydroxyphenyl)azanediyl)dipropionic Acid Derivatives ***5***, ***6***, and ***25*** Are Able to Induce Cell Death and Oxidative Stress in FaDu Cells

**Figure 4:**
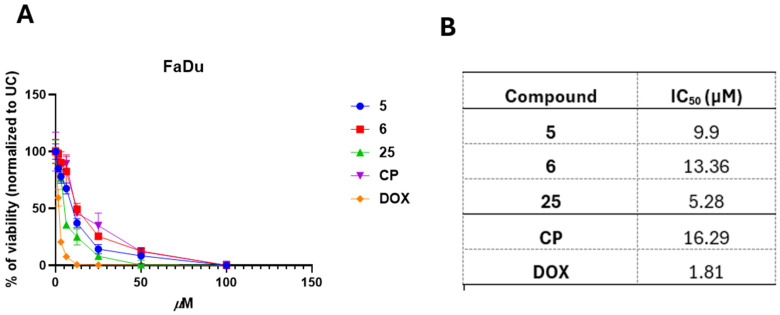
The most promising 3,3′-((3-hydroxyphenyl)azanediyl)dipropionic acid derivatives, **5**, **6**, and **25**, show dose-dependent anticancer activity in FaDu cells. (**A**) demonstrates the dose–response kinetics of these compounds, as well as cisplatin (CP) and doxorubicin (DOX), in FaDu cells. (**B**) shows the IC_50_ kinetics of these compounds in FaDu cells.

After selecting the most promising 3,3′-((3-hydroxyphenyl)azanediyl)dipropionic acid derivatives, **5**, **6**, and **25**, we aimed to establish the dose–response kinetics and compare them with standard chemoterapeutic drugs (CP and DOX). We exposed FaDu cells to the test compounds or control agents (0–100 µM) for 24 h and then measured their post-treatment viability and determined the IC_50_ (Figure 4A,B).

All selected compounds demonstrated low-micromolar, concentration-depended anticancer activity in FaDu cells (9.90–13.36 µM) (Figure 4A,B). Compounds **5**, **6**, and **25** exhibited higher anticancer activity in FaDu cells than CP (16.29 µM) (Figure 4).

**Figure 5:**
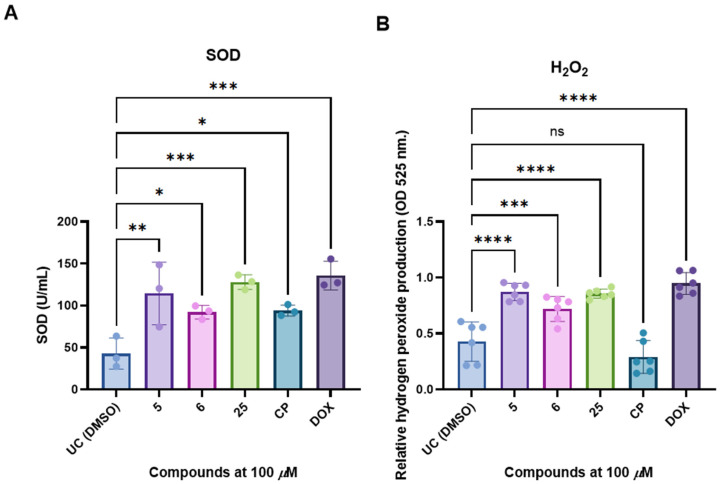
Compounds **5**, **6**, and **25** induce oxidative stress in FaDu cells. (**A**) demonstrates treatment-induced SOD activity, expressed as U/mL of cell lysate, while (**B**) shows relative hydrogen peroxide formation, expressed as optical density at 525 nm. The FaDu cells were exposed to a fixed concentration of compounds and test drugs (cisplatin (CP) and doxorubicin (DOX)) for 6 h in medium containing 0.25% DMSO, after which SOD activity and hydrogen peroxide levels were quantified by using commercial kits. The data are expressed as means ± SD from three experimental replicates for SOD activity or mean ± SD from six experimental replicates for hydrogen peroxide quantification. Statistical significance: * *p* < 0.05, ** *p* < 0.01, *** *p* < 0.001, and **** *p* < 0.0001; ns—not significant.

After establishing the dose-dependent and low-micromolar anticancer activity of the compounds in FaDu cells, we aimed to determine whether treatment with these compounds induces oxidative stress, as assessed by hydrogen peroxide formation and SOD activity. To investigate this, FaDu cells were treated with compounds **5**, **6**, and **25** or the cytotoxic agents cisplatin (CP) and doxorubicin (DOX) for 6 h, followed by the quantification of hydrogen peroxide levels and SOD activity (Figure 5).

Treatment with compounds **5**, **6**, and **25** resulted in a significant increase in oxidative stress markers in FaDu cells, as indicated by elevated SOD activity and hydrogen peroxide production (Figure 4A,B). Specifically, SOD activity (U/mL) was significantly higher in cells treated with compounds **5**, **6**, and **25** compared with the untreated control (UC; DMSO) (*p* < 0.05 to *p* < 0.001) (Figure 5A). The highest SOD activity was observed in cells treated with isatin-containing compound **25**, comparable to that induced by cisplatin (CP) and doxorubicin (DOX). Similarly, hydrogen peroxide levels, measured as optical density at 525 nm, were significantly higher following treatment with compounds **5** and **6** (*p* < 0.0001 and *p* = 0.0007) and compound **25** (*p* < 0.001) compared with the untreated control (Figure 5B). Notably, CP treatment resulted in a lower level of hydrogen peroxide production than the tested compounds in FaDu cells, potentially due to their resistance to cisplatin, whereas DOX induced a significant increase, demonstrating treatment-induced stress.

These data demonstrate that compounds **5**, **6**, and **25** promote oxidative stress in drug-resistant FaDu cells by enhancing both SOD activity and hydrogen peroxide accumulation, leading to potential anticancer effects.

### 3.5. The Molecular Modeling of the Most Potent Compound (***25***) with Major Overexpressed Head and Neck Cancer Targets

**Figure 6:**
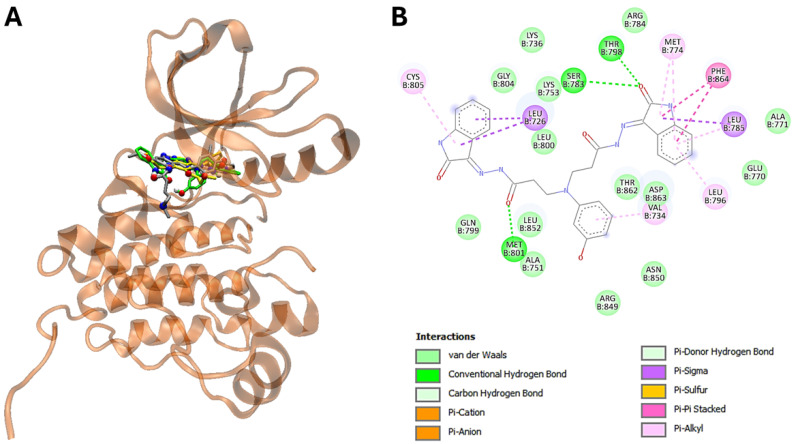
Compound **25** is proposed to interact with human epidermal growth factor receptor 2 (HER2) based on in silico molecular modeling. (**A**) visualizes the potential binding site of compound **25** (green), the ligand (silver), and erlotinib (yellow) within HER2. (**B**) presents 2D interaction maps illustrating hydrogen bonds (H-bonds) and hydrophobic interactions between compound **25** and HER2 residues. Van der Waals and π interactions are categorized as hydrophobic interactions.

After identifying the most promising *N*-aryl-substituted *β*-amino acid derivatives with low-micromolar activity on FaDu cells, we performed in silico molecular docking studies to identify possible biological targets for the cytotoxic compounds (**5**, **6**, **16**, and **25**) in order to obtain some information on their possible mechanisms of action (Table 2). For this purpose, we predicted the potential docking sites of the compounds in several cancer-related proteins and calculated their corresponding binding energies (∆G_bin_). To obtain high-reliability results, we reduced the search space to a set of cancer-related proteins of known 3D structures by establishing independent searches with the set of compounds, and we used their most stable conformers interacting with the biological targets. Table 2 shows the results of such a screening, which globally indicate that most cytotoxic compounds bind more strongly to epidermal growth factor receptor 2 (HER2) (Figure 6), with ∆G_bin_ values ranging from −11,7 to −7,8 (average −9,24) Kcal/mol, and mesenchymal–epithelial transition factor (c-MET), with values ranging from −11,6 to −7,4 (average −8,76) Kcal/mol (Table 2 and Table 3). Compound **25** is proposed to interact with HER2 via interactions through HER2-conserved Thr798 and Met801 amino acid residues (Table 3).

**Figure 7:**
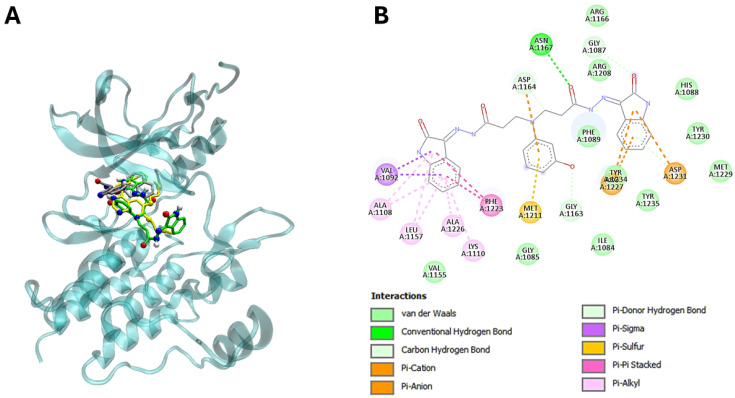
Compound **25** is proposed to interact with human mesenchymal–epithelial transition factor (c-Met) based on in silico molecular modeling. (**A**) visualizes the potential binding site of compound **25** (green), the ligand (silver), and erlotinib (yellow) within c-Met. (**B**) presents 2D interaction maps illustrating hydrogen bonds (H-bonds) and hydrophobic interactions between compound **25** and c-Met residues. Van der Waals and π interactions are categorized as hydrophobic interactions.

Interestingly, the energetic aspects of the interactions resulted favorable to **25** in comparison to erlotinib with favorable energy differences of 3,6 and 2,6 kcal/mol for EGFR and c-MET, respectively (Figure 7). On the other hand, the interactions did not result favorable for **25** in comparison with the ligand, with energy differences of −2,9 and −3,0 kcal/mol for EGFR and c-MET, respectively (Table 2 and Table 3).

Importantly, the 3-hydroxyphenyl core of these compounds plays a pivotal role in these interactions, directly contributing to the overlap with the ligands at the catalytic sites of the enzymes (Table 3; Figure 6 and Figure 7B).

Our molecular docking studies suggest that promising selected compounds in head and neck cancer cells (HNSCC cells) could interact with human epidermal growth factor receptor 2 (HER2) and mesenchymal–epithelial transition factor (c-MET). The elevated HER2 overexpression has been associated with a worse prognosis, increased recurrence, and decreased overall survival in HNSCC. Furthermore, HER2 has been found to be co-expressed with EGFR in HNSCC tumors, where EGFR is a protein that helps cells grow, and mutations in the EGFR gene can cause cancer cells to grow too quickly. On the other hand, c-MET is a receptor tyrosine kinase that is often dysregulated in cancers, including HNSCC, and its deregulation contributes to tumor progression, metastasis, and resistance to therapy [46,47,48]. Therefore, HER2 and c-MET are targets for cancer treatments, including targeted therapies and drugs that can also block other proteins, such as EGFR.

## 4. Discussion

3,3′-((3-Hydroxyphenyl)azanediyl)dipropionic acid and its derivatives, including diester, dihydrazide, dihydropyrimidines, and quinolinone, were synthesized and characterized. The reactions of *N*-carboxyethyl-*N*-(3-hydroxyphenyl)-*β*-alanine dihydrazide with mono- and dicarbonyl compounds were investigated. It was found that the reaction of the acid dihydrazide with aromatic and heterocyclic aldehydes and ketones resulted in the formation of hydrazone-type compounds, which existed as an E/Z isomeric mixture in DMSO-d_6_ solutions due to restricted rotation around the CO-NH bond. In contrast, reactions of the dihydrazide with dicarbonyl compounds, such as pentane-2,4-dione and hexane-2,5-dione, led to the formation of cyclic pyrazole and pyrrole derivatives.

The synthesized compounds exhibited promising structure–activity-dependent antimicrobial activity, primarily targeting Gram-positive pathogens with emerging resistance mechanisms. Among these, compounds **10**, **14**, and **16** demonstrated the most potent activity against methicillin-resistant *Staphylococcus aureus* and *Enterococcus faecalis*. The potent antimicrobial activity exhibited by compounds bearing 4-nitrophenyl (compound **10**), 1-naphthyl (compound **14**), and 5-nitro-2-thienyl (compound **16**) substituents against Gram-positive pathogens can be rationalized by their distinct electronic and physicochemical properties. The 4-nitrophenyl moiety possesses a strongly electron-withdrawing nitro group (Hammett σ_p = +0.78), which significantly decreases electron density on the aromatic ring through both inductive and resonance effects. This enhanced electrophilicity is hypothesized to facilitate covalent or non-covalent interactions with nucleophilic functional groups within bacterial proteins, impairing essential biological processes. The 1-naphthyl substituent, with its extended π-conjugated system, increases overall hydrophobicity (logP increment) and enables favorable π–π stacking interactions with hydrophobic domains of bacterial membranes or target macromolecules, promoting enhanced membrane penetration and target affinity. In compound **16**, the 5-nitro-2-thienyl group introduces both an electron-withdrawing nitro functionality and a sulfur-containing heterocyclic system, resulting in the modulation of the molecule’s dipole moment and further increasing its polarity and ability to traverse bacterial cell targets. Additionally, the electron deficiency of the thiophene ring, compounded by the nitro substitution, may favor specific binding interactions with bacterial enzymes targeted by these compounds. Despite the promising in vitro antimicrobial activity observed against Gram-positive bacterial pathogens, the current study is limited by the absence of data on additional clinically relevant *Staphylococcus* species and, notably, *Enterococcus faecium*—a high-priority multidrug-resistant pathogen often harboring vancomycin resistance and other emerging resistance mechanisms. Further investigations are warranted to determine whether the observed antimicrobial efficacy extends to these more resistant and epidemiologically significant strains. Furthermore, 3-((3-hydroxyphenyl)amino)propanoic acid derivatives demonstrated notable anticancer activity in drug-resistant FaDu head and neck squamous carcinoma cells. In the series, compounds **5**, **6**, and **25** were identified as the most promising candidates, exhibiting potent, dose-dependent cytotoxic effects with low micromolar IC₅₀ values. Importantly, these compounds also demonstrated low cytotoxic activity in non-cancerous HEK293 cells, suggesting cancerous cell-specific activity. Molecular docking analyses provided additional mechanistic insights, indicating that compound **25** engages in favorable binding interactions with two critical oncogenic receptors, HER2 (human epidermal growth factor receptor 2) and c-MET (hepatocyte growth factor receptor). Both HER2 and c-MET are well-established druggable targets implicated in tumor progression, metastasis, and therapeutic resistance across multiple cancer types. The predicted binding of compound **25** to these receptors supports the hypothesis that its anticancer activity may be mediated, at least in part, by the inhibition of growth factor signaling pathways crucial to cell proliferation and survival. These findings highlight the therapeutic potential of this scaffold as a platform for the development of novel anticancer agents targeting resistant head and neck cancers. While the synthesized 3,3′-((3-hydroxyphenyl)azanediyl)dipropionic acid derivatives demonstrated promising antimicrobial and anticancer activities, several limitations must be acknowledged. First, the biological evaluations were restricted to in vitro assays, and no in vivo validation of efficacy, pharmacokinetic behavior, metabolic stability, or toxicity profiles was performed, limiting the further translational relevance of the findings. Second, although molecular docking studies suggested favorable interactions between compound **25** and clinically relevant growth factor receptors such as HER2 and c-MET, these computational simulations were not corroborated by experimental binding affinity measurements, such as surface plasmon resonance (SPR), isothermal titration calorimetry (ITC), or cellular target validation assays. Additionally, although the E/Z isomerism of hydrazone derivatives was confirmed in DMSO-d6 by NMR, the potential impact of this isomeric mixture on biological activity, stability, and target binding affinity was not systematically investigated. Finally, the structure–activity relationship (SAR) analysis was preliminary, relying on limited chemical diversity within the tested library, and further iterative optimization based on rational design and target-based screening would be necessary to enhance potency, selectivity, and drug-like properties.

Overall, these results demonstrate that 3,3′-((3-hydroxyphenyl)azanediyl)dipropionic acid derivatives could serve as a novel scaffold for the development of compounds with antimicrobial and anticancer activity, particularly targeting drug-resistant pathogens and cancer cells. Furthermore, compound **25** could serve as a novel platform for the development of novel compound **25**-based derivatives targeting HER2 and c-MET growth factor receptors.

## 5. Conclusions

In this study, we successfully synthesized and characterized a series of novel *N*-aryl-substituted *β*-amino acid derivatives bearing a 3-hydroxy group and evaluated their in vitro antimicrobial and anticancer activity. These compounds demonstrated promising biological activity against multidrug-resistant bacterial pathogens and FaDu cancer cells, which are known for their resistance to chemotherapeutic agents. Structural modifications in the 3,3**′**-((3-hydroxyphenyl)azanediyl)dipropionic acid scaffold, particularly the introduction of electron-withdrawing groups and heterocyclic moieties, significantly enhanced their bioactivity, highlighting the importance of strategic chemical design in drug development.

Moreover, the synthesized compounds exhibited structure-dependent anticancer activity against the FaDu head and neck cancer cell line, which is characterized by resistance to platinum-based chemotherapy and radioresistance. The most potent derivatives displayed low micromolar inhibitory concentrations, comparable to doxorubicin, a standard chemotherapeutic agent. Molecular docking studies suggested that the most promising compound, **25**, bearing an isatin substituent, is capable of in silico binding to the c-MET and HER2 growth factor receptor proteins, which are key targets in anticancer drug development. Further studies are required to assess the in vivo efficacy, pharmacokinetic properties, and safety of these *N*-aryl-substituted *β*-amino acid derivatives, as well as to develop more potent analogs through hit-to-lead optimization strategies.

## Figures and Tables

**Table 1:** The in vitro antimicrobial activity expressed as minimal inhibitory concentration (MIC; μg/mL) of *N*-aryl-substituted *β*-amino acid derivatives **2**–**25** against multidrug-resistant bacterial pathogens with genetically defined resistance mechanisms.

Compound	*S. aureus* TCH 1516 ^1^	*E. faecalis* AR-0671 ^2^	*E. coli* AR-0001 ^3^	*K. pneumoniae* AR-0003 ^4^	*P. aeruginosa* AR-1114 ^5^	*A. baumannii* AR-0273 ^6^
**2**	>64	>64	>64	>64	>64	>64
**3**	>64	>64	32	>64	>64	>64
**4**	8	>64	>64	>64	>64	>64
**5**	16	32	>64	>64	>64	>64
**6**	16	32	>64	>64	>64	>64
**7**	>64	>64	>64	>64	>64	>64
**8**	>64	>64	>64	>64	>64	>64
**9**	>64	>64	>64	>64	>64	>64
**10**	4	16	>64	>64	>64	>64
**11**	64	>64	>64	>64	>64	>64
**12**	64	>64	>64	64	64	64
**13**	>64	>64	>64	>64	>64	>64
**14**	2	8	64	>64	>64	>64
**15**	>64	>64	>64	>64	64	64
**16**	1	4	32	>64	>64	>64
**17**	16	>64	>64	>64	>64	>64
**18**	>64	>64	>64	>64	>64	>64
**19**	>64	>64	>64	>64	>64	>64
**20**	16	32	>64	>64	>64	>64
**21**	32	32	>64	>64	>64	>64
**22**	8	32	16	>64	>64	>64
**23**	64	>64	>64	>64	>64	>64
**24**	>64	>64	>64	>64	>64	>64
**25**	32	16	64	>64	>64	>64
Vancomycin	2	4	N/A	N/A	N/A	N/A
Cefazolin	32	8	>64	>64	>64	>64
Meropenem	2	2	8	64	32	>64

^1^ *S. aureus* TCH 1516, *mecA* MRSA isolate, USA300 lineage. ^2^ *E. faecalis* AR-0671, *ant(6)-la*, *aph(3′)-III*, *erm(B)*, *sat-4A*, *tet(M)*. ^3^ *E. coli* AR-0001, *aac(6′)-Ib-cr*, *aadA5*, *ACRF*, *catB4*, *dfrA17*, *KPC-3*, *MDF(A)*, *mph(A)*, *OXA-1*, *sul1*, *tet(A)*, *tet(R)*. ^4^ *K. pneumoniae* AR-0003, *aac(6′)-Ib*, *aadA1*, *aph(3′)-Ib*, *aph(6)-Id*, *dfrA14*, *EMRD*, *KDEA*, *KPC-3*, *Omp35*, *OmpK35*, *oqxA*, *oqxB*, *OXA-9*, *SHV-12*, *sul2*, *TEM-1*. ^5^ *P. aeruginosa* AR-1114, *aac(6′)-Il*, *aacA4*, *aadA1*, *aadA6*, *bcr1*, *catB7*, *IMP-15*, *mexE*, *mexX*, *OXA-2*, *OXA-494*, *PDC-5*, *sul1*. ^6^ *A. baumannii* AR-0273, *aac(3)-IIe*, *ABAF*, *ADC-25*, *ADEC*, *aph(3′)-Ia*, *aph(6)-Id*, *aphA6*, *OXA-23*, *OXA-66*, *strA*, *sul2*, *tet(B)*. N/A—not applicable.

**Table 2:** Predicted binding free energy values (∆G_bin_ kcal/mol) of synthesized cytotoxic hybrids with selected proteins overexpressed in cancer.

Compound	Target Proteins												
	COX-2	FGFR-2	VEGRF-2	NR3A1	NR3A2	EGFR	HER2	c-MET	ERK2	MEK1	CK4	TPK	TopoII
**5**	−7,7	−7,0	−7,5	−7,2	−7,2	−7,9	−7,8	−7,4	−7,2	−7,0	−6,5	−7,5	−7,7
**6**	−7,3	−6,7	−7,1	−6,9	−6,9	−7,3	−7,8	−7,2	−7,2	−6,8	−6,4	−7,6	−7,3
**16**	−8,3	−8,0	−6,5	−8,3	−7,4	−8,4	−9,6	−8,9	−8,0	−8,8	−7,1	−8,1	−8,3
**25**	−9,8	−11,3	−7,6	−10,9	−10,8	−9,1	−11,7	−11,6	−10,3	−10,5	−10,2	−10,1	−9,8
P avge.	−8,29	−8,23	−7,19	−8,34	−8,09	−8,16	**−9,24**	**−8,76**	−8,16	−8,27	−7,54	−8,34	−7,54

Proteins with their respective (PDB) entries: COX-2: cyclooxygenase 2 (3LN1); FGFR-2: fibroblast growth factor receptor 2 (1GJO); VEGRF-2: vascular endothelial growth factor receptor 2 (3VHE); NR3A1: estrogen receptor α (3ERT); NR3A2: estrogen receptor beta (2QTU); EGFR: epidermal growth factor receptor (5GTY); HER2: epidermal growth factor receptor 2 (7JXH); c-MET: hepatocyte growth factor receptor (3RHK); ERK2: extra-cellular signal-regulated kinase 2 (2OJG); MEK1: MAPK/ERK kinase (4AN3); CK4: cyclin-dependent kinase 4 (1G3N); TPK: tyrosine-protein kinase (4EHZ); TopoII: topoisomerase II (5GWK). P avge.: protein average. The mean of the ∆Gbin values for the interactions of each protein with the compounds; the two proteins with the highest global compounds affinity are highlighted in red color.

**Table 3:** Binding site contacts of compound **25**, ligands, and drugs into HER2 and c-MET.

Compound	ΔG_bin_ (kcal/mol)	H Bonds and Hydrophobic Contacts in Binding Site
		HER2
**25**	−11,7	Leu726, Val734, Lys736, Ala751, Lys753, Glu770, Ala771, Met774, **Ser783**, Arg784, Leu785, Leu796, **Thr798**, Gln799, Leu800, **Met801**, Gly804, Arg849, Asn850, Leu852, Thr862, Asp863, Phe864
**Ligand 1 ^[a]^**	−14,6	Leu726, Val734, Ala751, Ile752, Lys753, Met774, **Ser783**, Leu785, Leu796, Thr798, Gln799, Leu800, Met801, Glu804, **Cys805**, Asp808, Leu852, Thr862, Asp863, Phe864
**Erlotinib ^[b]^**	−8,1	Leu726, Gly727, Ser728, Val734, Ala751, Lys753, Met774, Ser783, Leu785, Leu796, Thr798, Gln799, Leu800, Met801, Gly804, Cys805, Leu852, **Thr862**, Asp863, Phe864
		**c-MET**
**25**	−11,6	Ile1084, Gly1085, Gly1087, His1088, Phe1089, Val1092, Ala1108, Lys1110, Val1155, Leu1157, Gly1163, Asp1164, **Asn1167**, Arg1208, Met1211, Phe1223, Ala1226, Arg1227, Met1229, Tyr1230, Asp1231, Tyr1234, Tyr1235, Gly1163
**Ligand 2 ^[a]^**	−14,6	Ile1084, Gly1085, Phe1089, Val1092, Ala1108, Lys1110, Leu1140, Leu1157, **Pro1158**, Tyr1159, **Met1160**, Gly1163, Met1211, Phe1223, Ala1226, Arg1227
**Erlotinib ^[b]^**	−9,0	Ile1084, Phe1089, Val1092, Ala1108, Lys1110, Val1155, Leu1157, Met1160, Gly1163, Asp1164, Asn1167, Arg1208, Met1211, Phe1223, Ala1226, **Arg1227**, Asp1231, Tyr1234, Tyr1235

^[a]^ Ligands 1 and 2 correspond to (2*E*)-*N*-[3-cyano-7-ethoxy-4-({3-methyl-4-[([1,2,4]triazolo[1,5-a]pyridin-7-yl)oxy]phenyl}amino)quinolin-6-yl]-4-(dimethylamino)but-2-enamide and 1-[(3*R*,4*R*)-4-(1*H*-indol-3-yl)-2,5-dioxopyrrolidin-3-yl]pyrrolo[3,2,1-ij]quinolinium, respectively. The three-dimensional structure of ligands 1 and 2 was extracted from the Protein Data Bank by using PDB IDs 7JXH and 3RHK, respectively. ^[b]^ Drug compound that acts as inhibitor of biological targets. Bolded names correspond to amino acids involved in H bonds.

## Data Availability

All data generated during this study are provided in the manuscript and Supplementary Materials.

## Supplementary Materials

The following supporting information can be downloaded at: https://www.mdpi.com/article/10.3390/pathogens14050484/s1, Figure S1: ^1^H NMR spectrum of compound **2**, Figure S2: ^13^C NMR spectrum of compound **2**, Figure S3: ^1^H NMR spectrum of compound **3**, Figure S4: ^13^C NMR spectrum of compound **3**, Figure S5: ^1^H NMR spectrum of compound **4**, Figure S6: ^13^C NMR spectrum of compound **4**, Figure S7: ^1^H NMR spectrum of compound **8**, Figure S8: ^13^C NMR spectrum of compound **8**, Figure S9: ^1^H NMR spectrum of compound **9**, Figure S10: ^13^C NMR spectrum of compound **9**, Figure S11: ^1^H NMR spectrum of compound **10**, Figure S12: ^13^C NMR spectrum of compound **10**, Figure S13: ^1^H NMR spectrum of compound **11**, Figure S14: ^13^C NMR spectrum of compound **11**, Figure S15: ^1^H NMR spectrum of compound **12**, Figure S16: ^13^C NMR spectrum of compound **12**, Figure S17: ^1^H NMR spectrum of compound **13**, Figure S18: ^13^C NMR spectrum of compound **13**, Figure S19: ^1^H NMR spectrum of compound **14**, Figure S20: ^13^C NMR spectrum of compound **14**, Figure S21: ^1^H NMR spectrum of compound **15**, Figure S22: ^13^C NMR spectrum of compound **15**, Figure S23: ^1^H NMR spectrum of compound **16**, Figure S24: ^13^C NMR spectrum of compound **16**, Figure S25: ^1^H NMR spectrum of compound **17**, Figure S26: ^13^C NMR spectrum of compound **17**, Figure S27: ^1^H NMR spectrum of compound **18**, Figure S28: ^13^C NMR spectrum of compound **18**, Figure S29: ^1^H NMR spectrum of compound **19**, Figure S30: ^13^C NMR spectrum of compound **19**, Figure S31: ^1^H NMR spectrum of compound **20**, Figure S32: ^13^C NMR spectrum of compound **20**, Figure S33: ^1^H NMR spectrum of compound **21**, Figure S34: ^13^C NMR spectrum of compound **21**, Figure S35: ^1^H NMR spectrum of compound **22**, Figure S36: ^13^C NMR spectrum of compound **22**, Figure S37: ^1^H NMR spectrum of compound **23**, Figure S38: ^13^C NMR spectrum of compound **23**, Figure S39: ^1^H NMR spectrum of compound **24**, Figure S40: ^13^C NMR spectrum of compound **24**, Figure S41: ^1^H NMR spectrum of compound **25**, Figure S42: ^13^C NMR spectrum of compound **25**.
