# Supplementary material for: 3,3′-((3-Hydroxyphenyl)azanediyl)dipropionic Acid Derivatives as a Promising Scaffold Against Drug-Resistant Pathogens and Chemotherapy-Resistant Cancer
Source: Pathogens. 2025 May 15;14(5):484. doi: 10.3390/pathogens14050484 (PMC12115217; doi:10.3390/pathogens14050484)
Supplement: Supplementary file 1 [file pathogens-14-00484-s001.zip › pathogens-3602216-supplementary.pdf]

3,3'-((3-Hydroxyphenyl)azanediyl)dipropionic Acid Derivatives as a Promising Scaffold Against Multidrug-Resistant Pathogens and Chemotherapy-Resistant Cancer

Povilas Kavaliauskas [1,2,3,4], Waldo Acevedo [5], Eglė Mickevičiūtė [6], Ramunė Grigalevičiūtė [2,7], Birutė Grybaitė [1], Birutė Sapijanskaitė-Banevič [1], Guoda Pranaitytė [1] , Vidmantas Petraitis [8], Rūta Petraitienė [8], and Vytautas Mickevičius [1,*]

Supplementary information

^{1}H and ^{13}C NMR spectra of compounds 2–4, 8–25
(in DMSO-d_6)

3,3'-((3-Hydroxyphenyl)azanediyl)dipropionic acid ***(2)***

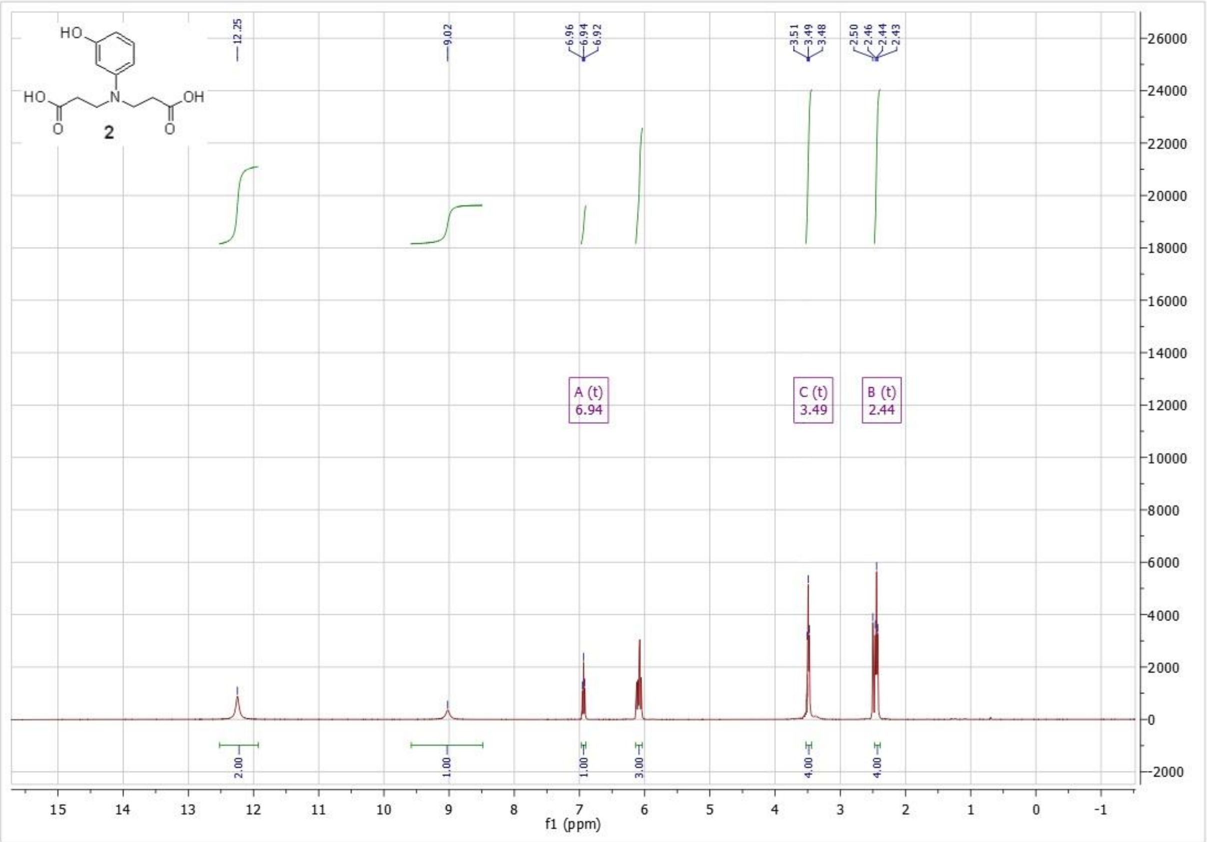

Figure S1. ^{1}H NMR spectrum of compound **2**

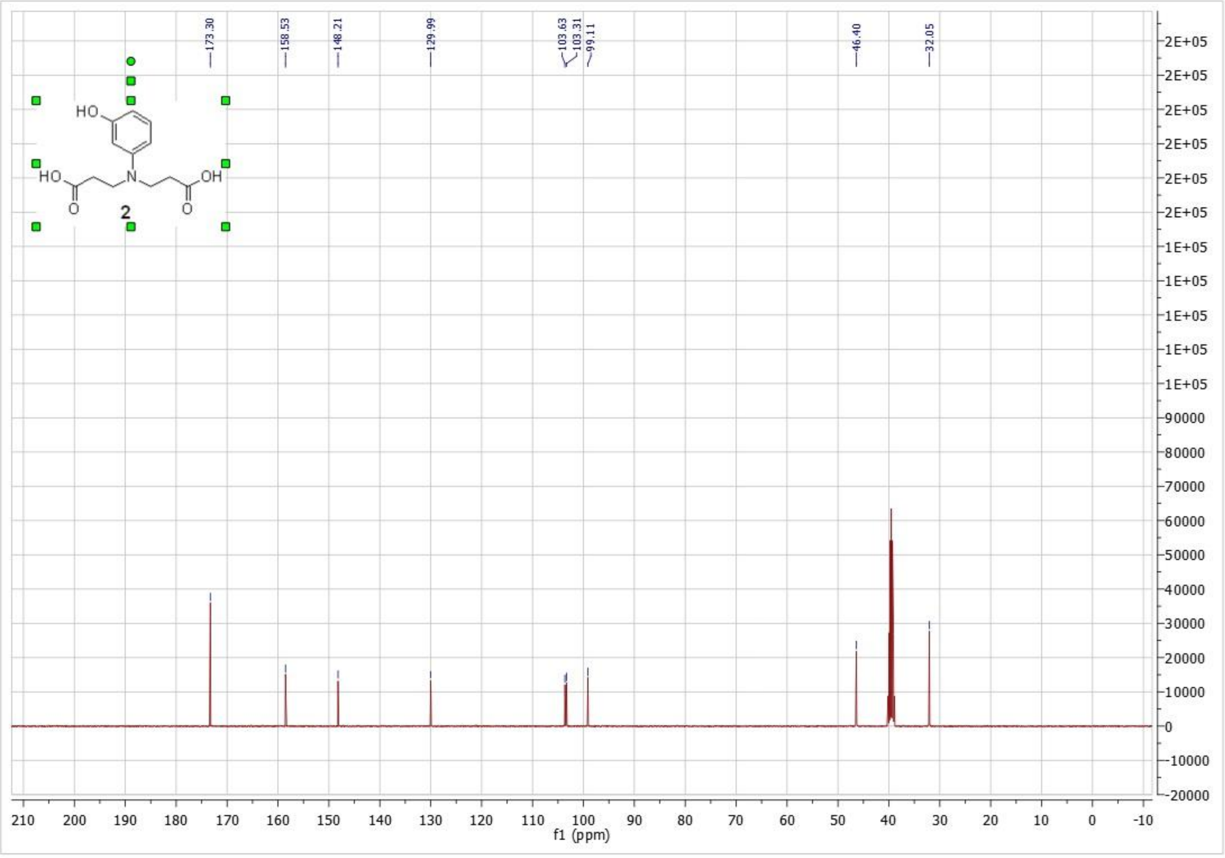

Figure S2. ^{13}C NMR spectrum of compound **2**

Dimethyl 3,3'-((3-hydroxyphenyl)azanediyl)dipropionate ***(3)***

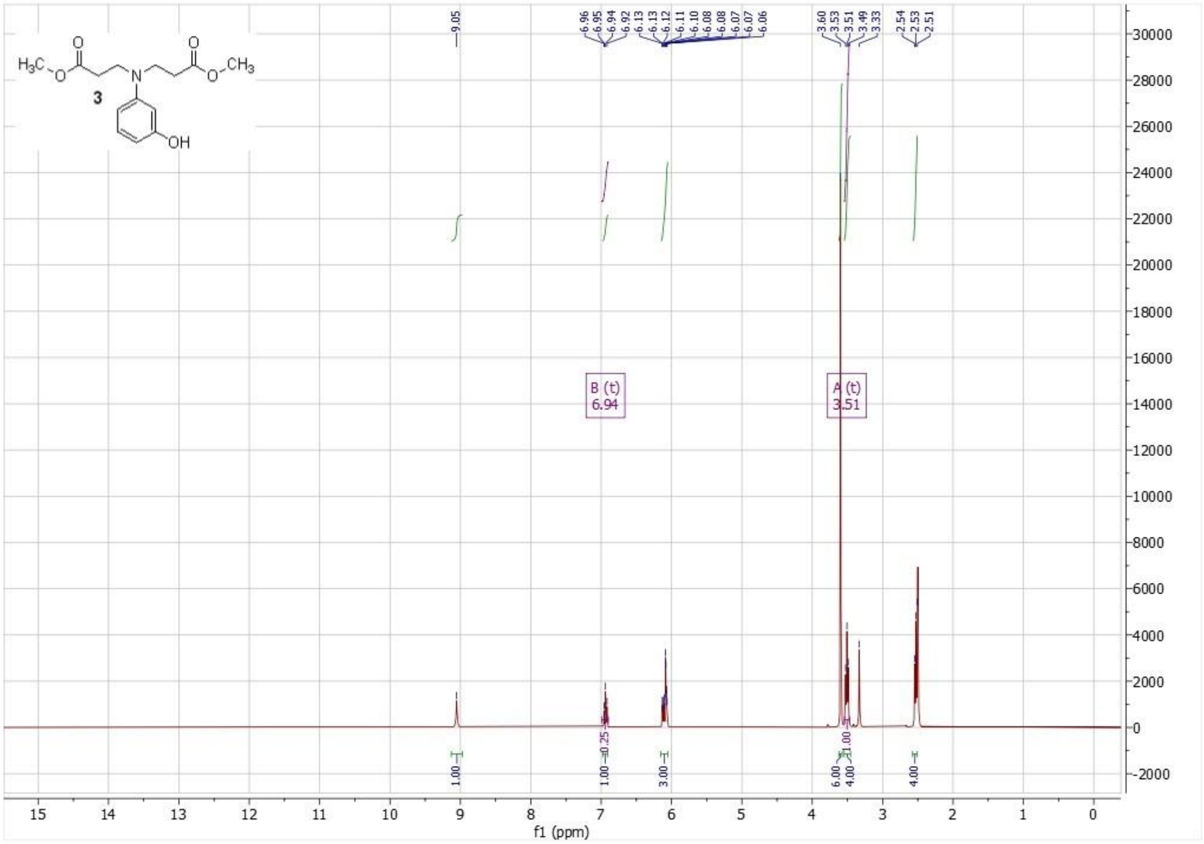

Figure S3. ^{1}H NMR spectrum of compound **3**

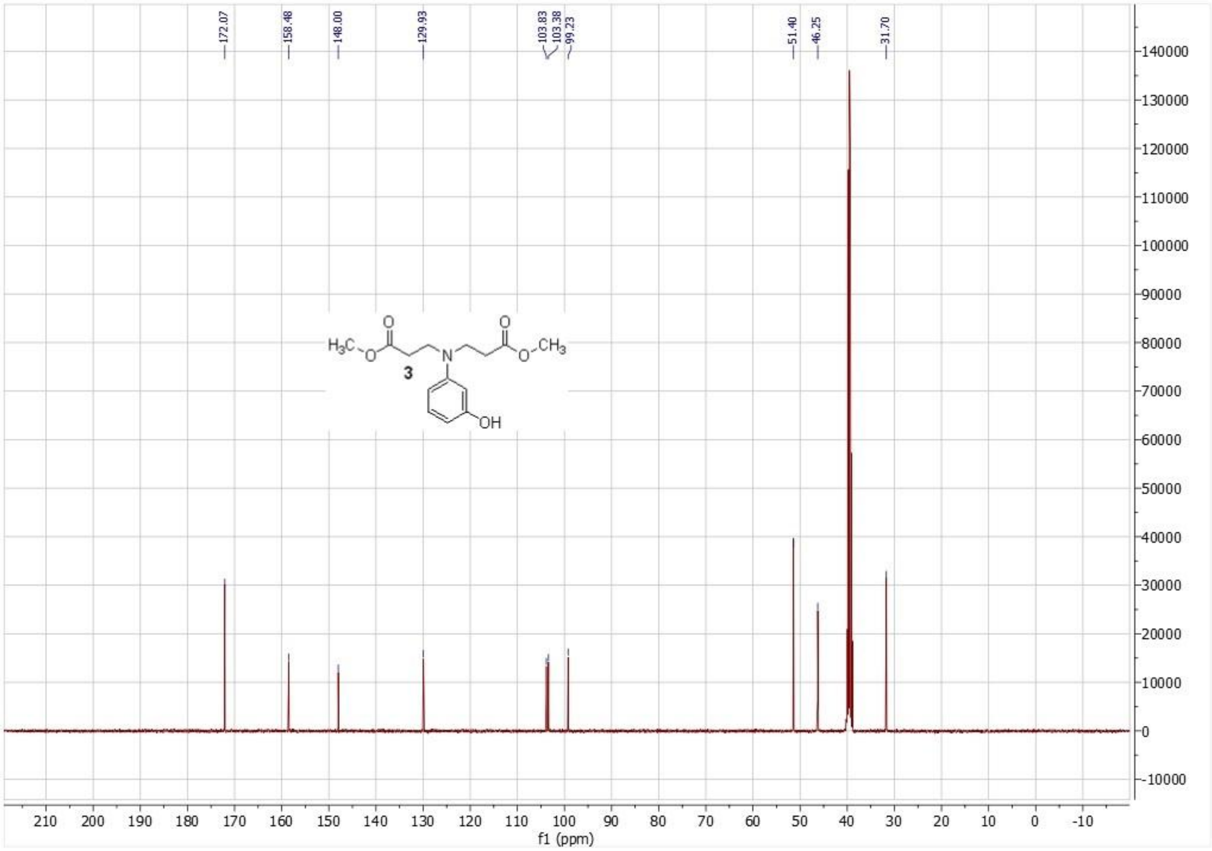

Figure S4. ^{13}C NMR spectrum of compound **3**

3,3'-((3-Hydroxyphenyl)azanediyl)di(propanehydrazide) ***(4)***

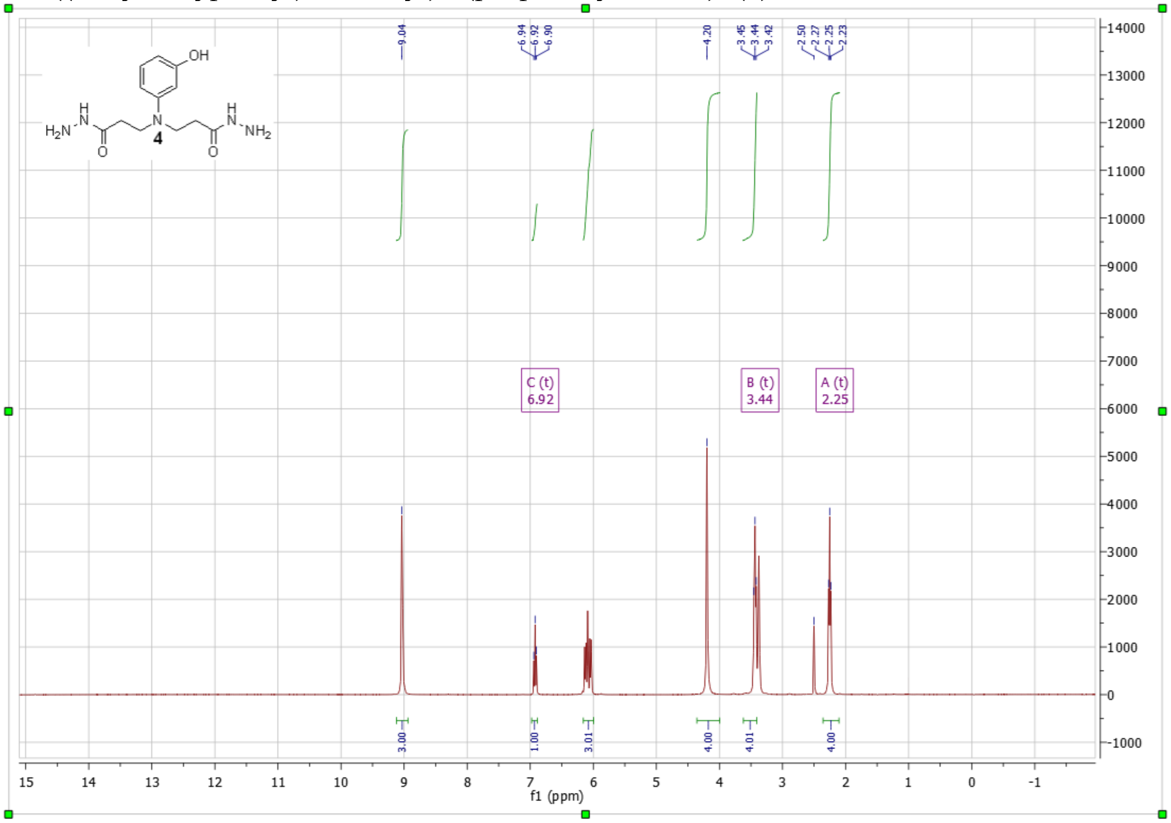

Figure S5. ^{1}H NMR spectrum of compound **4**

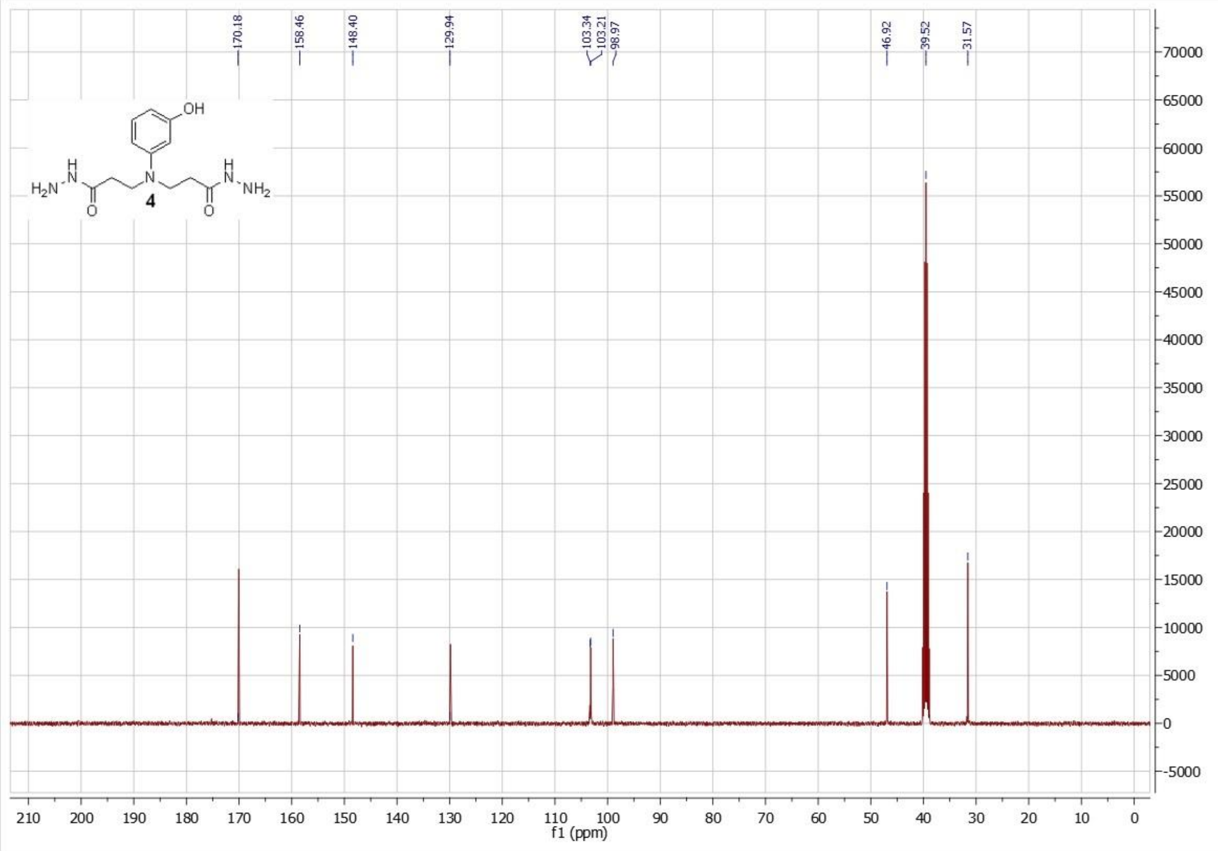

Figure S6. ^{13}C NMR spectrum of compound **4**

3,3'-((3-Hydroxyphenyl)azanediyl)bis(N'-(benzylidene)propanehydrazide) ***(8)***

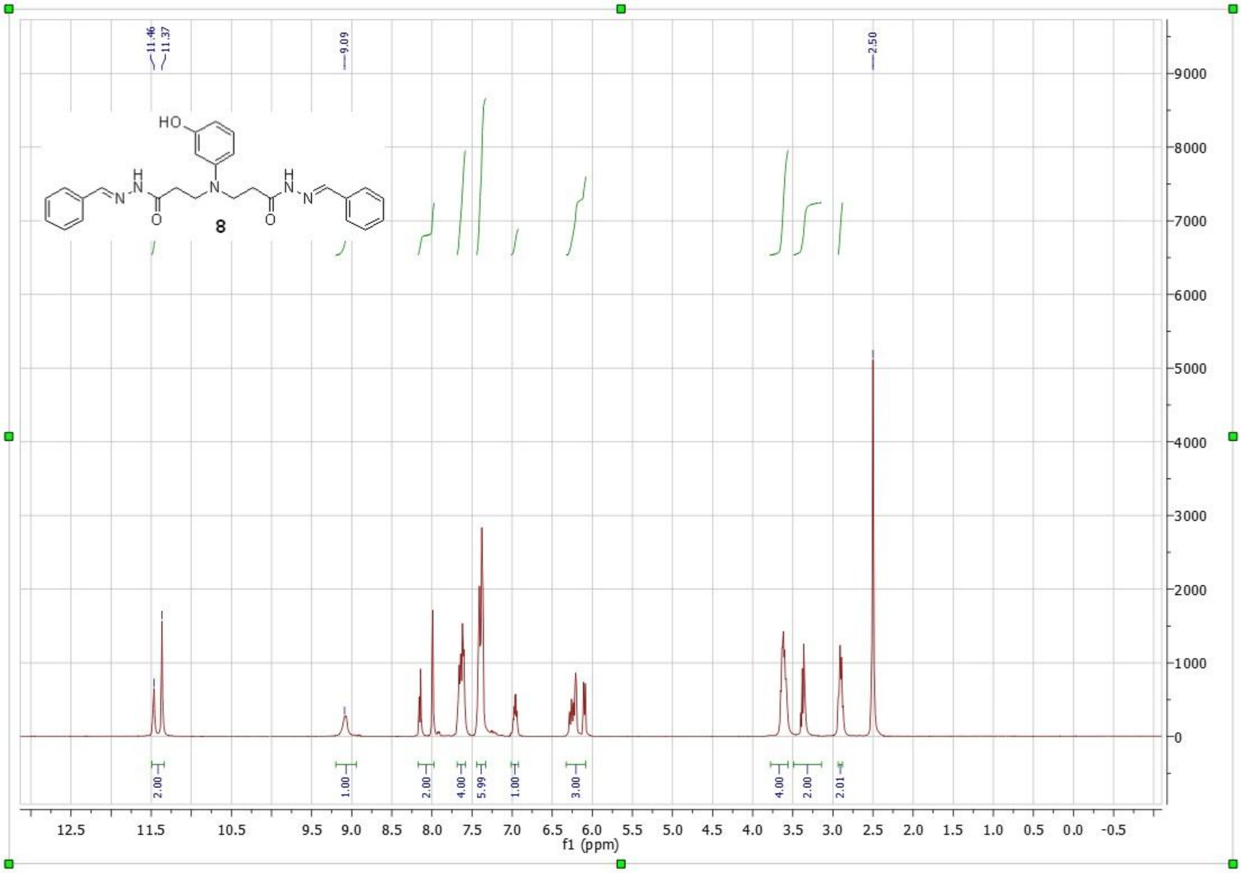

Figure S7. ^{1}H NMR spectrum of compound **8**

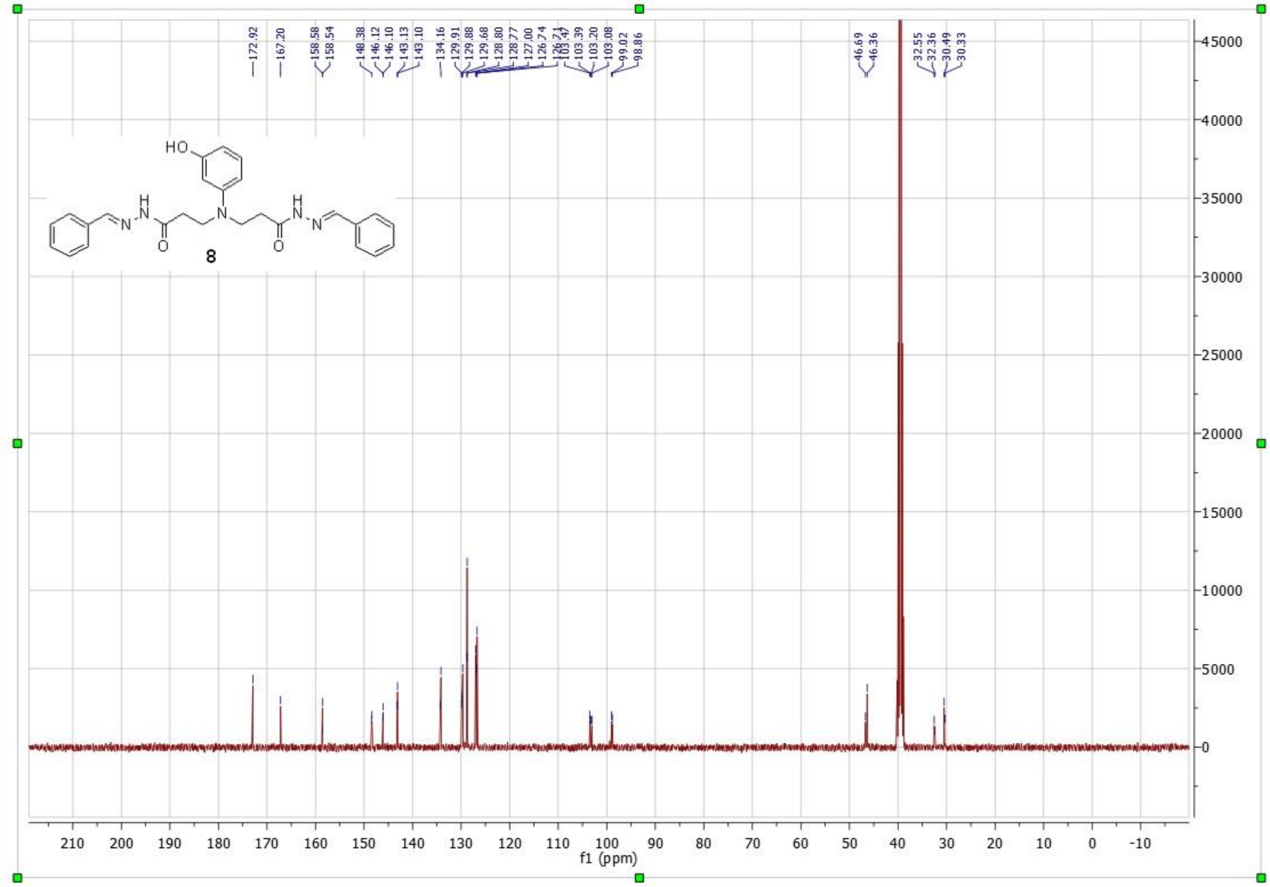

Figure S8. ^{13}C NMR spectrum of compound **8**

3,3'-((3-Hydroxyphenyl)azanediyl)bis(N'-(2,4-difluorobenzylidene)propanehydrazide) ***(9)***

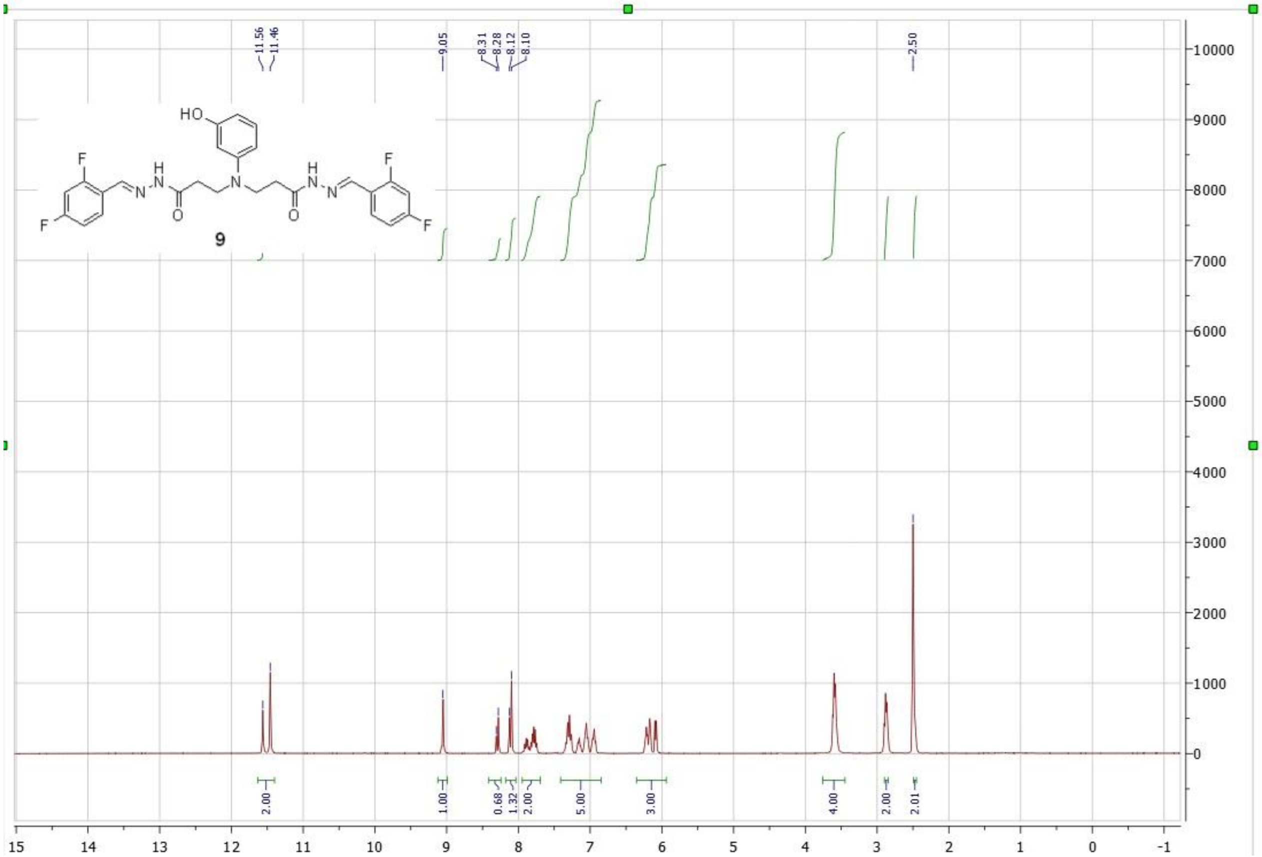

Figure S9. ^{1}H NMR spectrum of compound **9**

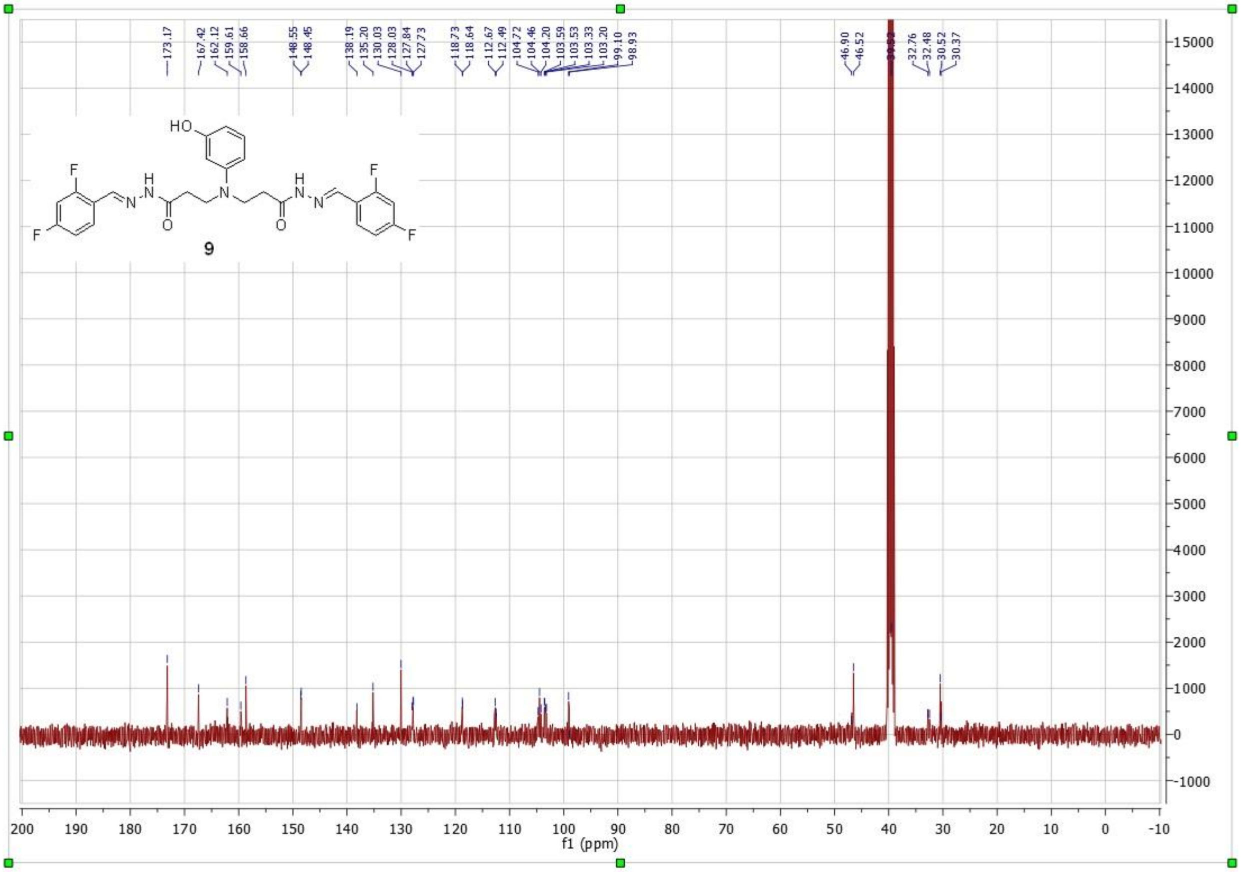

Figure S10. ^{13}C NMR spectrum of compound **9**

3,3'-((3-Hydroxyphenyl)azanediyl)bis(N'-(4-nitrobenzylidene)propanehydrazide) ***(10)***

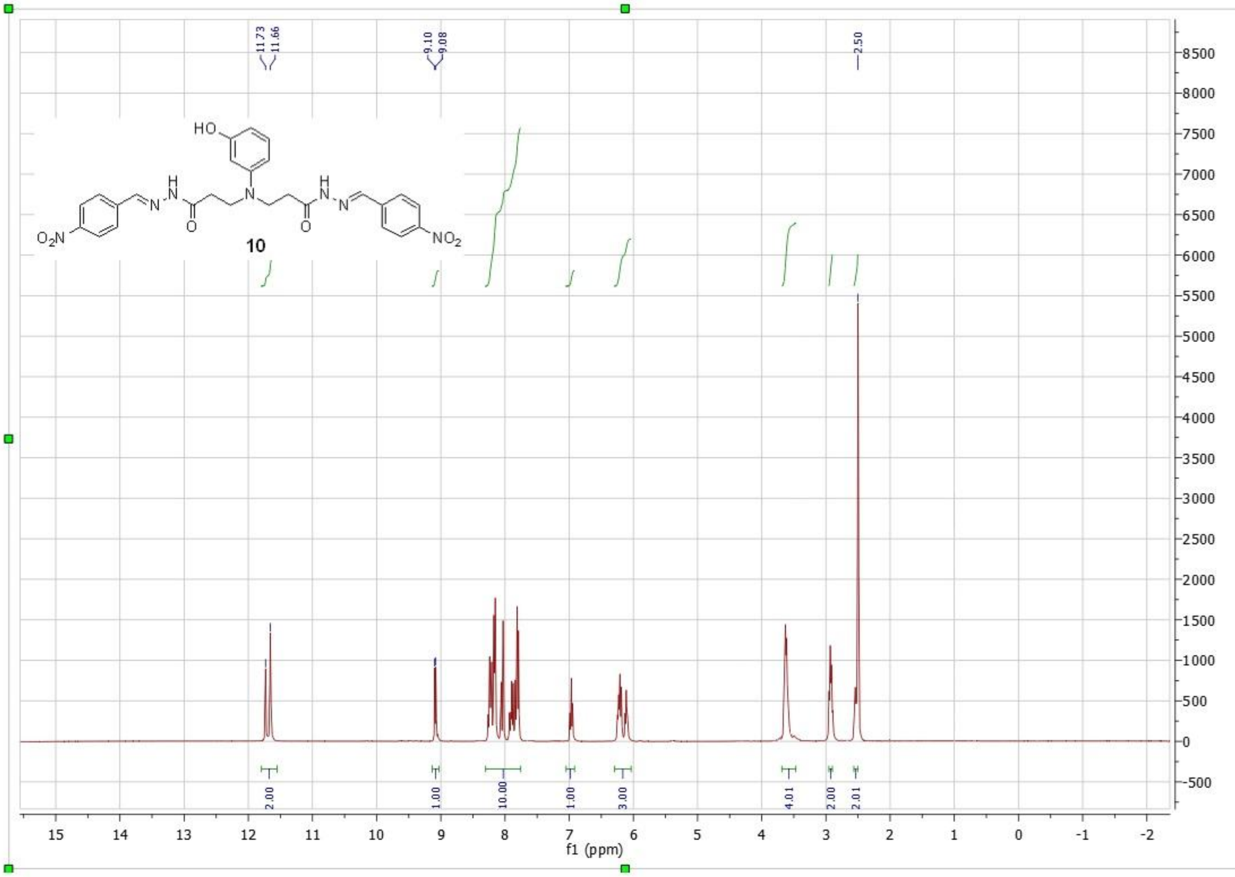

Figure S11. ^{1}H NMR spectrum of compound **10**

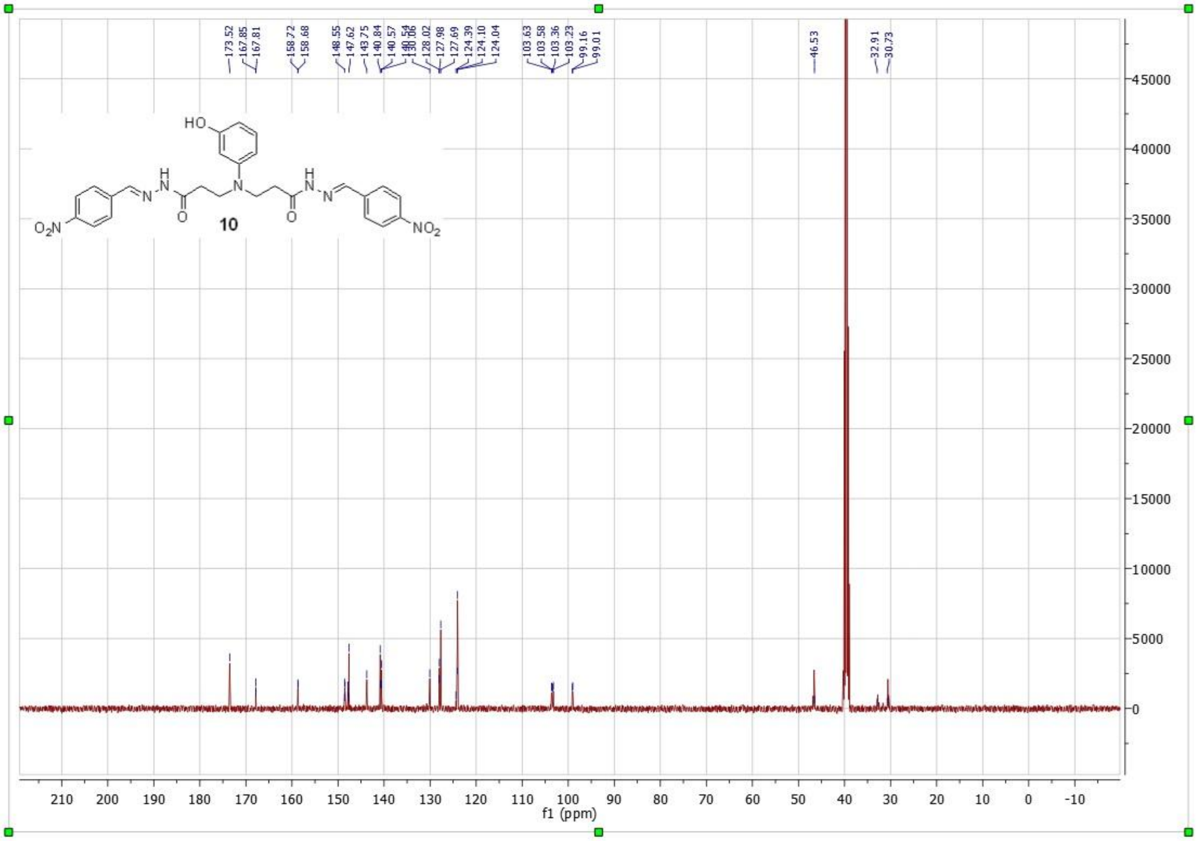

Figure S12. ^{13}C NMR spectrum of compound **10**

3,3'-((3-Hydroxyphenyl)azanediyl)bis(N'-(4-chlorobenzylidene)propanehydrazide) ***(11)***

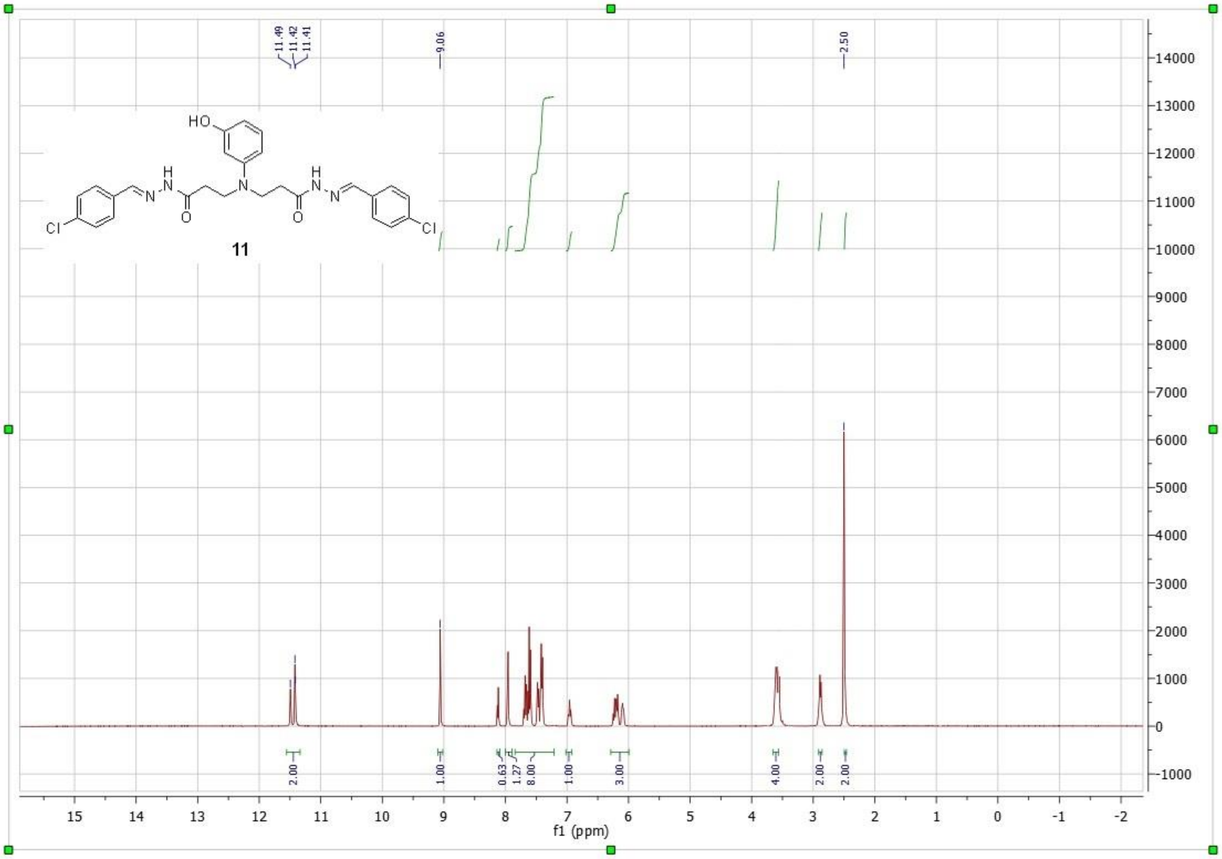

Figure S13. ^{1}H NMR spectrum of compound **11**

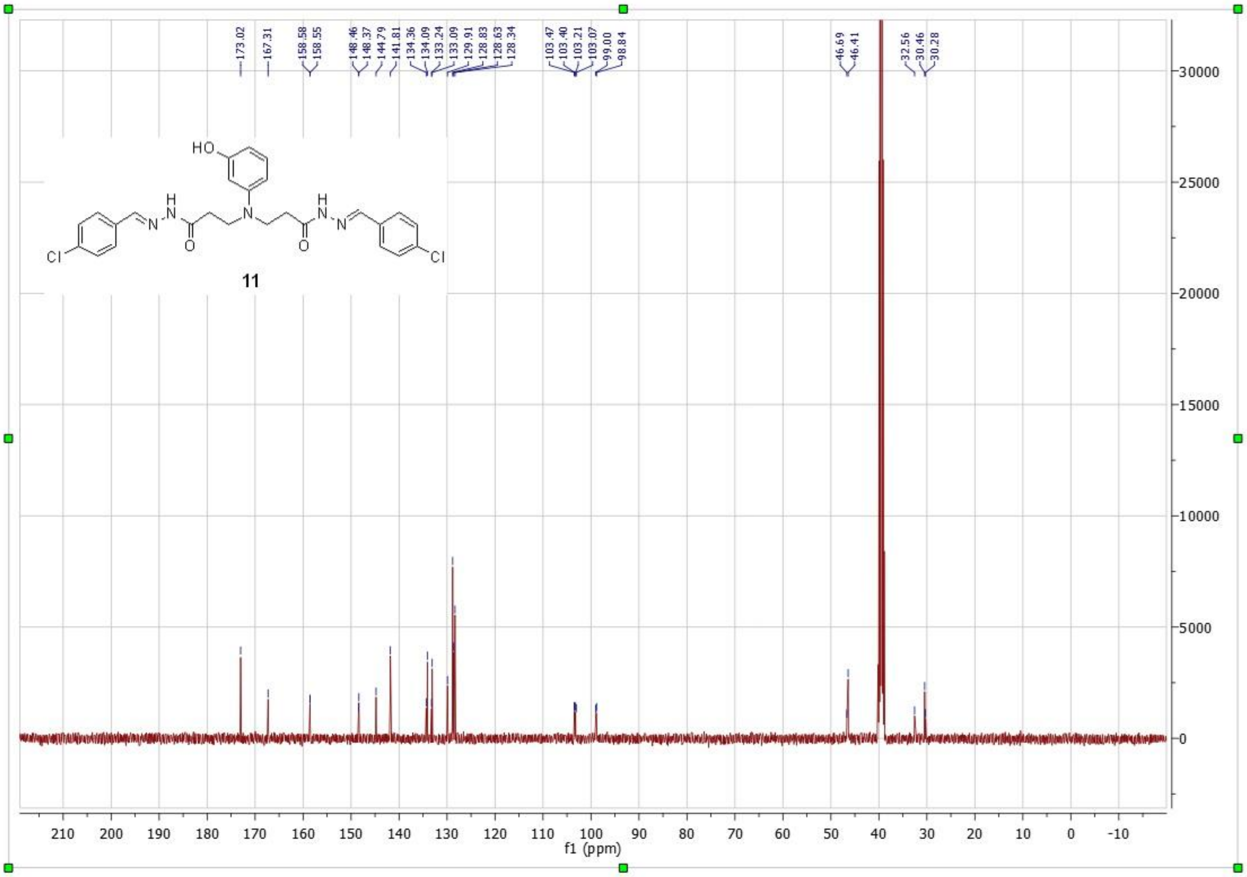

Figure S14. ^{13}C NMR spectrum of compound **11**

3,3'-((3-Hydroxyphenyl)azanediyl)bis(N'-(4-dimethylamino)benzylidene)propanehydrazide) ***(12)***

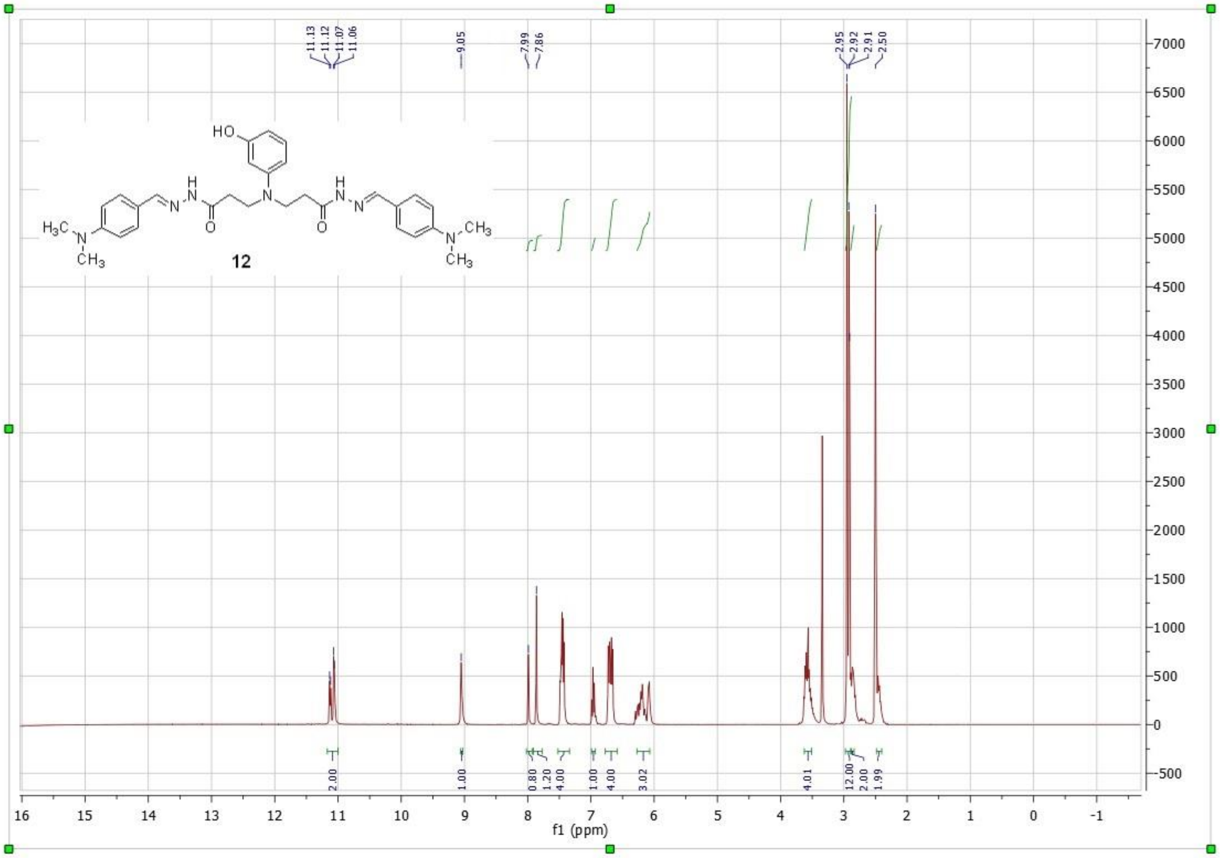

Figure S15. ^{1}H NMR spectrum of compound **12**

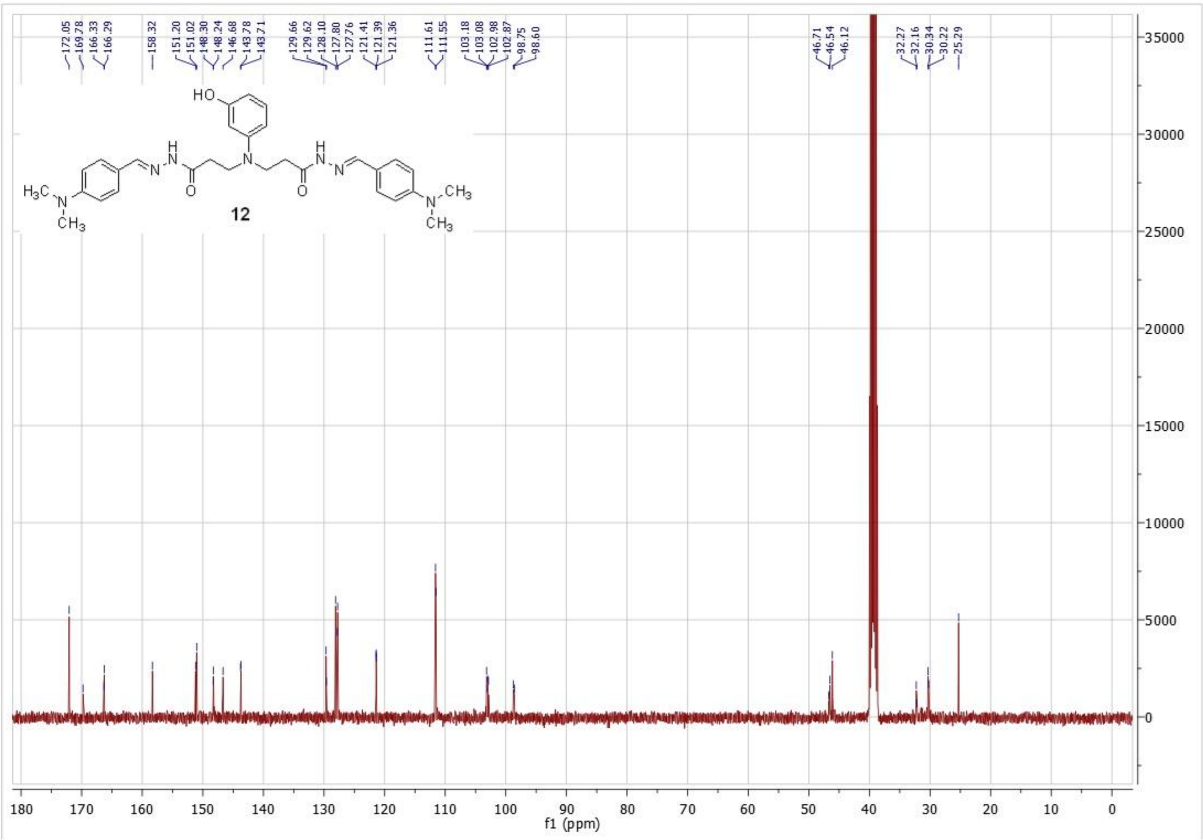

Figure S16. ^{13}C NMR spectrum of compound **12**

3,3'-((3-Hydroxyphenyl)azanediyl)bis(N'-(3,4,5-trimethoxybenzylidene)propanehydrazide) ***(13)***

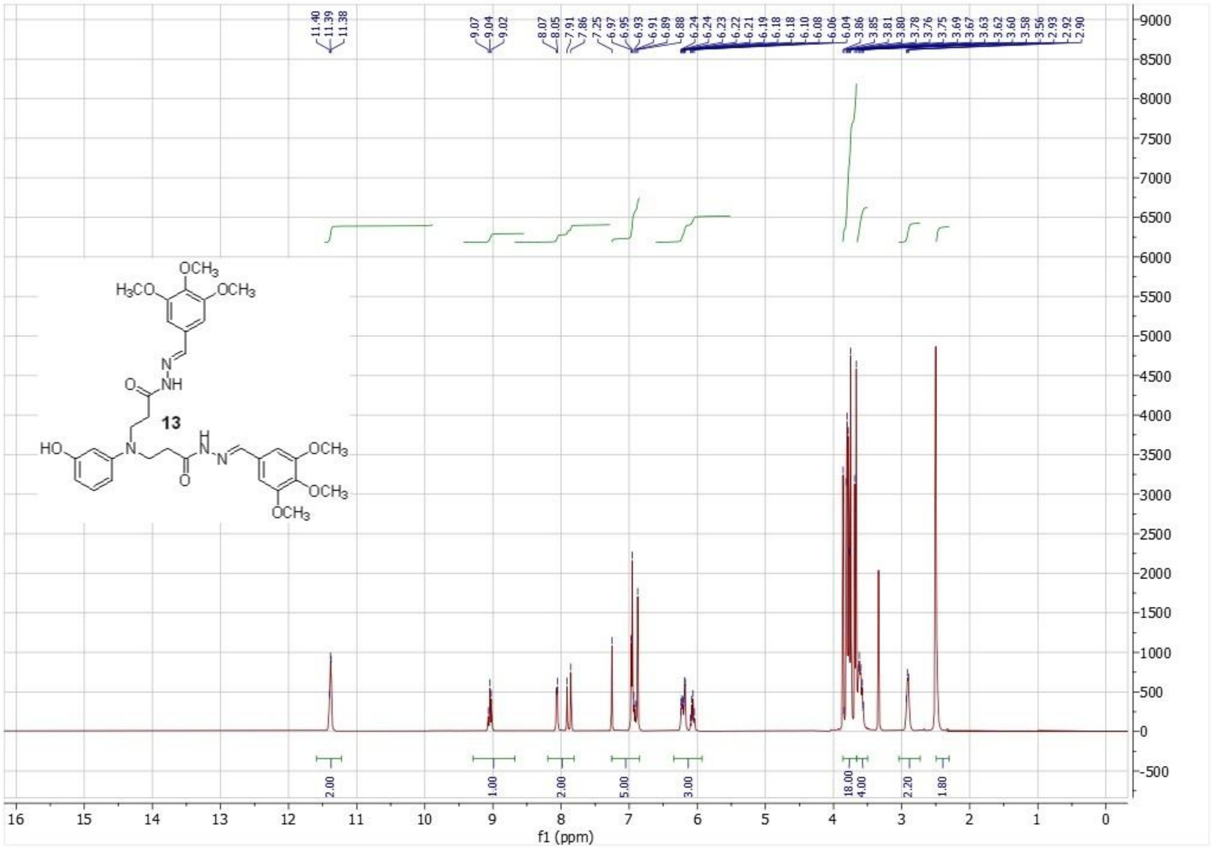

Figure S17. ^{1}H NMR spectrum of compound **13**

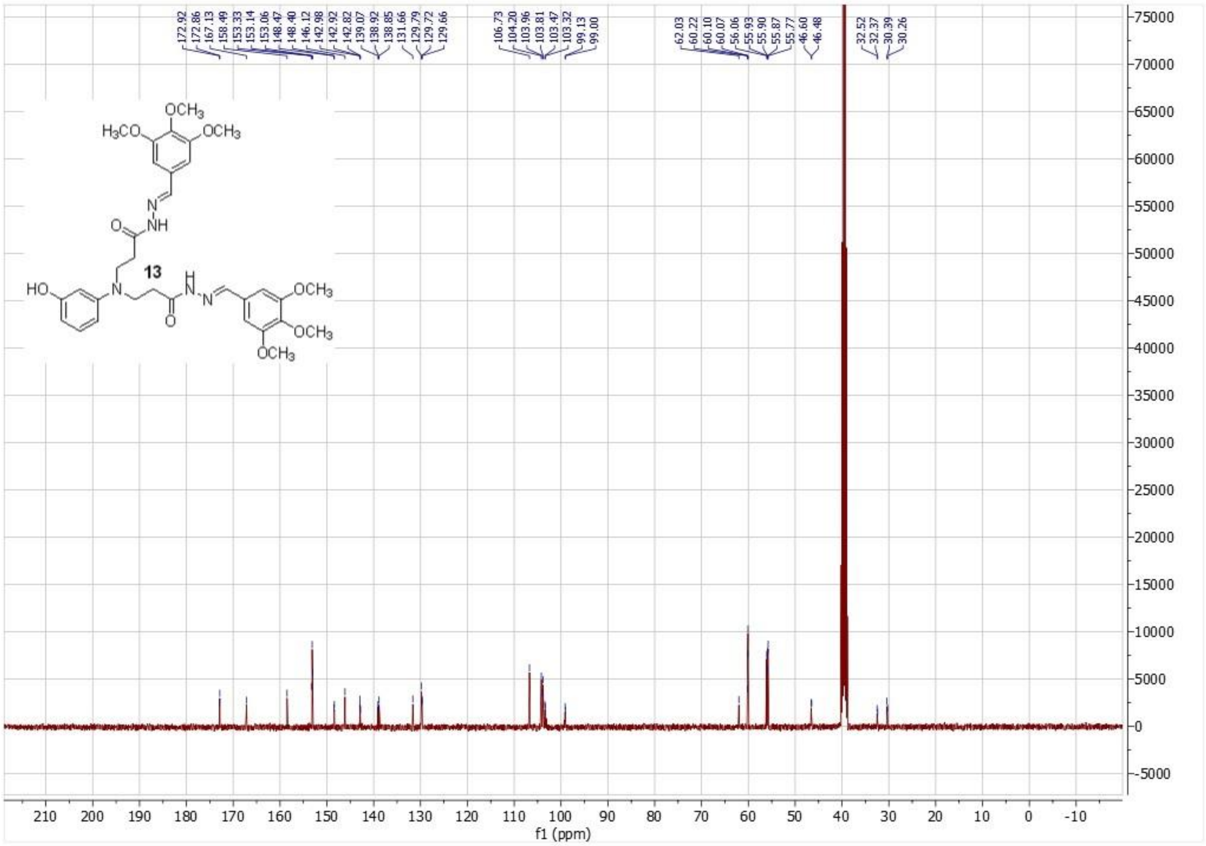

Figure S18. ^{13}C NMR spectrum of compound **13**

3,3'-((3-Hydroxyphenyl)azanediyl)bis(N'-(naphthalen-1-ylmethylene)propanehydrazide) ***(14)***

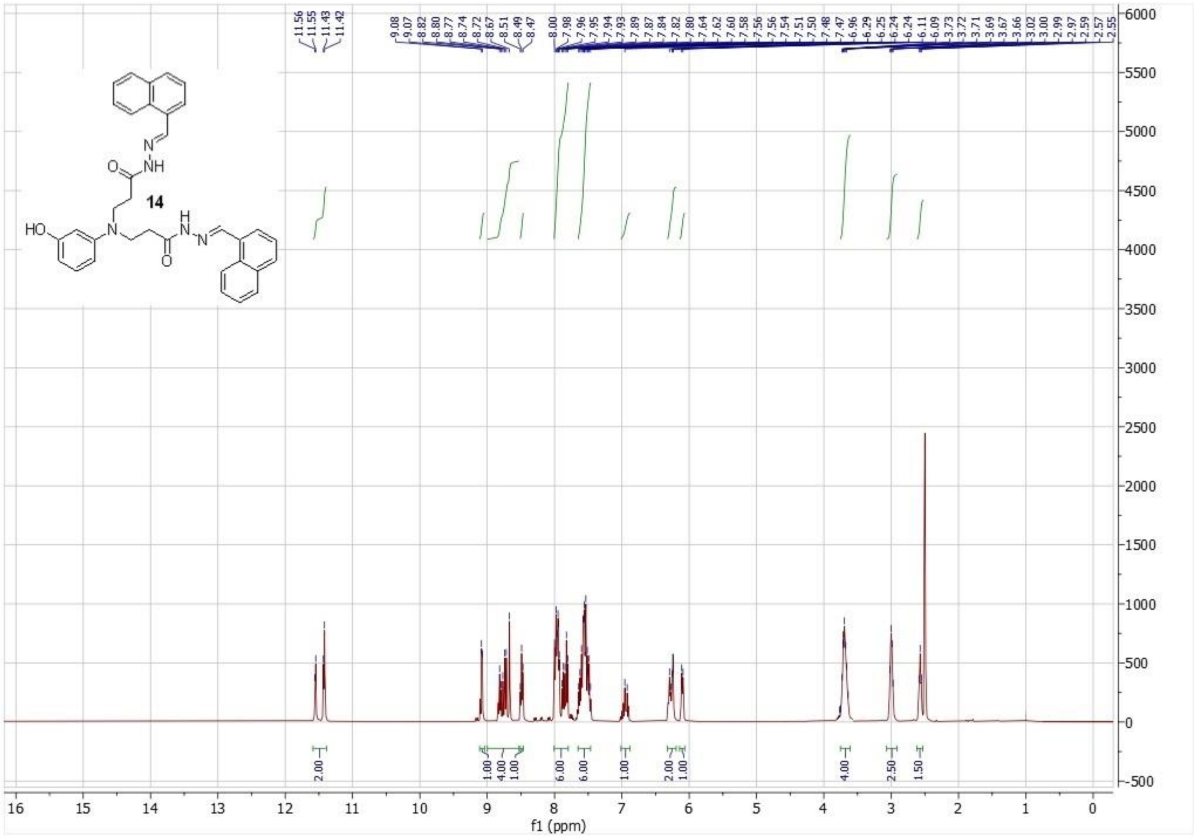

Figure S19. ^{1}H NMR spectrum of compound **14**

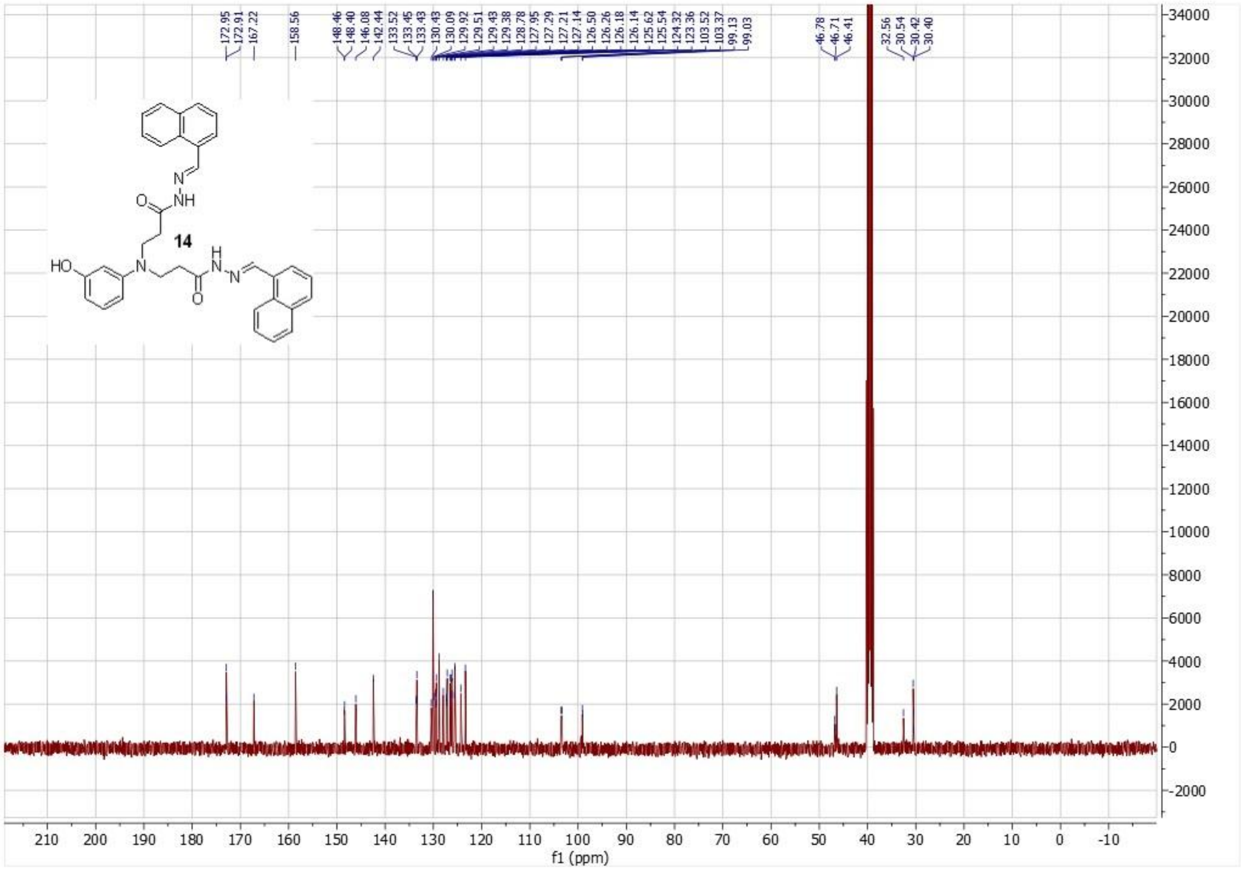

Figure S20. ^{13}C NMR spectrum of compound **14**

3,3'-((3-Hydroxyphenyl)azanediyl)bis(N'-(furan-2-ylmethylene)benzylidene)propanehydrazide)) ***(15)***

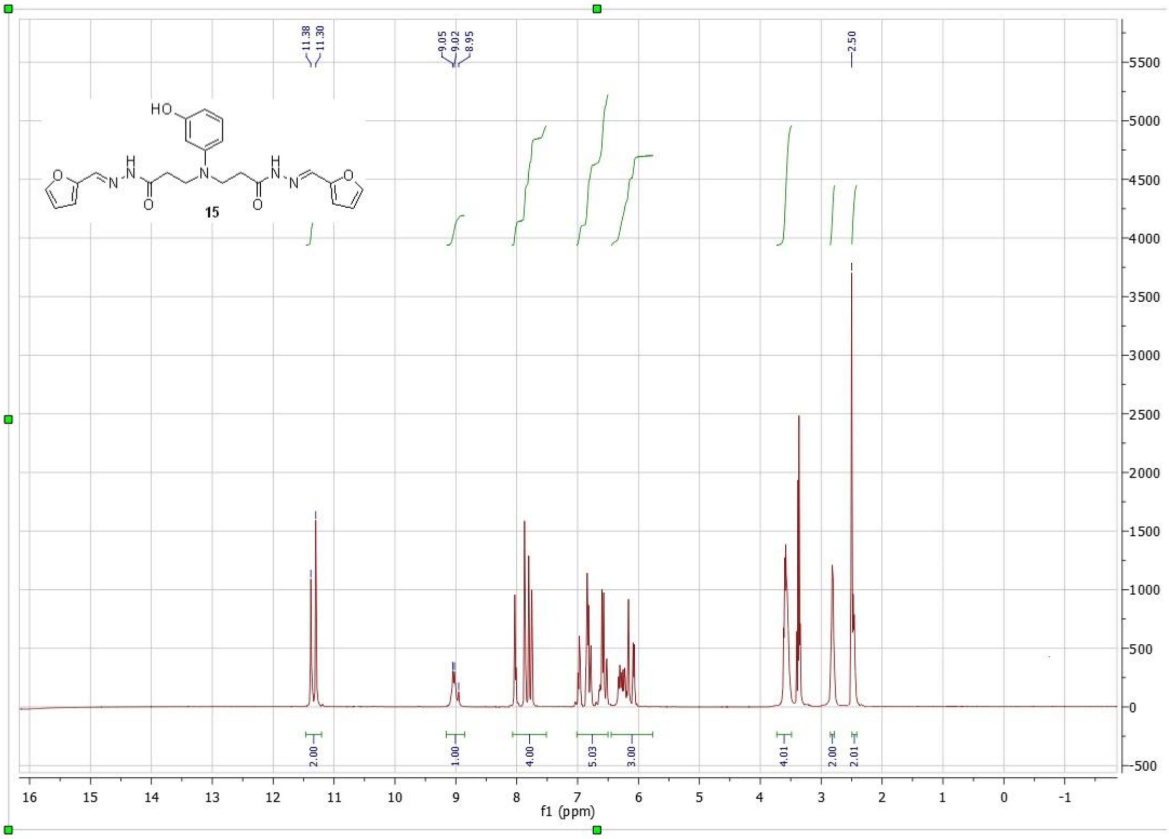

Figure S21. ^{1}H NMR spectrum of compound **15**

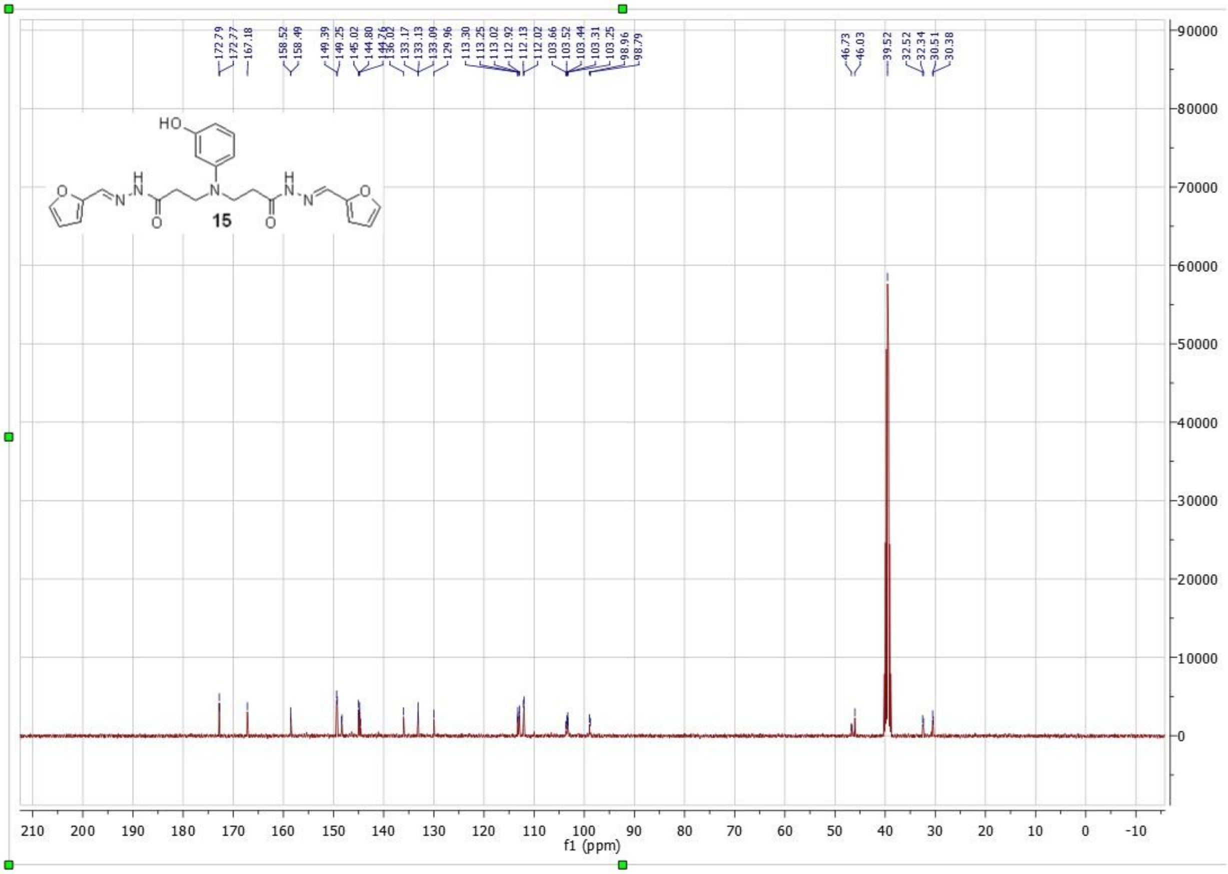

Figure S22. ^{13}C NMR spectrum of compound **15**

3,3'-((3-Hydroxyphenyl)azanediyl)bis(N'-((5-nitrothiophen-2-yl)methylene)propanehydrazide) ***(16)***

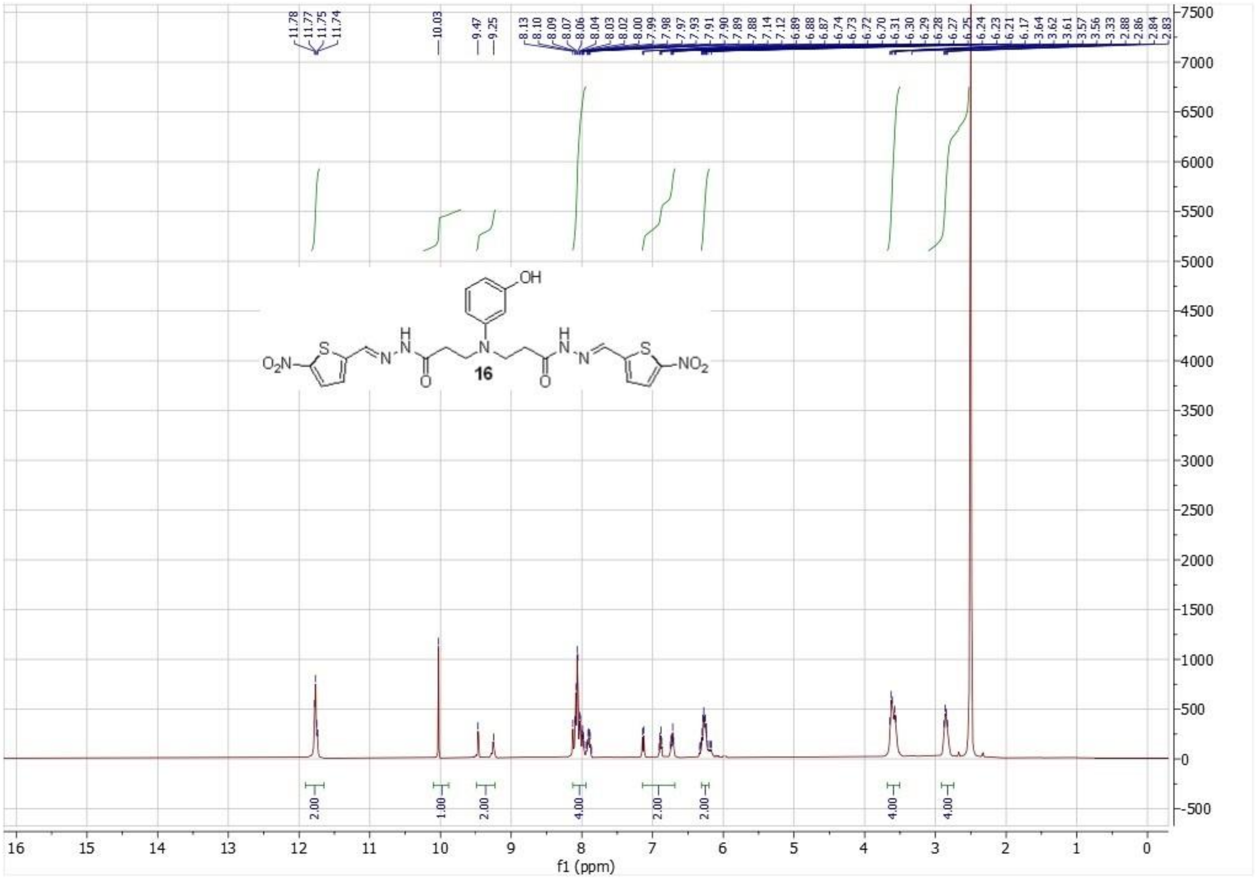

Figure S23. ^{1}H NMR spectrum of compound **16**

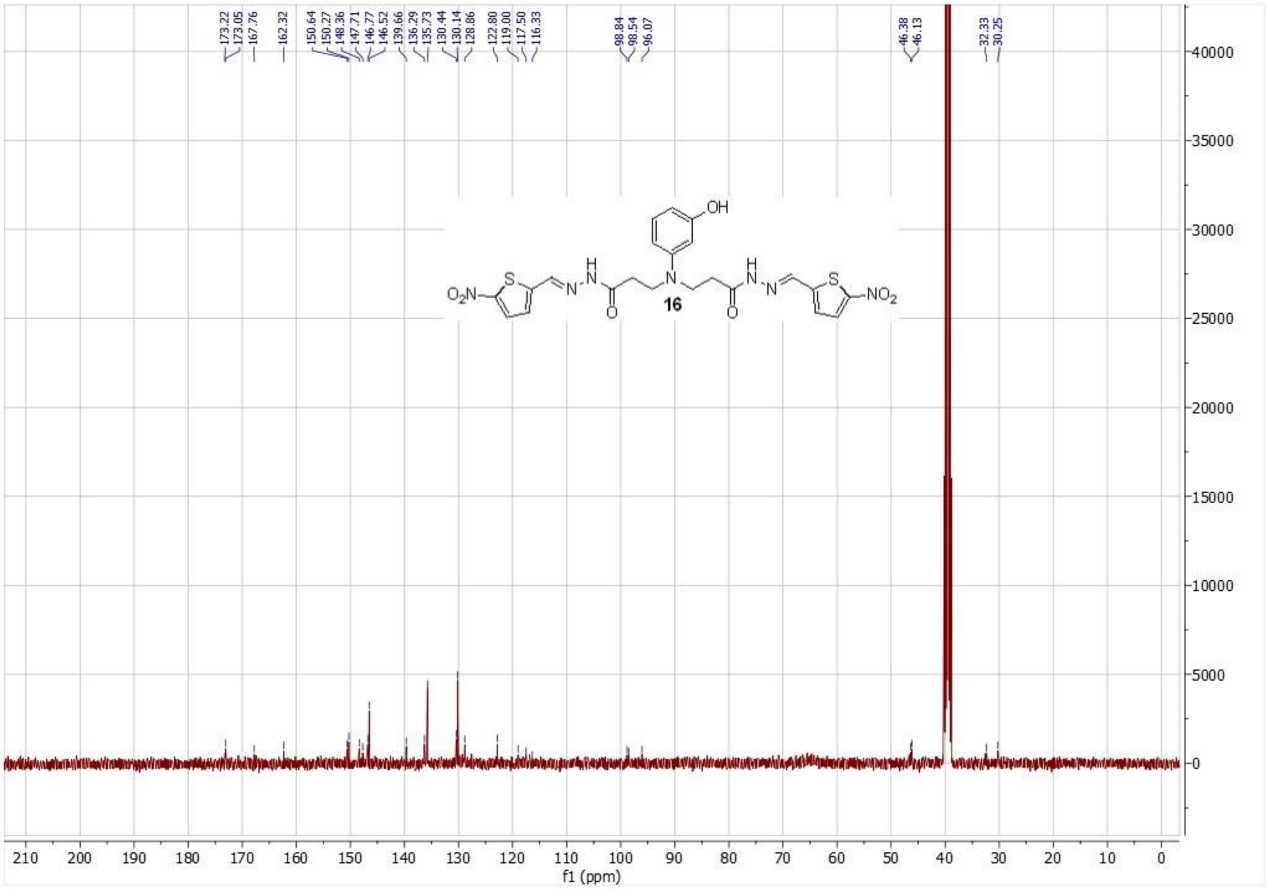

Figure S24. ^{13}C NMR spectrum of compound **16**

3,3'-((3-Hydroxyphenyl)azanediyl)bis(N'-(5-nitrofuran-2-yl)methylene)benzylidene)propanehydrazide) ***(17)***

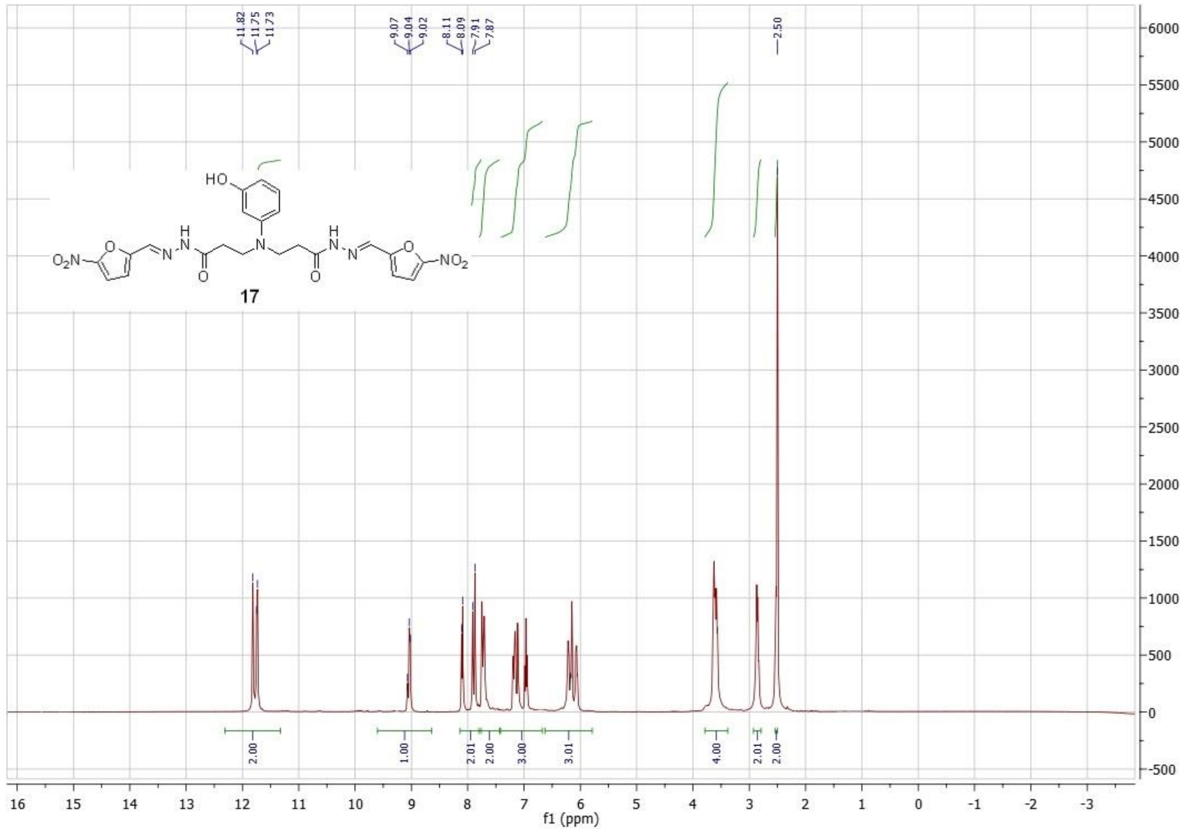

Figure S25. ^{1}H NMR spectrum of compound **17**

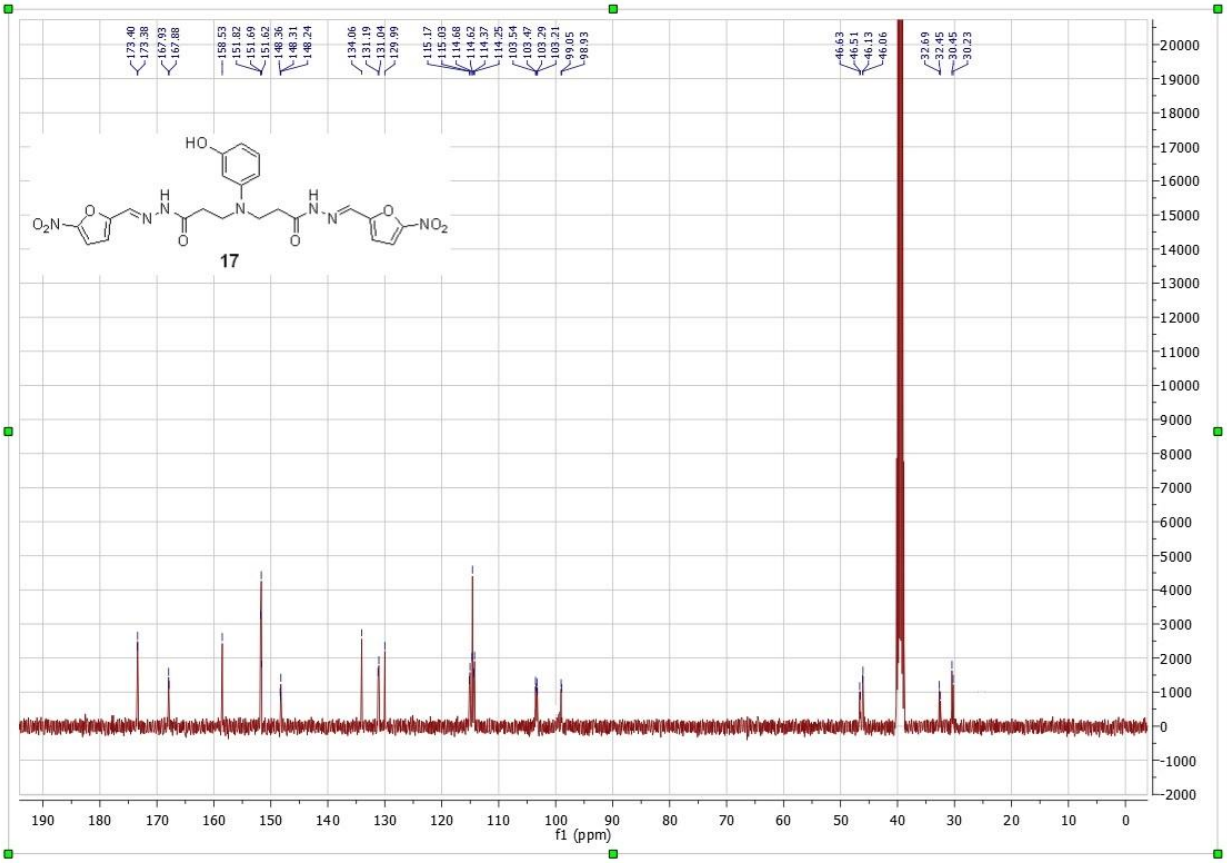

Figure S26. ^{13}C NMR spectrum of compound **17**

3,3'-((3-Hydroxyphenyl)azanediyl)bis(N'-(thiophen-3-yl)methylene)benzylidene)propanehydrazide) ***(18)***

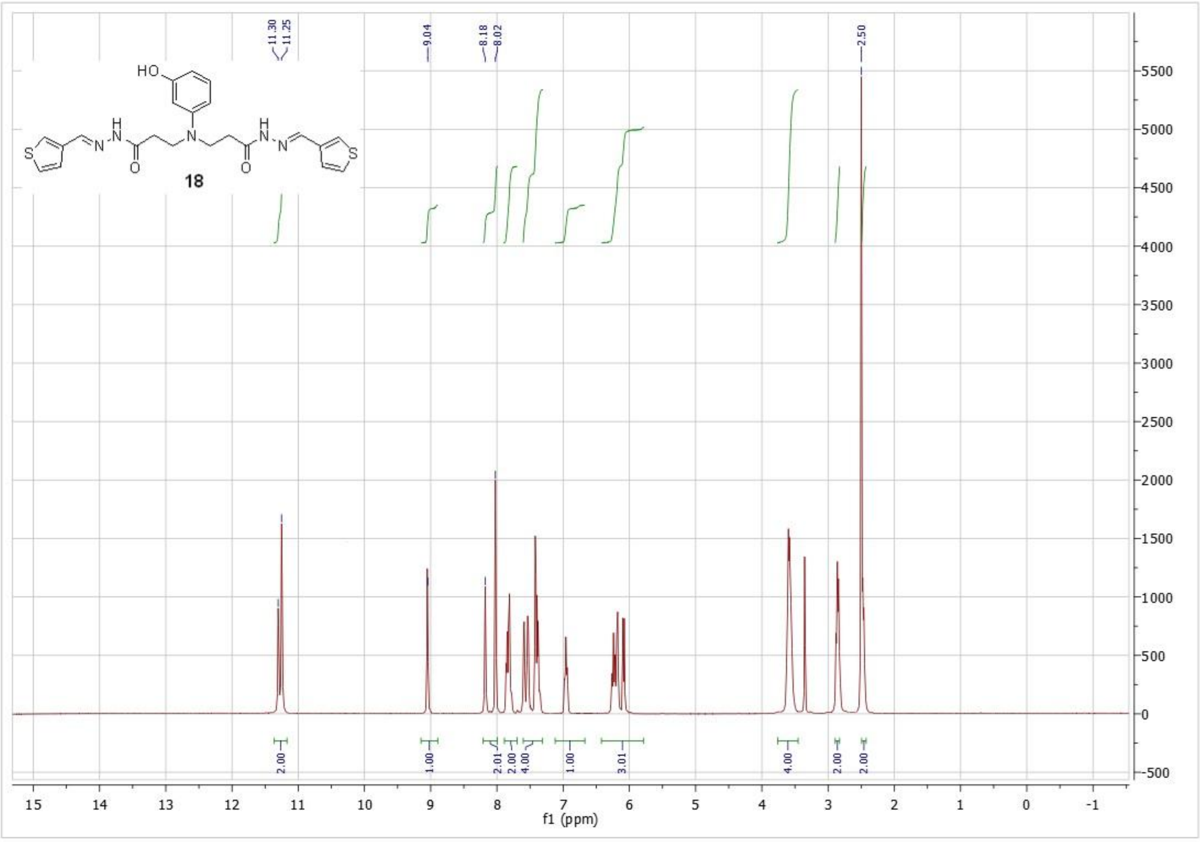

Figure S27. ^{1}H NMR spectrum of compound **18**

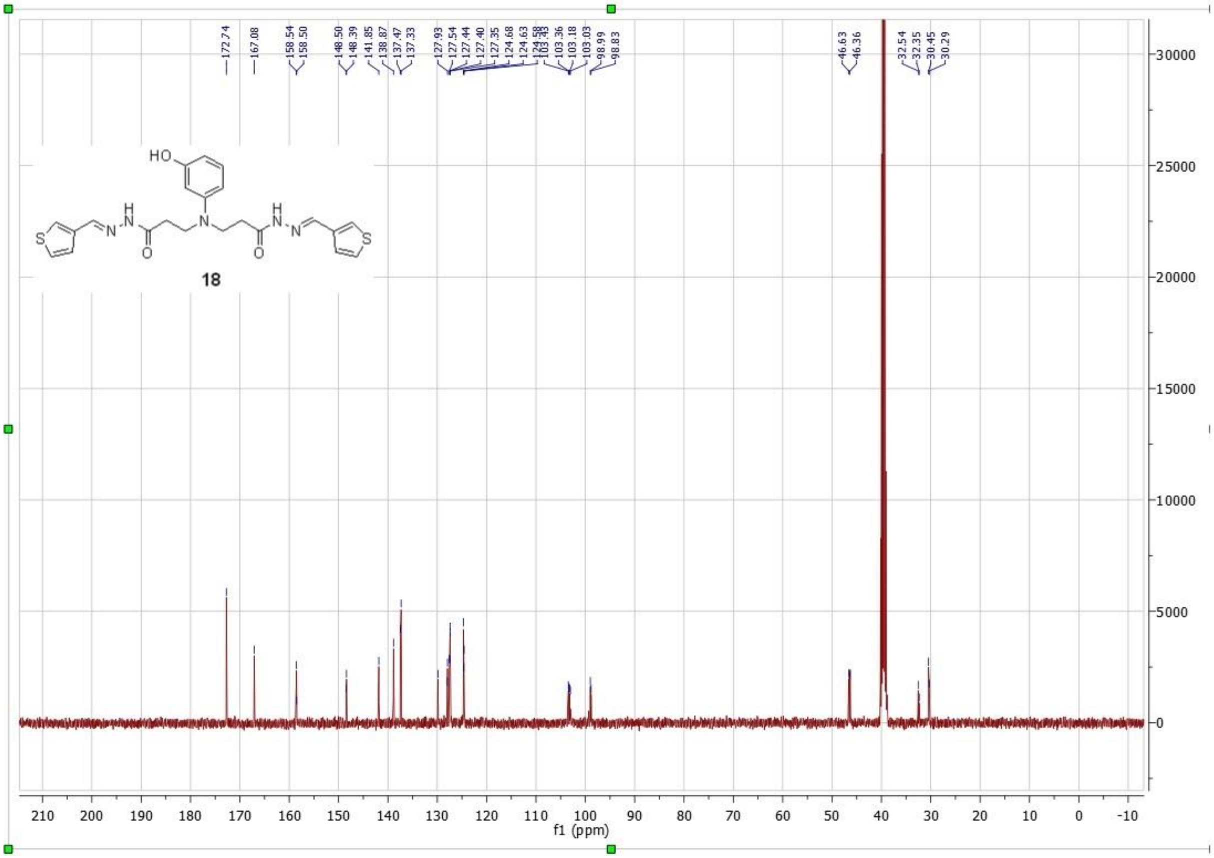

Figure S28. ^{13}C NMR spectrum of compound **18**

3,3'-((3-Hydroxyphenyl)azanediyl)bis(N'-(propan-2-ylidene)propanehydrazide) ***(19)***

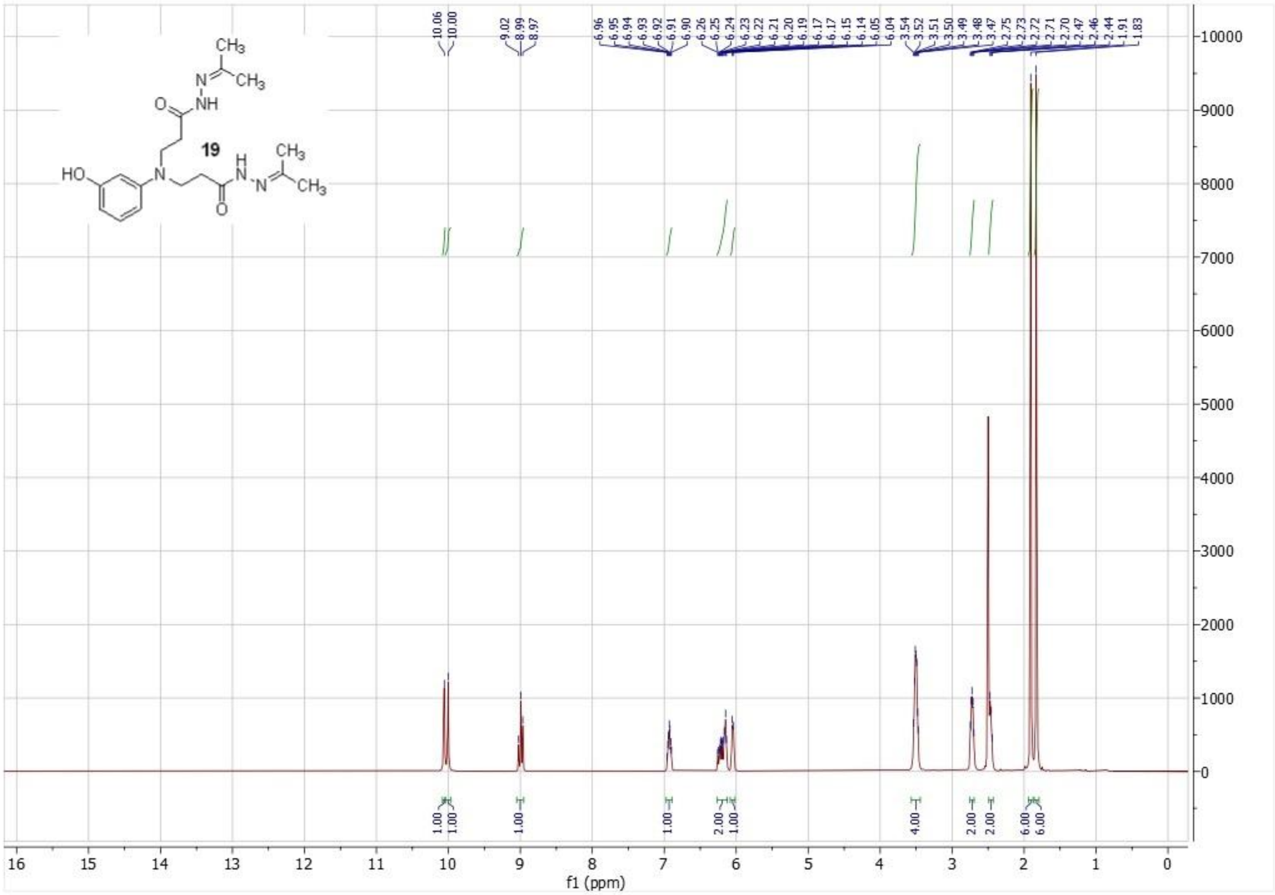

Figure S29. ^{1}H NMR spectrum of compound **19**

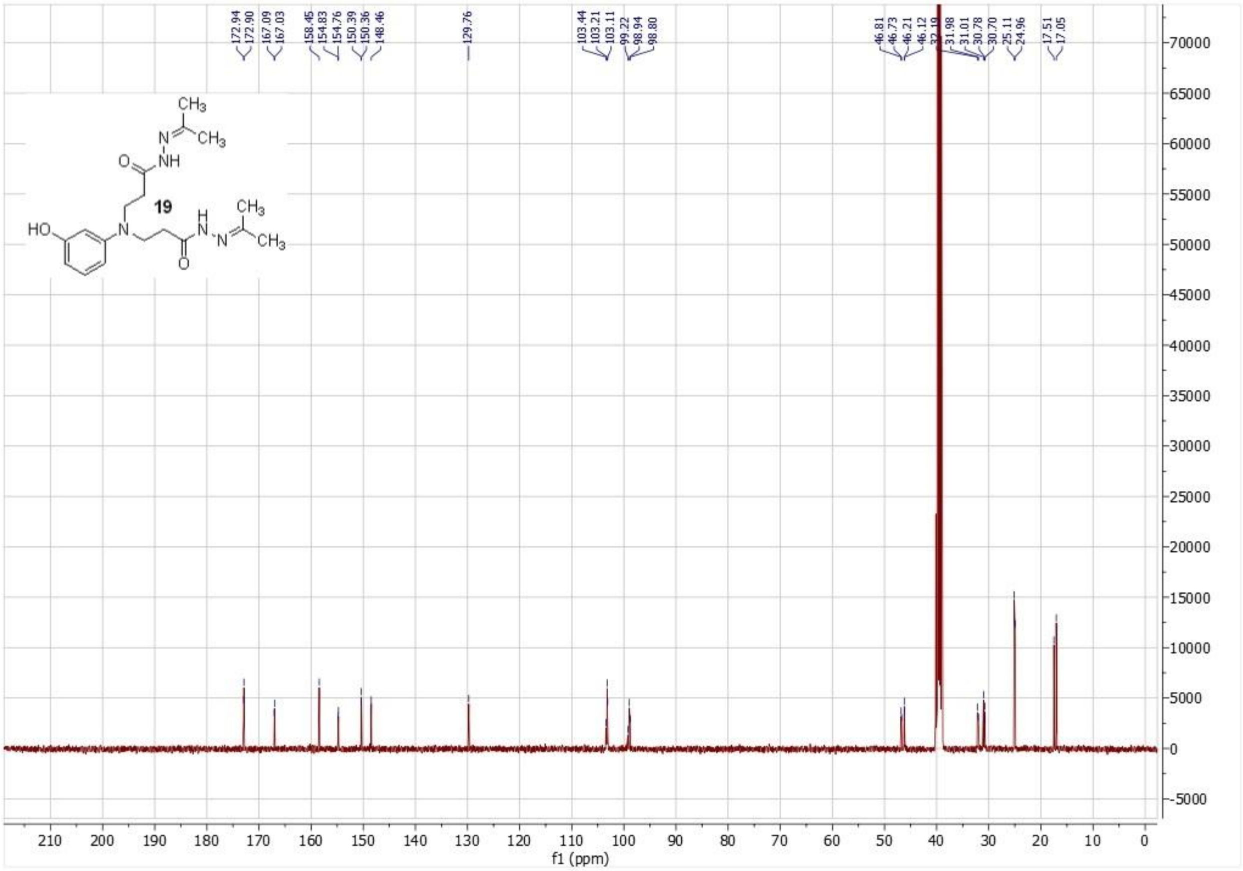

Figure S30. ^{13}C NMR spectrum of compound **19**

3,3'-((3-Hydroxyphenyl)azanediyl)bis(N'-(butan-2-ylidene)propanehydrazide) ***(20)***

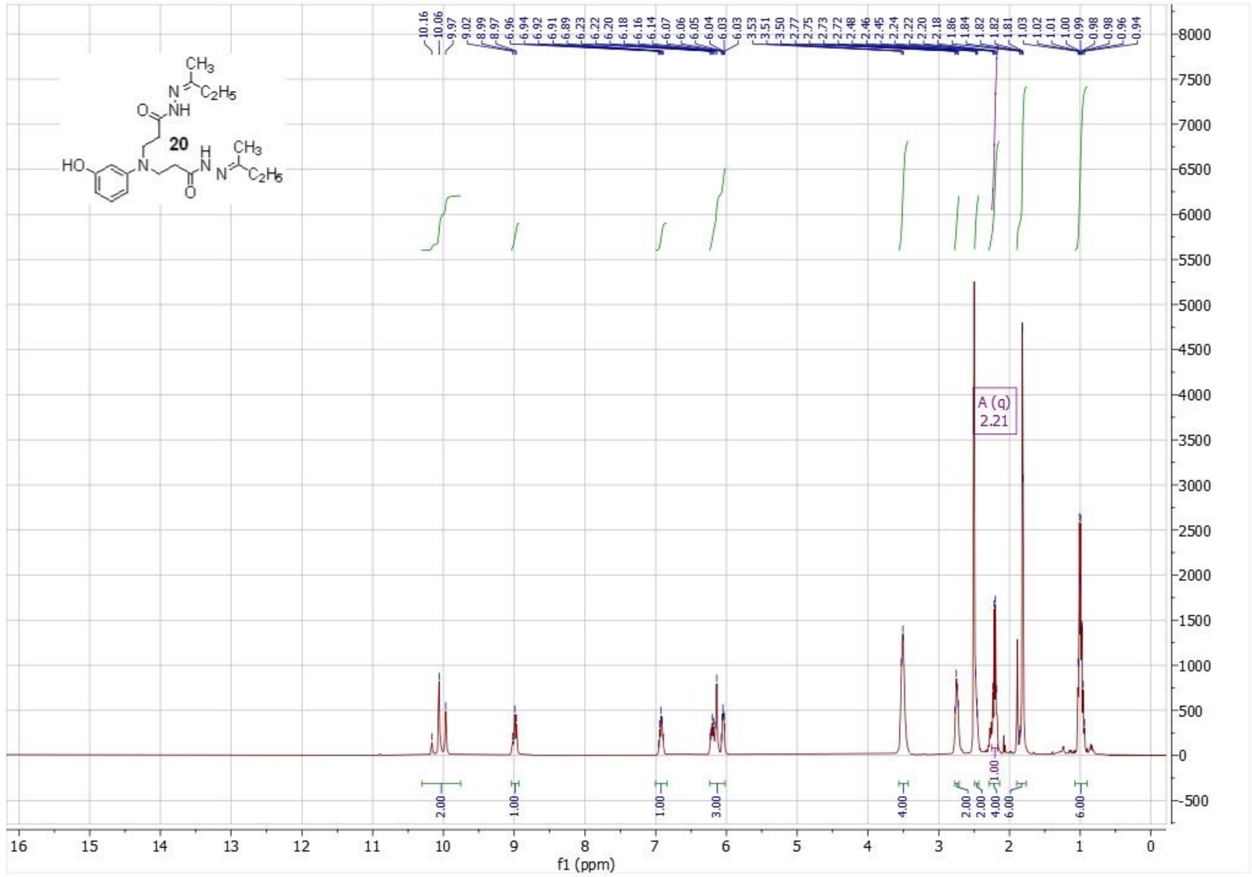

Figure S31. ^{1}H NMR spectrum of compound **20**

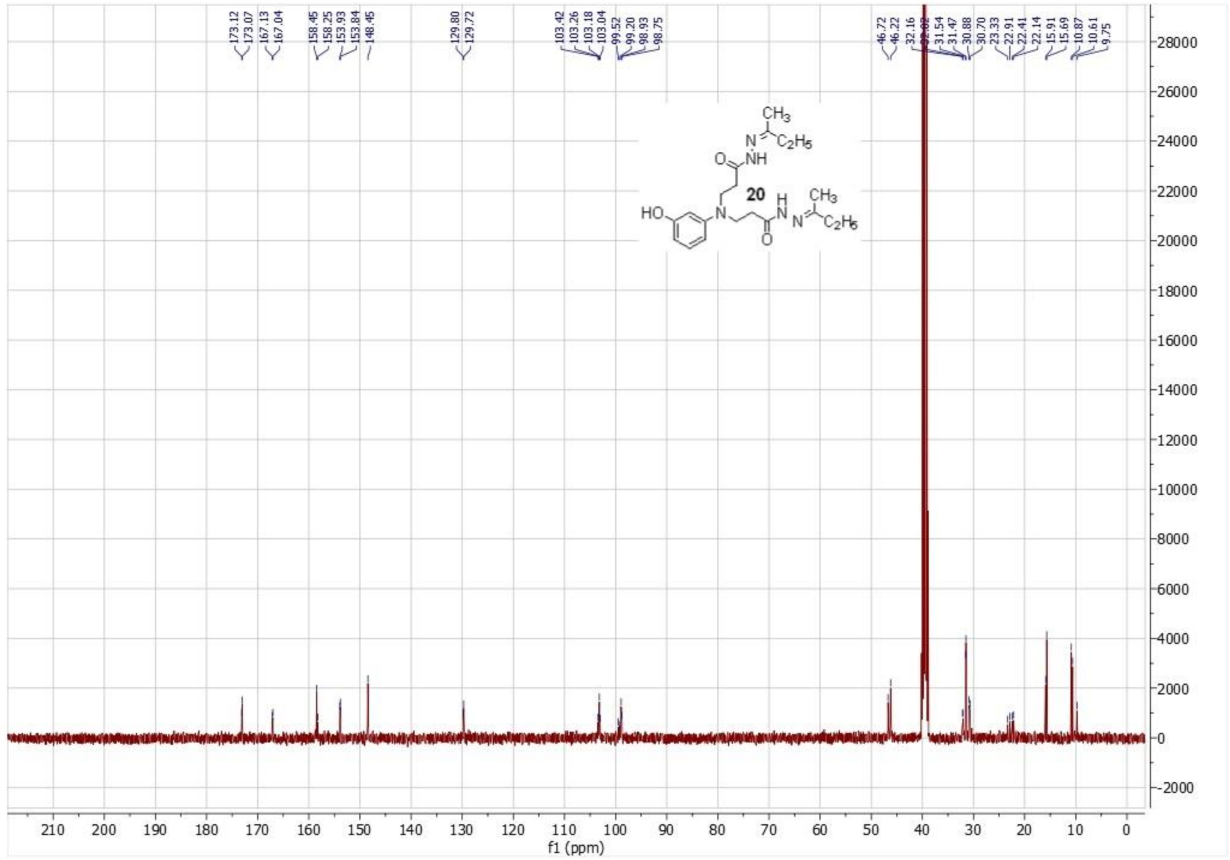

Figure S32. ^{13}C NMR spectrum of compound **20**

3-((3-Hydroxyphenyl)(3-oxo-3-(2-(1-phenylethylidene)hydrazineyl)propyl)amino)-N'-(1-phenylethylidene)propanehydrazide ***(21)***

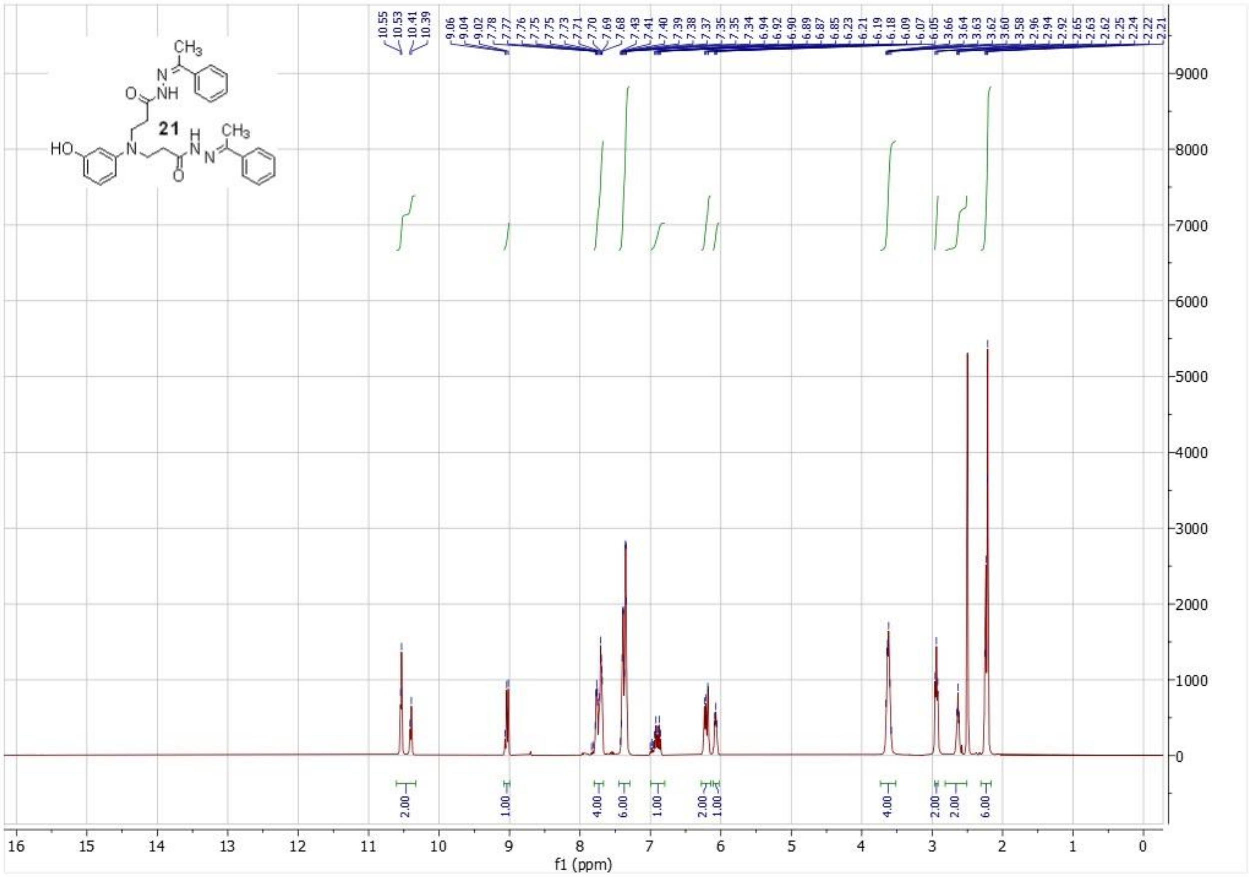

Figure S33. ^{1}H NMR spectrum of compound **21**

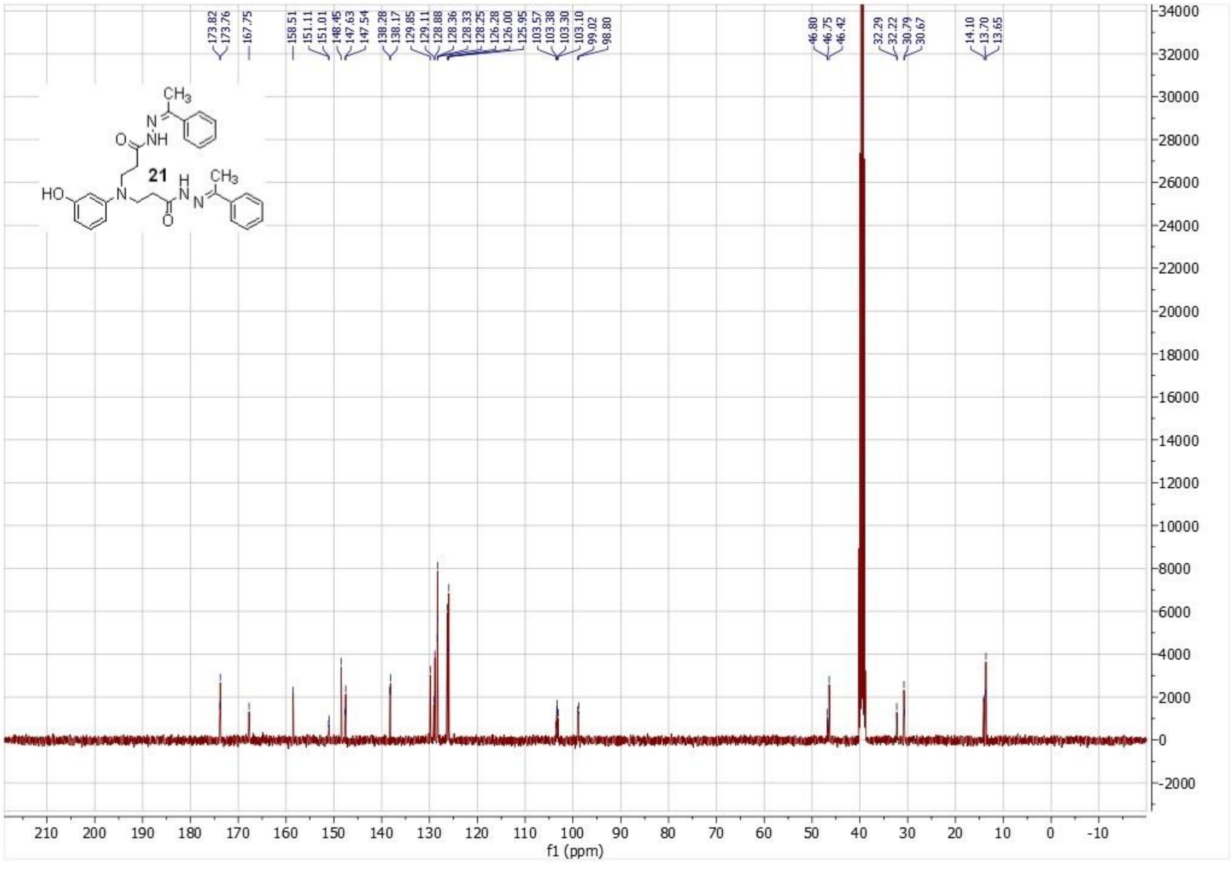

Figure S34. ^{13}C NMR spectrum of compound **21**

4,4'-(((3,3'-((3-Hydroxyphenyl)azanediyl)bis(propanoyl))bis(hydrazin-2-yl-1-ylidene))bis(ethan-1-yl-1-ylidene))dibenzenesulfonamide ***(22)***

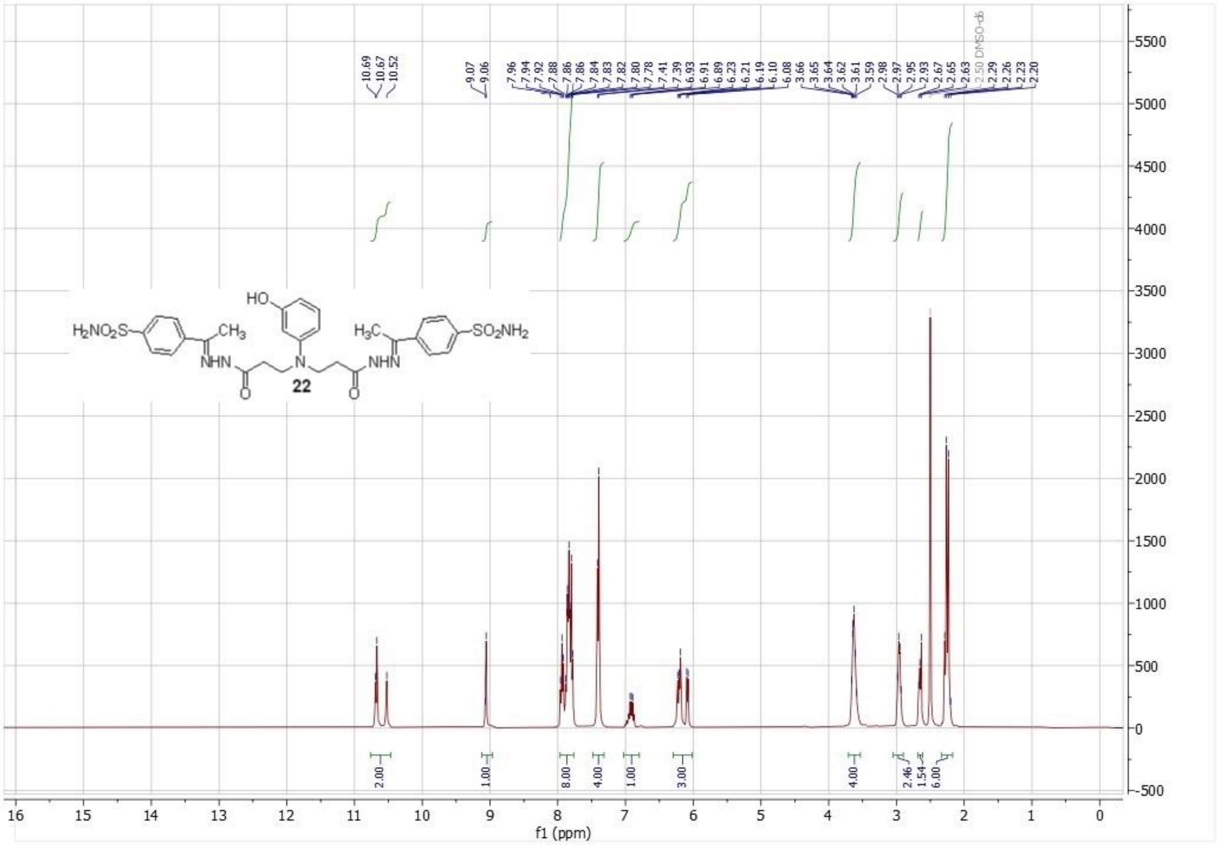

Figure S35. ^{1}H NMR spectrum of compound **22**

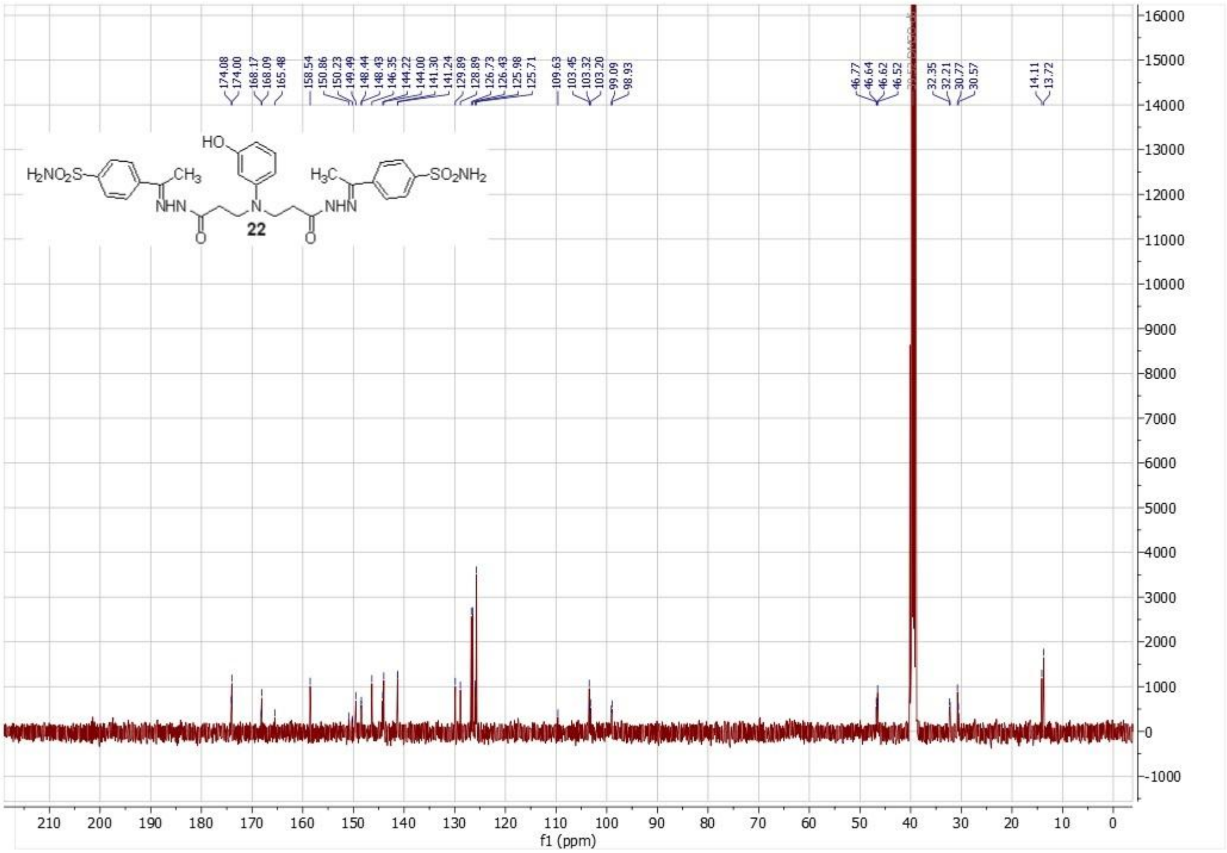

Figure S36. ^{13}C NMR spectrum of compound **22**

3,3'-((3-Hydroxyphenyl)azanediyl)bis(N-(2,5-dimethyl-1H-pyrrol-1-yl)propanamide) ***(23)***

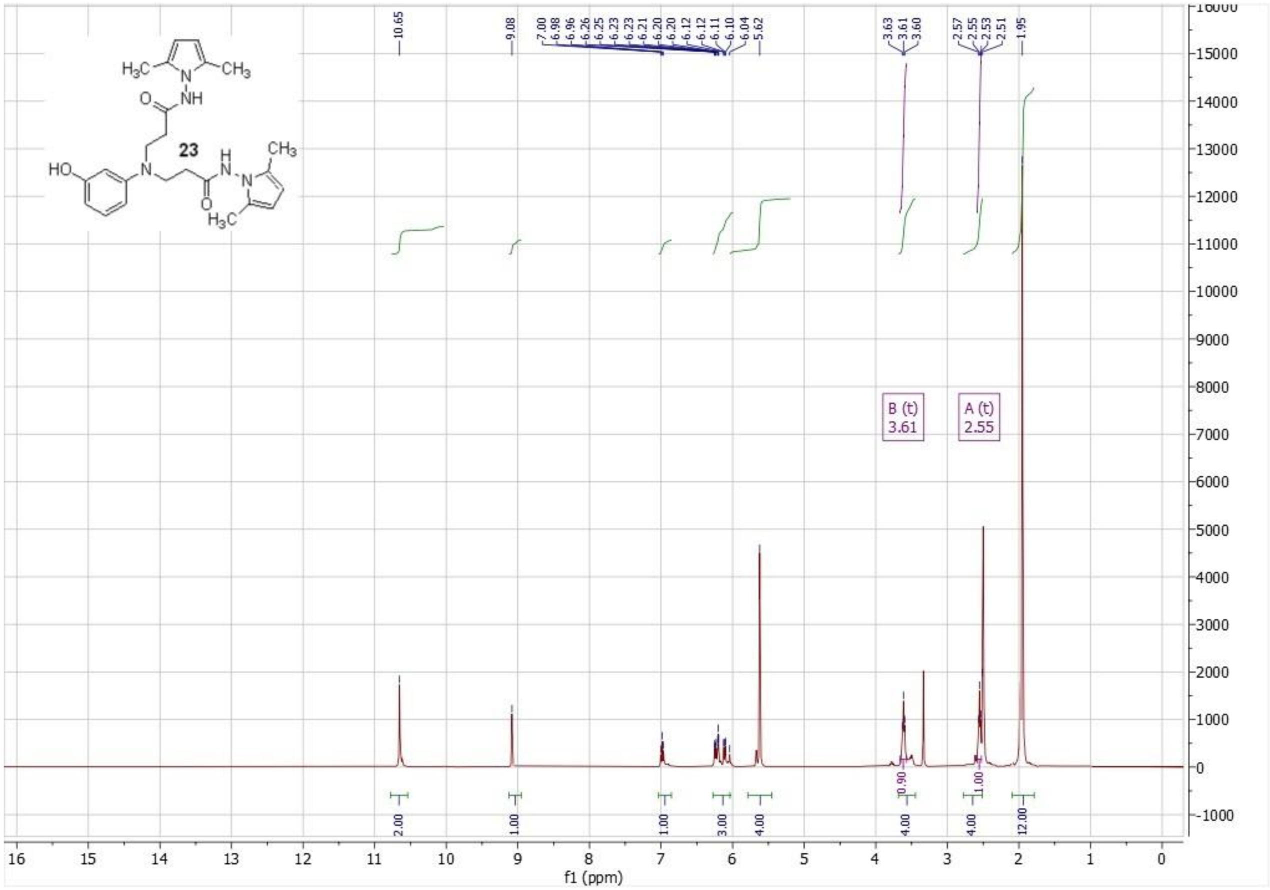

Figure S37. ^{1}H NMR spectrum of compound **23**

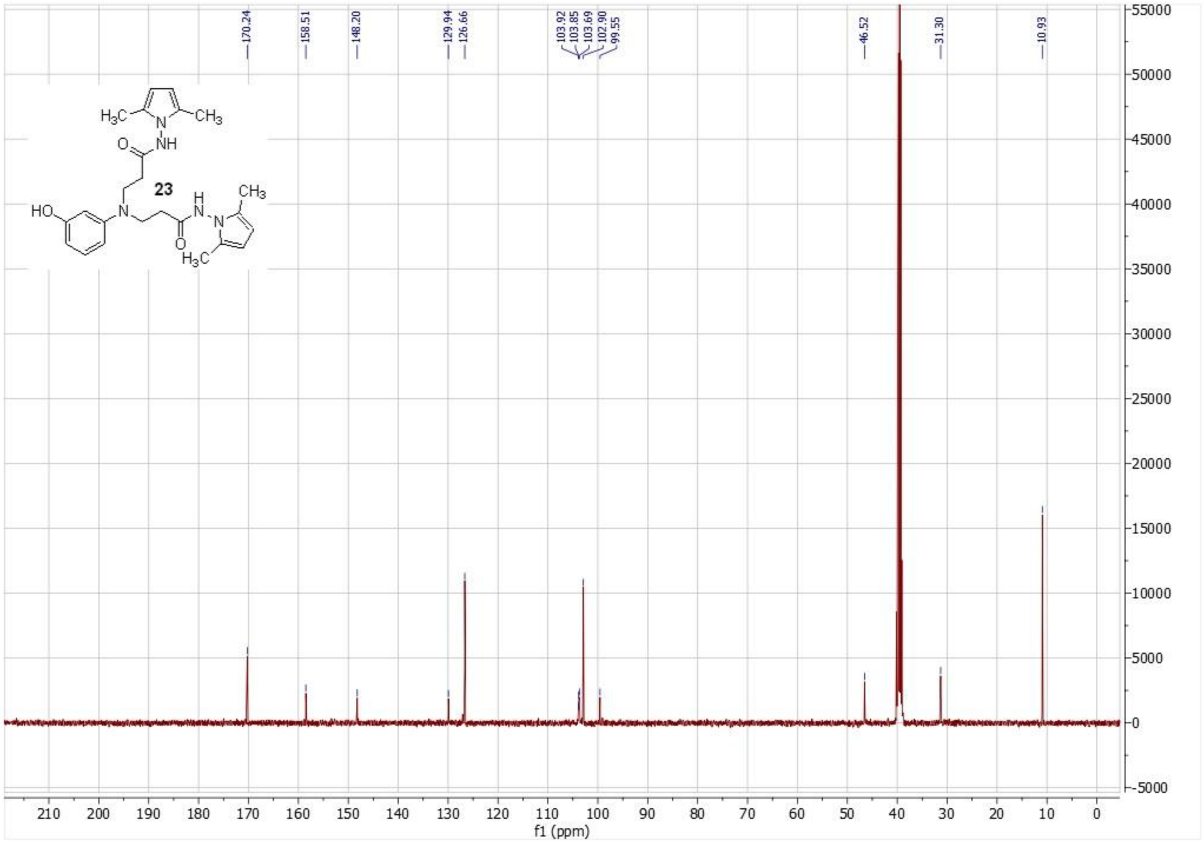

Figure S38. ^{13}C NMR spectrum of compound **23**

3,3'-((3-Hydroxyphenyl)azanediyl)bis(1-(3,5-dimethyl-1H-pyrazol-1-yl)propan-1-one) ***(24)***

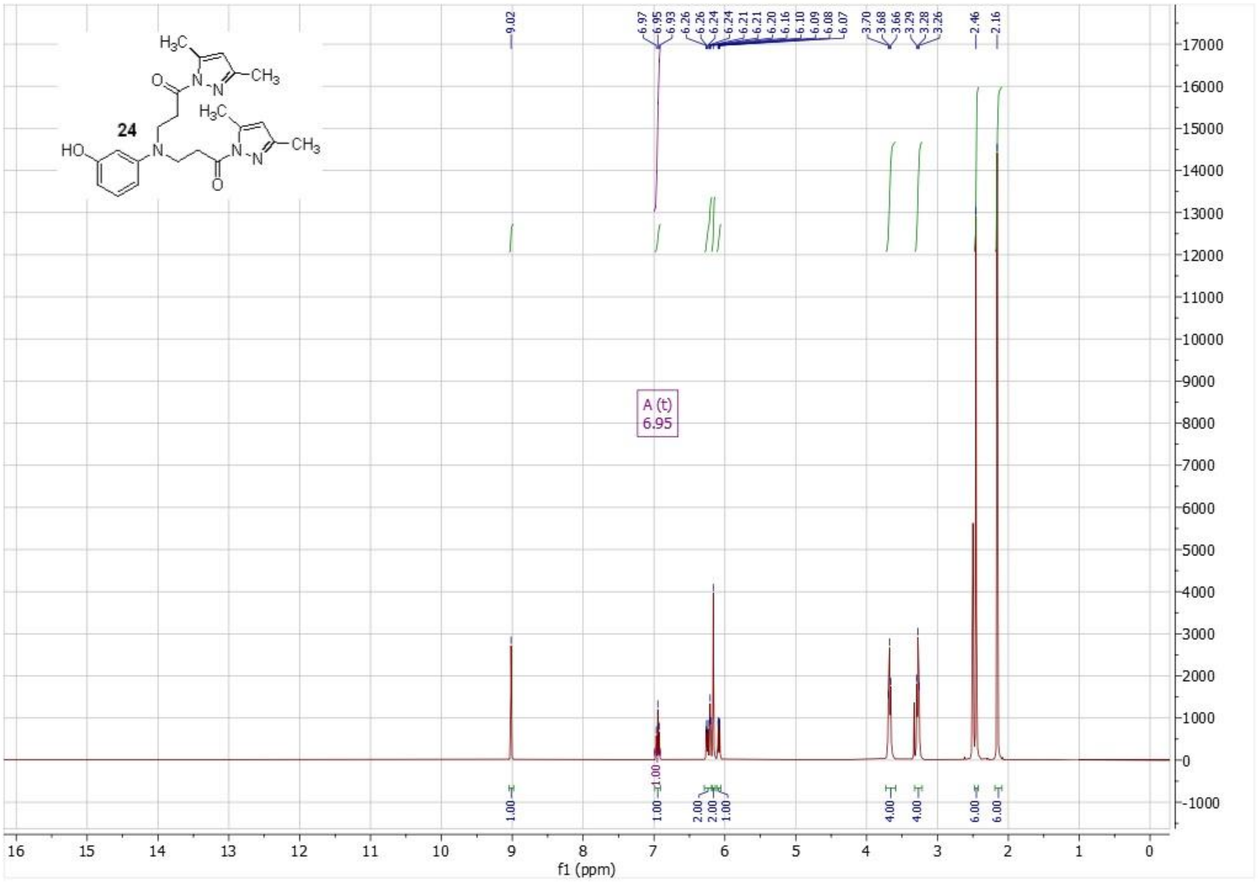

Figure S39. ^{1}H NMR spectrum of compound **24**

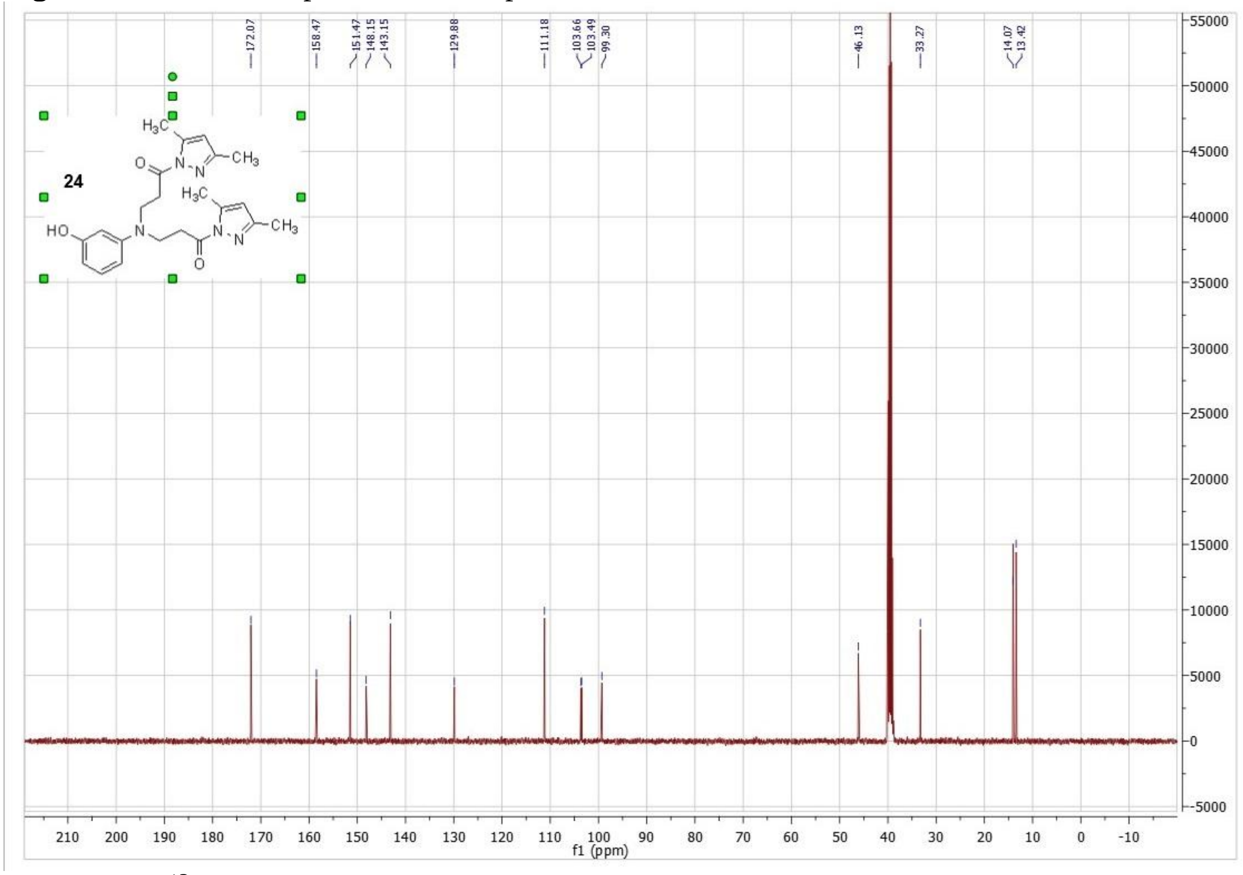

Figure S40. ^{13}C NMR spectrum of compound **24**

3,3'-((3-Hydroxyphenyl)azanediyl)bis(N'-(2-oxoindolin-3-ylidene)propanehydrazide ***(25)***

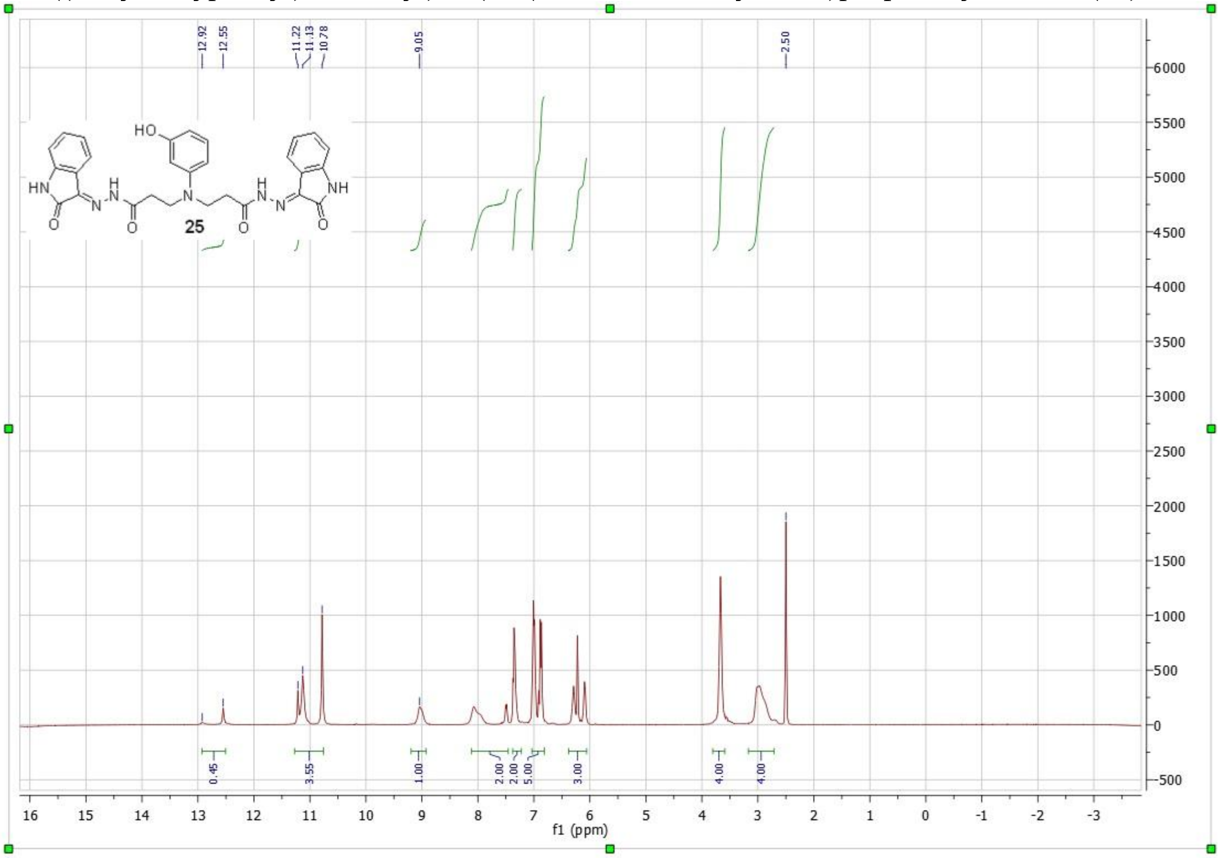

Figure S41. ^{1}H NMR spectrum of compound **25**

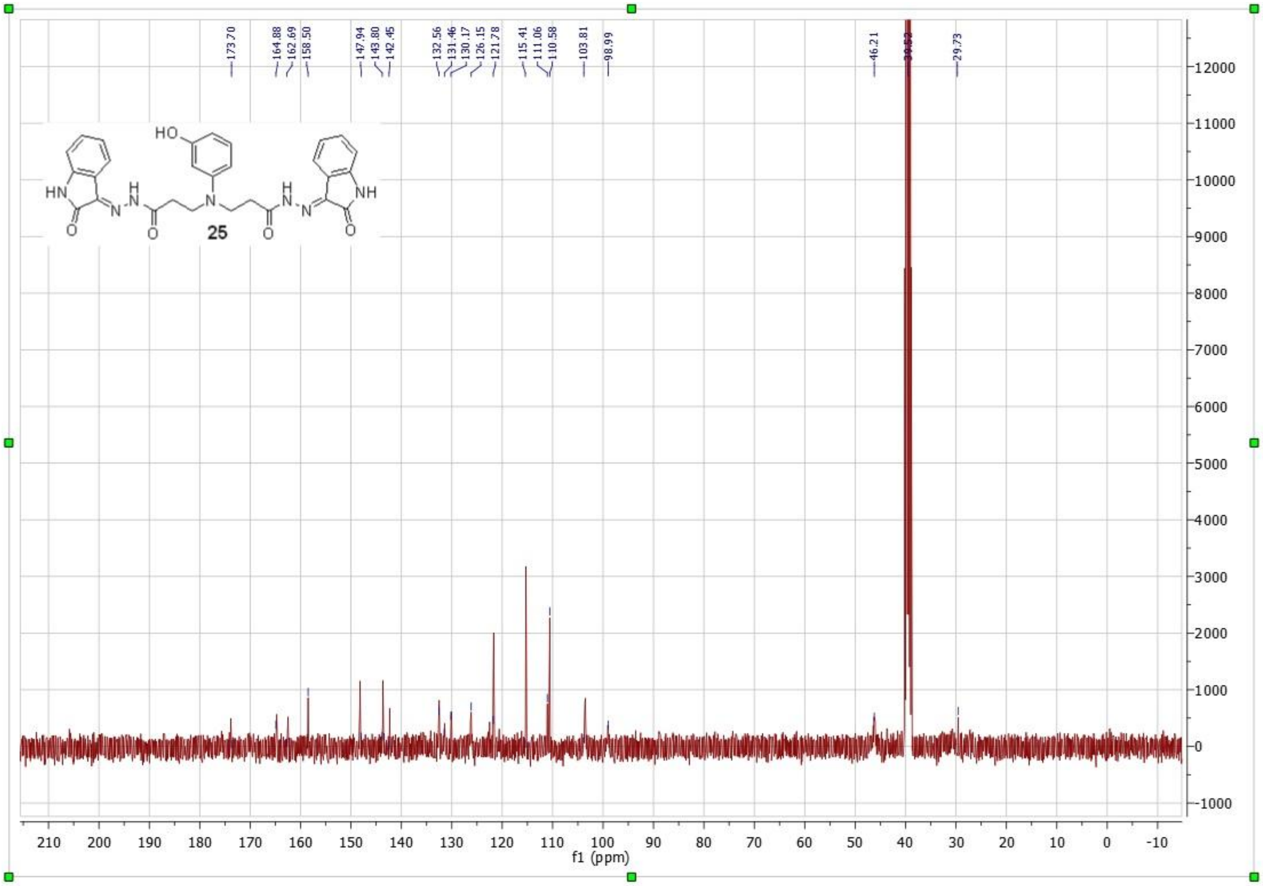

Figure S42. ^{13}C NMR spectrum of compound **25**
